# Osteosarcoma Cell and Osteosarcoma Stem Cell Potent Immunogenic Bi‐Nuclear Gallium(III) Complexes

**DOI:** 10.1002/chem.202500747

**Published:** 2025-04-23

**Authors:** Xiao Feng, Shruti Dhandore, Yu Liu, Kuldip Singh, Fabrizio Ortu, Kogularamanan Suntharalingam

**Affiliations:** ^1^ School of Chemistry University of Leicester Leicester LE1 7RH UK

**Keywords:** antitumor agents, bioinorganic chemistry, cancer, gallium, Schiff bases

## Abstract

We report the synthesis, characterization, anti‐osteosarcoma and anti‐osteosarcoma stem cells (OSC) properties (cytotoxic and immunogenic) of a series of bi‐nuclear gallium(III) complexes with tridentate Schiff base ligands and 8‐hydroxyquinoline (1–4). According to monolayer cytotoxicity studies, 1–4 display micromolar potency toward bulk osteosarcoma cells and OSCs. The most effective complex in series 2 is up to 13‐fold more potent toward OSCs than cisplatin and carboplatin (the only metallodrugs used in the clinic to treat osteosarcoma). Remarkably, the bi‐nuclear gallium(III) complexes 1–4 are significantly more potent toward 3D‐cultured sarcospheres than OSCs cultured in monolayers indicating effective penetration of the sarcosphere multicellular architecture. The bi‐nuclear gallium(III) complexes 1–4 are up to 53‐fold more potent toward sarcospheres than cisplatin and carboplatin. Mechanistic studies show that gallium(III) complex 2 kills osteosarcoma cells by caspase‐dependent apoptosis and paraptosis, leading to the release of danger‐associated molecular patterns associated with immunogenic cell death. Osteosarcoma cells and OSCs treated with gallium(III) complex 2 are effectively phagocytosed by immune cells, highlighting its immunogenic potential. As far as it is known, gallium(III) complex 2 is the first metal complex to evoke an immunogenic response toward both bulk osteosarcoma cells and OSCs.

## Graphical abstract

Current treatment options are unable to benefit the majority of osteosarcoma patients with metastasized tumors (70–80% 5‐year fatality rate). Osteosarcoma stem cells (OSCs), a small sub‐population of osteosarcoma cells with stem cell‐like properties, are thought to play a role in the spread of osteosarcoma. In this manuscript, we report the first metal complex to evoke an immunogenic response toward both bulk osteosarcoma cells and OSCs.

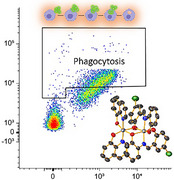

## 1 Introduction

Osteosarcoma is the most common primary bone cancer in children and adolescents.^[^ ^1^ ^]^ Osteosarcoma tends to occur in the long bones in the arms and legs and has a high tendency for metastasis.^[^ ^2^ ^]^ Reportedly, 15–20% of newly diagnosed osteosarcomas are detected with metastatic sites.^[^ ^3^ ^]^ The five‐year survival rate for osteosarcoma patients with metastasized tumors is only 20–30%, therefore there is an urgent clinical need to develop new chemotherapeutic agents that can prevent metastasis.^[^ ^4^ ^]^ Metastasis in osteosarcoma patients is heavily linked to the existence of a subpopulation of self‐renewing osteosarcoma cells called osteosarcoma stem cells (OSCs).^[^ ^5^ ^]^ The current frontline chemotherapeutic regimens used to treat osteosarcoma patients (including methotrexate, cisplatin, and doxorubicin) are believed to enrich OSC populations, increasing the possibility of metastasis.^[^ ^6^ ^]^ The implication of OSCs means that osteosarcoma treatments need to remove heterogeneous osteosarcoma populations in their entirety, including OSCs, otherwise OSC‐mediated metastasis could occur. Although studies have identified several OSC targets, an effective OSC‐active agent with low systematic toxicity has not been developed by academic‐ or industry‐led endeavors thus far.^[^ ^5^, ^7^ ^]^

We reported the first metal‐based agents to potently kill OSCs in vitro.^[^ ^8^ ^]^ Specifically, a series of gallium(III) compounds containing polypyridyl ligands were shown to kill bulk osteosarcoma cells and OSCs (in monolayer and sarcosphere cultures) within the nanomolar range.^[^ ^8^ ^]^ The rationale for investigating the anti‐OSC properties of gallium(III) compounds was based on the intrinsic ability of gallium(III) salts to accumulate in bones, and the favorable safety profiles observed for gallium(III) compounds that have undergone human clinical trials (such as KP46).^[^ ^9^ ^]^ The most effective gallium(III) complex in the series was over 400‐fold more potent than cisplatin toward methotrexate‐resistant OSCs, and significantly less toxic toward a panel of noncancerous cells of various tissue types (lung, breast, skin, and kidney).^[^ ^8^ ^]^ Mechanistic studies showed that the lead gallium(III) complex induced apoptotic osteosarcoma cell death by entering the nucleus and damaging genomic DNA.^[^ ^8^ ^]^

Following this work, we developed “second generation” gallium(III)‐polypyridyl complexes containing salicylic acid and diflunisal, nonsteroidal anti‐inflammatory drugs (NSAIDs), with improved anti‐OSC properties.^[^ ^10^ ^]^ NSAIDs are established inhibitors of cyclooxygenase‐2 (COX‐2).^[^ ^11^ ^]^ COX‐2 catalyses the formation of prostaglandin (an inflammation mediator).^[^ ^12^ ^]^ COX‐2 is an established marker in osteosarcoma and its inhibition is widely thought to be a viable method of improving therapeutic outcomes.^[^ ^13^ ^]^ Histological studies have shown that COX‐2 expression in osteosarcoma patients correlates with tumor grade, metastasis potential, and lower survival rates.^[^ ^14^ ^]^ COX‐2 expression is elevated in OSCs (compared to bulk osteosarcoma cells) and plays a vital role in OSC maintenance.^[^ ^15^ ^]^ The diflunisal‐bearing gallium(III) complex displayed significantly higher monolayer and sarcosphere OSC potency (up to three orders of magnitude) than clinically approved osteosarcoma drugs used in frontline (doxorubicin and cisplatin) and secondary (etoposide, ifosfamide, and carboplatin) treatments.^[^ ^10a^ ^]^ Mechanistic studies showed that the diflunisal‐bearing gallium(III) complex killed osteosarcoma cells by simultaneously inhibiting COX‐2 and damaging nuclear DNA.^[^ ^10a^ ^]^ The diflunisal‐bearing gallium(III) complex was also incorporated into a clinically approved, spherical polymeric nanoparticle system (methoxy poly(ethylene glycol)‐*b*‐poly(D,L‐lactic‐co‐glycolic) acid) with the aim of improving delivery to osteosarcoma sites.^[^ ^16^ ^]^ The optimized nanoparticle formulation displayed tenfold better activity toward OSCs than the payload.^[^ ^16^ ^]^ The nanoparticle formulation also exhibited up to 5645‐fold greater potency toward OSCs than frontline anti‐osteosarcoma drugs, doxorubicin and cisplatin.^[^ ^16^ ^]^ Furthermore, the nanoparticle formulation evoked a similar mechanism of action as the payload, which bodes well for further translation.

Immunotherapy, where the immune system is stimulated to destroy tumors, has emerged as a feasible alternative to conventional cytotoxic cancer therapies over the last decade and could provide long‐term durable therapeutic outcomes for osteosarcoma patients.^[^ ^17^ ^]^ The clinical development of osteosarcoma‐directed immunotherapy has largely focused on monoclonal antibodies and native biomolecules that can block protein‐protein interactions between T‐cell checkpoint receptors and their cognate ligands.^[^ ^17^, ^18^ ^]^ Antigen‐specific immunotherapy such as adoptive T‐cell or chimeric antigen receptor (CAR)‐T‐cell therapy has also shown some promise against osteosarcoma.^[^ ^17^, ^18^ ^]^ Given that osteosarcomas are considered low immunogenic tumors, the general effectiveness of current biologic approaches is somewhat limited. These biological approaches prime immune cells to seek and destroy osteosarcoma cells by recognizing protein(s) on their surface. As bulk osteosarcoma cells and OSCs express different surface protein arrays, and immunological approaches are inherently biased toward more differentiated bulk osteosarcoma cells, current biologic approaches are largely ineffective against OSCs. As far as we are aware no methods have been reported to prime immune cells to target OSCs. Mifamurtide, a synthetic analog of muramyl dipeptide, is used as an immunomodulator to treat osteosarcoma patients (and reduces the risk of dying by 13% over 5 years); however, its impact on OSCs is not reported.^[^ ^19^ ^]^ In general, small molecule immuno‐chemotherapeutics offer distinct advantages over biologic approaches including; higher feasibility, opportunities for intracellular targeting, flexible formulation and dosing options to overcome pharmacokinetic and pharmacodynamics challenges, and lower costs.^[^ ^20^ ^]^ The current batch of small molecules undergoing investigation as OSC‐specific agents are typically organic and display no immunological features.^[^ ^21^ ^]^ These agents induce OSC death via cytotoxic mechanisms only, which are susceptible to acquired resistance mechanisms.

Although the potency of various gallium(III) complexes toward bulk osteosarcoma cells and OSCs has been investigated, their immunogenic potential remains unexplored.^[^ ^8^, ^10^ ^]^ In this study, we report the preparation, characterization, anti‐osteosarcoma and anti‐OSC properties (both cytotoxic and immunogenic) of a series of bi‐nuclear gallium(III) complexes comprising of tridentate Schiff base ligands and 8‐hydroxyquinoline. The Schiff base ligands contain two hard oxygen donor atoms to stabilize the hard gallium(III) metal centres. The use of 8‐hydroxyquinoline was inspired by the structure of KP46, a gallium(III) complex with three 8‐hydroxyquinoline units, that has progressed to human clinical trials.^[^ ^9^ ^]^ Considering the inherent ability of gallium salts to amass in bones, we envisage that the bi‐nuclear gallium(III) complexes could effectively target bone material. Indeed our studies show that the bi‐nuclear gallium(III) complexes are readily absorbed onto bone material and display very promising bulk osteosarcoma cell and OSC activity in in vitro systems (both monolayer and 3D). Moreover, the most effective complex within the series displays unprecedented immunogenic potential. To the best of our knowledge, the aforementioned gallium complex reported in this study is the first metal complex to evoke an immunogenic response toward both bulk osteosarcoma cells and OSCs.

## 2 Results and Discussion

### 2.1 Synthesis and Characterization of Bi‐Nuclear Gallium(III) Complexes

**Figure 1:**
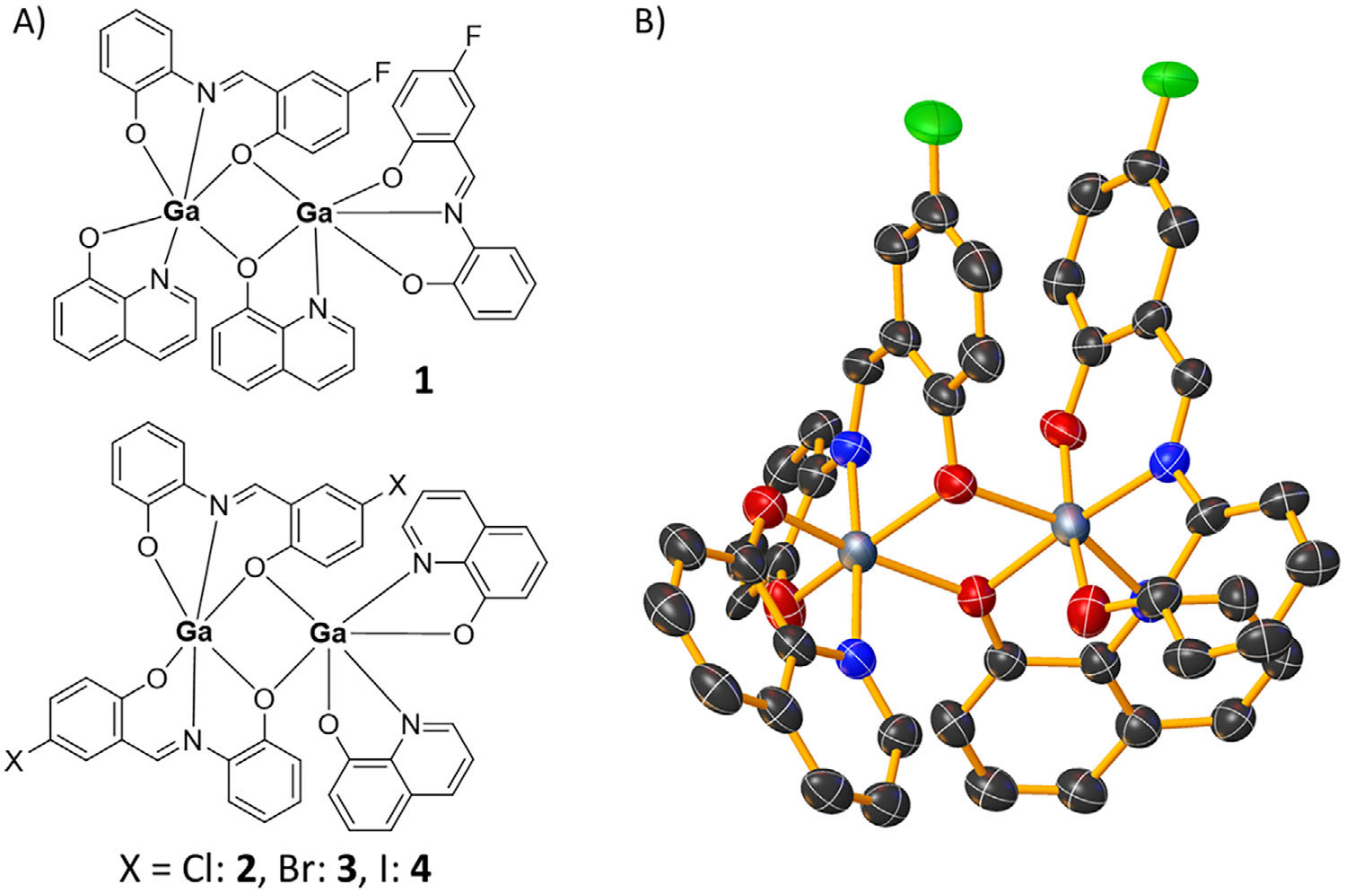
A) Chemical structure of the bi‐nuclear gallium complexes **1**–**4** comprising of tridentate Schiff base ligands (**L^1^**‐**L^4^**) and 8‐hydroxyquinoline moieties. B) X‐ray structure of **1**. Ellipsoids are shown at 50% probability, H atoms and co‐crystalizing solvent molecules have been omitted for clarity. C in black, N in dark blue, O in red, F in green, and Ga in grey.

Four bi‐nuclear gallium compounds (**1**‐**4**) were prepared with various tridentate Schiff base ligands (**L^1^**‐**L^4^**) and 8‐hydroxyquinoline (structures depicted in Figure 1A). The Schiff base ligands (**L^1^**‐**L^4^**) were prepared by reacting equimolar amounts of 2‐aminophenol and the corresponding salicylaldehyde (5‐fluorosalicylaldehyde for **L^1^**, 5‐chlorosalicylaldehyde for **L^2^**, 5‐bromosalicylaldehyde for **L^3^**, 5‐iodosalicylaldehyde for **L^4^**) in methanol. The Schiff base ligands (**L^1^**‐**L^4^**) were isolated as yellow or orange solids in reasonable to good yields (44–77%), and characterized by ^1^H and ^19^F{^1^H} NMR, infrared spectroscopy, and ESI mass spectrometry (see Figures S1–S11, Supporting Information). Characteristic imine proton signals at 8.95–8.98 ppm in the ^1^H NMR spectra and C═N bands at 1620–1632 cm^−1^ in the IR spectra for **L^1^**‐**L^4^** confirmed formation of the imine functionality (Figures S1, S3–S5, andS7, Supporting Information). Disappearance for the aldehyde proton peak (10.17–10.25 ppm) associated to the various salicylaldehyde starting materials established full conversion to the Schiff base ligands (**L^1^**‐**L^4^**) (Figures S1 andS3–S6, Supporting Information). The bi‐nuclear gallium(III) complexes (**1**‐**4**) were synthesized by reacting GaCl_3_ with equimolar amounts of **L^1^**‐**L^4^** and 8‐hydroxyquinoline in the presence of piperidine in ethanol. The bi‐nuclear gallium(III) complexes (**1**‐**4**) were isolated as orange solids in reasonable yields (43–58%), and characterized by ^1^H and ^19^F{^1^H} NMR, infrared spectroscopy, ESI mass spectrometry and elemental analysis (see Figures S12–S21, Supporting Information). The aromatic signals associated with the ^1^H NMR spectra of **1**–**4** shifted relative to **L^1^**‐**L^4^**, indicative of metal coordination (Figures S12 and S14–S16, Supporting Information). The fact that only one set of ^1^H NMR signals were observed for the **L^1^**‐**L^4^** and 8‐hydroxyquinoline moieties in **1**–**4** suggests that a single regioisomer was isolated in each case. The IR spectra of **1**–**4** displayed C═N stretching bands at relatively lower frequencies than the corresponding Schiff base ligands (**L^1^**‐**L^4^**), further confirming coordination of gallium to **L^1^**‐**L^4^** (Figure S17, Supporting Information). Distinctive molecular ion peaks corresponding to **1**–**4** with the appropriate isotopic pattern was observed in the positive mode of the high‐resolution ESI mass spectra (*m/z* = 887.0568 amu for [**1**+H]^+^; 918.9961 amu for [**2**+H]^+^; 1008.8945 amu for [**3**+H]^+^; 1102.8684 amu for [**4**+H]^+^), providing further evidence for product formation (Figures S18–S21, Supporting Information). The purity of the bulk solid of **1–4** was confirmed by elemental analysis (see Supporting Information).

Single crystals of **1** and **2** suitable for X‐ray diffraction studies were obtained by slow diffusion of diethyl ether into an acetonitrile solution of **1** and **2** (CCDC 2419225–2419226, Table S1, Supporting Information; Figure 1B; Figure S22, Supporting Information).^[^ ^22^ ^]^ Selected bond distance and bond angle data for **1** are presented in Table S2 (Supporting Information). The data obtained for **2** was insufficient to reliably determine bond parameters; however, the connectivity of the bi‐nuclear gallium(III) complex could be resolved. The structures of **1** and **2** consist of two distorted octahedral gallium(III) centres, two Schiff base ligands (**L^1^** for **1** and **L^2^** for **2**), and two 8‐hydroxyquinoline units. As depicted in Figure 1B and Figure S22 (Supporting Information), each of the gallium(III) centres in **1** and **2** display different coordination environments. For **1**, one of the Schiff base ligands **L^1^** (via the phenolato oxygen atom on the fluorophenyl moiety) and one of the 8‐hydroxyquinoline ligands bridge the two gallium(III) centres. For **2**, both of the Schiff base ligands **L^2^** bridge the two gallium(III) centres (via different phenolato oxygen atoms on the chlorophenyl and unsubstituted phenyl moieties). The difference in ligand arrangement around the two gallium(III) centres could be partly attributed to the favorable intramolecular offset *π–π* interaction between the fluorophenyl moieties in **1** (dihedral angle, Φ of 20.1(3)° between the least‐squares planes and a centroid‐centroid distance, *d* of 3.665(5) Å), which is not observed for the chlorophenyl moieties in **2**. This is plausible as chlorophenyl moieties (compared to fluorophenyl moieties) are less polarized and thus less likely to shift the quadrupole moment of the associated phenyl ring to promote intramolecular *π–π* interactions. The relatively short Ga‐Ga distance in **1** (3.1936(6) Å) could also be partly attributed to the abovementioned intramolecular *π–π* interactions. Overall the Ga–O (bridging and terminal) and Ga–N bond distances determined for **1** are consistent with the bond parameters for a structurally related bi‐nuclear gallium(III) complex.^[^ ^23^ ^]^ The structures of **3** and **4** are likely to be similar to those obtained for **2** (as depicted in Figure S22, Supporting Information) based on the electronegativity and size of the bromine and iodine substituents on **L^3^** and **L^4^**, respectively.

### 2.2 Solution Stability of Bi‐Nuclear Gallium(III) Complexes

The lipophilicity of the bi‐nuclear gallium(III) complexes was determined by measuring the extent to which **1**–**4** partitioned between octanol and water using UV–vis spectroscopy. The experimentally determined Log P values varied from 1.51 ± 0.001 to 2.10 ± 0.03 (Table S3, Supporting Information). The nature of the substituent on the Schiff base ligands (**L^1^**‐**L^4^**) attached to the gallium(III) centers in **1**–**4** influences hydrophobicity. The lipophilicity increased along with the size of the substituent, LogP value for **1** (F) < **2** (Cl) < **3** (Br) < **4** (I). Overall, the LogP values for **1**–**4** suggests that the bi‐nuclear gallium(III) complexes should be adequately soluble in aqueous media to conduct biological studies and highly cell permeable.

**Figure 2:**
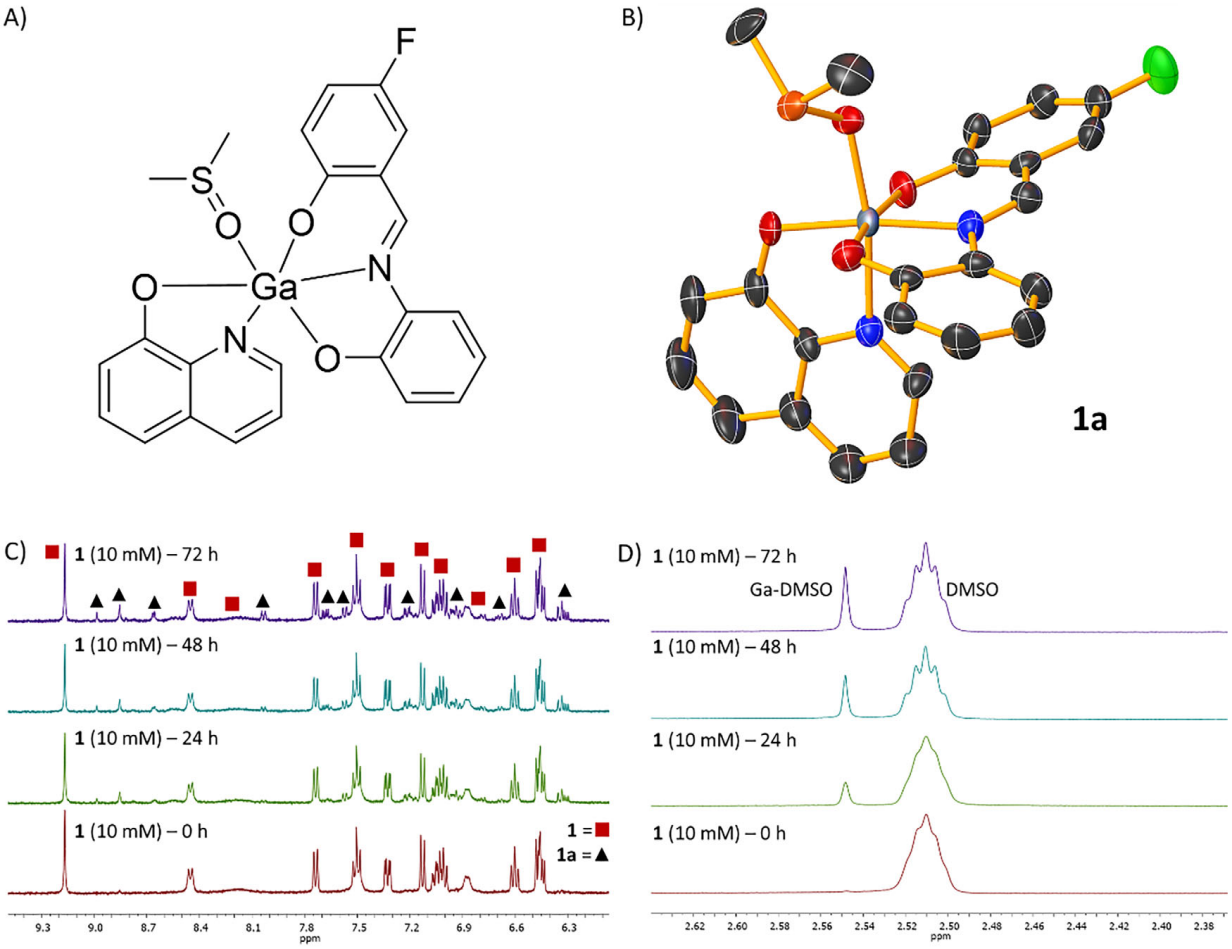
^1^H NMR spectra of **1** (10 mm) in D_2_O:DMSO‐*d*_6_ (9:1) over the course of 72 h. A) aromatic region and B) aliphatic region. C) Chemical structure of the mono‐nuclear gallium(III) complex **1a** formed in DMSO:H_2_O (1:1). D) X‐ray structure of **1a**. Ellipsoids are shown at 50% probability, H atoms have been omitted for clarity. C in black, N in dark blue, O in red, S in orange, F in green, and Ga in gray.

The solution stability of **1**–**4** was assessed by time course UV–vis and ^1^H NMR spectroscopy and ESI mass spectrometry experiments. In DMSO, the UV–vis trace associated with **1**–**4** (50 µm) remained unchanged over the course of 24 h at 37 °C (Figure S23, Supporting Information), indicative of stability. The ^1^H NMR spectra of **1**–**4** (10 mm) in DMSO‐*d*_6_ remained unaltered over the course of 72 h at 37 °C (Figures S24–S27, Supporting Information), proving that **1**–**4** do not undergo structural modifications in DMSO. The ESI mass spectra of **1**–**4** (0.5 mm) in DMSO each displayed a distinctive molecular ion peak corresponding to **1**–**4** throughout the course of 24 h at 37 °C (Figures S28–S31, Supporting Information), further confirming the stability of **1**–**4** in DMSO. The stability of **1**–**4** in DMSO bodes well for conducting biological studies as most assays involve solubilizing the test compound in DMSO prior to dilution in the appropriate biological media. In H_2_O:DMSO (200:1), the UV–vis band in 400–450 nm region associated with the respective metal perturbed π–π* transitions within **1**–**4** (50 µm) decreased slightly (by 9–16%) over the course of 24 h at 37 °C (Figure S32, Supporting Information) implicative of some structural changes. To decipher the precise nature of the structural changes we attempted to grow single crystals suitable for X‐ray diffraction studies from concentrated solutions of **1**–**4** in DMSO:H_2_O (1:1). Crystallization was successful for **1** and **3**. In each case the structure revealed a mono‐nuclear gallium(III) complex (**1a** and **3a**) comprising of the corresponding Schiff base ligand (**L^1^** or **L^3^**), 8‐hydroxyquinoline, and a DMSO molecule (bound to the gallium centre via the oxygen atom) (CCDC 2 419 224 and 2 419 227, Tables S4–S6, Supporting Information; Figure 2A,B; Figure S33, Supporting Information).^[^ ^22^ ^]^ This suggests that the bridging Ga‐O‐Ga bonds in **1** and **3** are somewhat prone to cleavage in the presence of H_2_O and this can lead to the formation of an adduct with DMSO. The ^1^H NMR spectra of **1**–**4** (10 mm) in D_2_O:DMSO‐*d*_6_ (9:1) over the course of 72 h at 37 °C was dominated by signals associated to the intact bi‐nuclear gallium(III) complexes **1**–**4** however additional signals corresponding to the mono‐nuclear gallium(III)‐DMSO complexes **1a**‐**4a** were also observed (Figure 2C,D; Figures S33–S37, Supporting Information). Collectively, the UV–vis, XRD, and ^1^H NMR analysis indicate that in H_2_O:DMSO solutions, the bi‐nuclear gallium(III) complexes **1**–**4** are largely stable but can undergo structural transformation to form the corresponding mono‐nuclear gallium(III)‐DMSO complexes **1a**‐**4a** in small amounts. Additional time course UV–vis spectroscopy studies in MEGM:DMSO (200:1) revealed that that the UV–vis bands associated to **1**–**4** (50 µm) decreased over the course of 24 h (Figure S38, Supporting Information). The changes are similar, but more pronounced, compared to those observed in H_2_O:DMSO (200:1) (Figure S32, Supporting Information). This implies that **1**–**4** undergo structural changes in MEGM, and are susceptible to form the corresponding mono‐nuclear gallium(III)‐DMSO complexes **1a**‐**4a**. The observed structural changes in biologically relevant solutions could negatively impact the in vivo performance of **1**–**4**.

### 2.3 Bone Mineral Absorption Studies

Given the inherent ability of gallium‐containing compounds to accumulate in bones in vivo, we determined the ability of the bi‐nuclear gallium(III) complexes **1**–**4** to be absorbed onto bone minerals using a hydroxyapatite model.^[^ ^24^ ^]^ The bi‐nuclear gallium(III) complexes **1**–**4** (0.1 mm) were incubated with hydroxyapatite (10 mg) in HEPES buffer at 37 °C and agitated (200 rpm). At certain time points over a 48 h incubation period, the amount of **1**–**4** in solution (unbound to hydroxyapatite) was determined by ICP‐MS and used to generate depletion plots (Figure S39, Supporting Information). The bi‐nuclear gallium(III) complexes **1** and **2** displayed similar absorption profiles, the amount of gallium absorbed onto hydroxyapatite steadily increased over 48 h, reaching a maximum of 34–39% absorption. The bromine‐ and iodine‐containing gallium(III) complexes **3** and **4** displayed rapid absorbed onto hydroxyapatite in the first 1 h before a relatively steady absorption profile was reached. Notably, the amount of **4** (71%) absorbed onto hydroxyapatite after 1 h incubation was significantly higher than **3** (42%). There was also some evidence of gallium release at longer incubation times (3–24 h), suggesting that binding of **3** and **4** onto hydroxyapatite is reversible. Overall the bone mineral absorption studies indicate that the bi‐nuclear gallium(III) complexes have reasonable affinity for hydroxyapatite, with the iodine‐containing complex **4** displaying the greatest absorption over 48 h.

### 2.4 Cytotoxicity Toward Bulk Osteosarcoma Cells and Osteosarcoma Stem Cells

The cytotoxicity of the bi‐nuclear gallium(III) complexes **1**–**4** toward bulk osteosarcoma cells (U2OS) and OSC‐enriched cells (U2OS‐MTX) was assessed using the MTT [3‐(4,5‐dimethylthiazol‐2‐yl)‐2,5‐diphenyltetrazolium bromide] assay. The IC_50_ values (concentration required to reduce cell viability by 50%) were derived from dose‐response curves (Figures S40–S43, Supporting Information) and are summarized in Table 1. The bi‐nuclear gallium(III) complexes **1**–**4** displayed micromolar potency toward U2OS and U2OS‐MTX cells, with slightly higher toxicity toward bulk osteosarcoma cells than OSC‐enriched cells. The chlorine‐containing gallium(III) complex **2** displayed the lowest IC_50_ values toward U2OS and U2OS‐MTX cells within the series. The chlorine‐containing complex **2** was fourfold and 38‐fold more potent than cisplatin and carboplatin toward U2OS cells (*p* < 0.05, *n* = 18) and fourfold and 13‐fold more potent than cisplatin and carboplatin toward U2OS‐MTX cells (*p* < 0.05, *n* = 18).^[^ ^8^, ^10^ ^]^ It should be noted that although **2** displayed similar potency toward U2OS cells as salinomycin (a cancer stem cell‐potent positive control), **2** was significantly less potent toward U2OS‐MTX cells than salinomycin.^[^ ^8^, ^10^ ^]^ Control cytotoxicity studies were conducted with the Schiff base ligands (**L^1^**‐**L^4^**) and Ga(NO_3_)_3_. These studies showed that **L^1^**‐**L^4^** were significantly less potent toward U2OS and U2OS‐MTX cells than their corresponding bi‐nuclear gallium(III) complexes **1**–**4** (*p* < 0.05, *n* = 18, Table S7 and Figures S44–S47, Supporting Information). Ga(NO_3_)_3_ was nontoxic toward U2OS and U2OS‐MTX cells (IC_50_ value > 100 µm).^[^ ^14^ ^]^ This suggests that the potency of **1**–**4** toward bulk osteosarcoma cells and OSCs is likely to result from the intact gallium(III) bi‐nuclear complexes rather than their individual components.

**Table 1:** IC_50_ values of **1**–**4**, salinomycin, cisplatin, and Ga(NO_3_)_3_ against U2OS cells, U2OS‐MTX cells, and U2OS‐MTX sarcospheres.

Compound	U2OS IC_50_ [µM]^[^ ^a^ ^]^	U2OS‐MTX IC_50_ [µM]^[^ ^a^ ^]^	OSC‐sarcosphere IC_50_ [µM]^[^ ^b^ ^]^
**1**	5.71 ± 0.04	10.74 ± 0.39	0.43 ± 0.003
**2**	4.10 ± 0.19	8.70 ± 0.38	0.48 ± 0.11
**3**	5.07 ± 0.21	13.71 ± 0.01	0.93 ± 0.13
**4**	4.93 ± 0.35	9.65 ± 0.83	1.04 ± 0.02
Ga(NO_3_)_3_^a^	> 100	> 100	24.94 ± 0.19
Cisplatin^[a]^	16.30 ± 0.50	33.87 ± 3.71	16.49 ± 0.20
carboplatin^[a]^	157.50 ± 2.21	114.98 ± 2.31	22.77 ± 0.09
salinomycin^[a]^	6.09 ± 1.06	1.49 ± 0.26	4.70 ± 0.08

^[a]^ Determined after 72 h incubation (mean of three independent experiments ± SD).

^[b]^ Determined after 10 days incubation (mean of three independent experiments ± SD).

^[c]^ Reported in references^[^ ^8^ ^]^ and^[^ ^10a^ ^]^.

### 2.5 Activity Toward Sarcospheres

**Figure 3:**
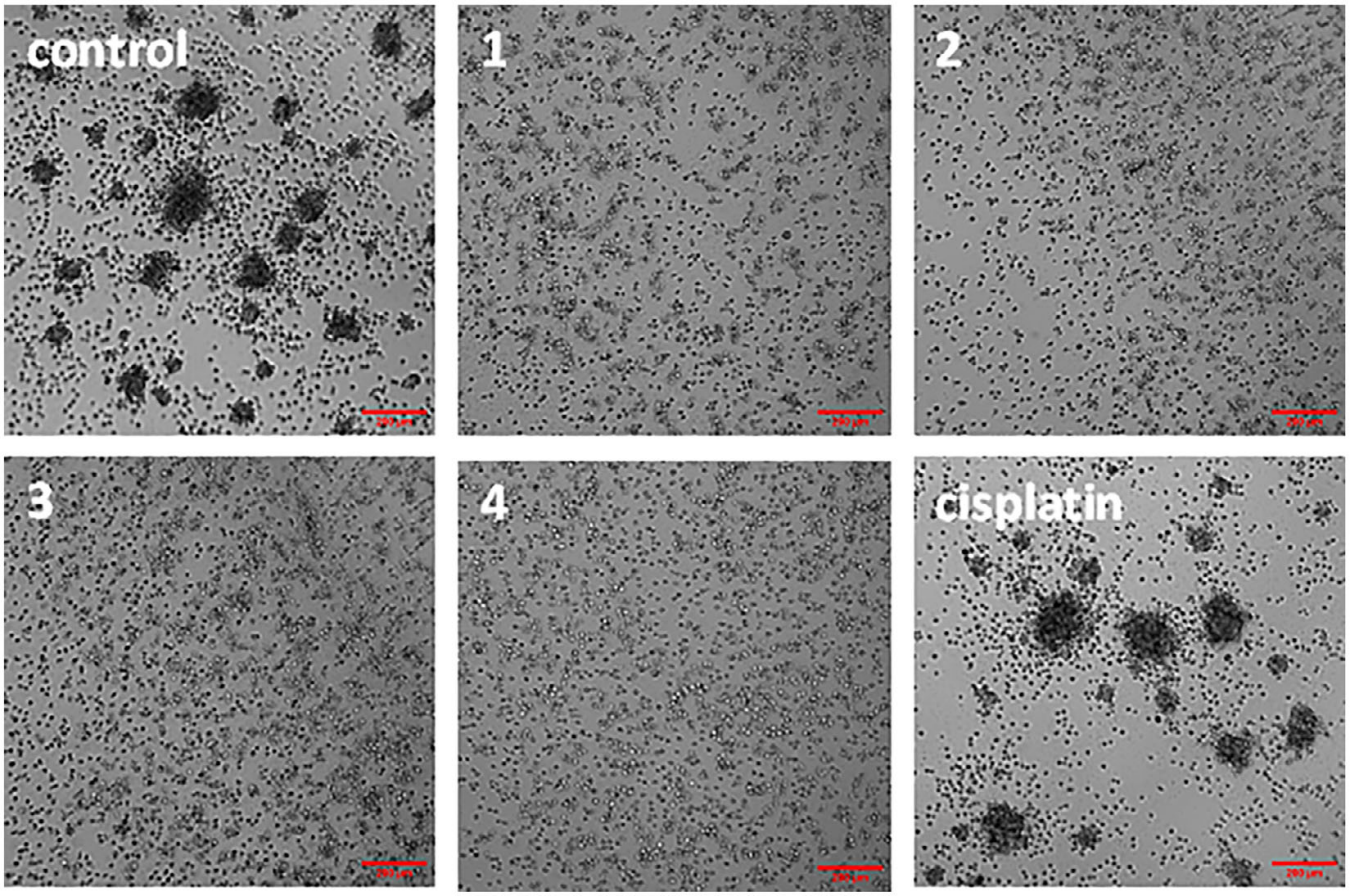
Representative bright‐field images (× 10) of U2OS‐MTX sarcospheres in the absence and presence of **1**–**4** or cisplatin at their respective IC_20_ values for 10 days.

Given the promising potency of the bi‐nuclear gallium(III) complexes **1**–**4** toward osteosarcoma cells cultured in monolayers, their activity toward 3D‐cultured sarcospheres (also called osteospheres) was determined. When U2OS‐MTX cells are cultured in low‐attachment, serum‐free conditions, sarcospheres are formed. Sarcospheres are irregularly shaped collections of OSCs that are reasonable physical mimics of osteosarcomas in vivo. Incubation of single cell suspensions of U2OS‐MTX cells with **1**–**4** (at their corresponding IC_20_ value for 10 days) markedly reduced the formation of sarcospheres compared to untreated control cells (Figure 3). Cisplatin (IC_20_ value, 10 days) only slightly reduced the formation of sarcospheres compared to untreated control cells (Figure 3), whereas salinomycin (IC_20_ value, 10 days) disrupted the formation of sarcospheres to a similar extent as **1**–**4** (Figure S48, Supporting Information). The colorimetric resazurin‐based reagent, TOX8 was used to determine the effect of **1**–**4** on sarcosphere viability. Dose‐response curves indicated that **1**–**4** displayed low micromolar or sub‐micromolar potency toward sarcospheres (Table 1; Figure S49, Supporting Information). According to the IC_50_ values obtained from the cell and sarcosphere viability studies (Table 1), **1**–**4** were significantly more potent toward 3D‐cultured sarcospheres than U2OS‐MTX cells grown in monolayer cultures (up to 25‐fold). This is highly atypical as most anticancer drug candidates tend to be more potent toward cells grown in monolayer systems than the same cells cultured as spheroids. This result implies that **1**–**4** are able to effectively penetrate the multicellular architecture of sarcospheres, which is an attractive feature with respect to in vivo translation. Notably, **1**–**4** exhibited significantly higher potency toward sarcospheres than cisplatin (up to 38‐fold, *p* < 0.05), carboplatin (up to 53‐fold, *p* < 0.05), salinomycin (up to 11‐fold, *p* < 0.05), and Ga(NO_3_)_3_ (up to 58‐fold, *p* < 0.05).^[^ ^8^, ^10^ ^]^

### 2.6 Cellular Uptake Studies

To provide insight into the mechanism of action of the bi‐nuclear gallium(III) complexes, cellular uptake studies were carried out. U2OS cells were incubated with **1**–**4** (5 µm for 24 h) and the internalized gallium content was determined by inductively coupled plasma mass spectrometry (ICP‐MS) (Figure S50, Supporting Information). The bi‐nuclear gallium(III) complexes **1**–**4** were readily taken up by U2OS cells, with whole cell uptake ranging from 56.46 ± 1.60 ppb of Ga/ million cells for **3** to 73.77 ± 2.34 ppb of Ga/ million cells for **4**. Fractionation studies were conducted with **2** as a representative member of the series (Figure S51, Supporting Information). A large proportion of internalized **2** was detected in the cytoplasm (70%) suggesting that **2**‐mediated osteosarcoma cell toxicity is likely to be related to interactions with cytoplasmic biomolecules. Nevertheless, a relatively small but appreciable amount of internalized **2** was also detected in the nucleus (4%) indicating that a genomic DNA damage related mechanism of action for **2** could not be completely ruled out.

### 2.7 Mode of Cell Death

**Figure 4:**
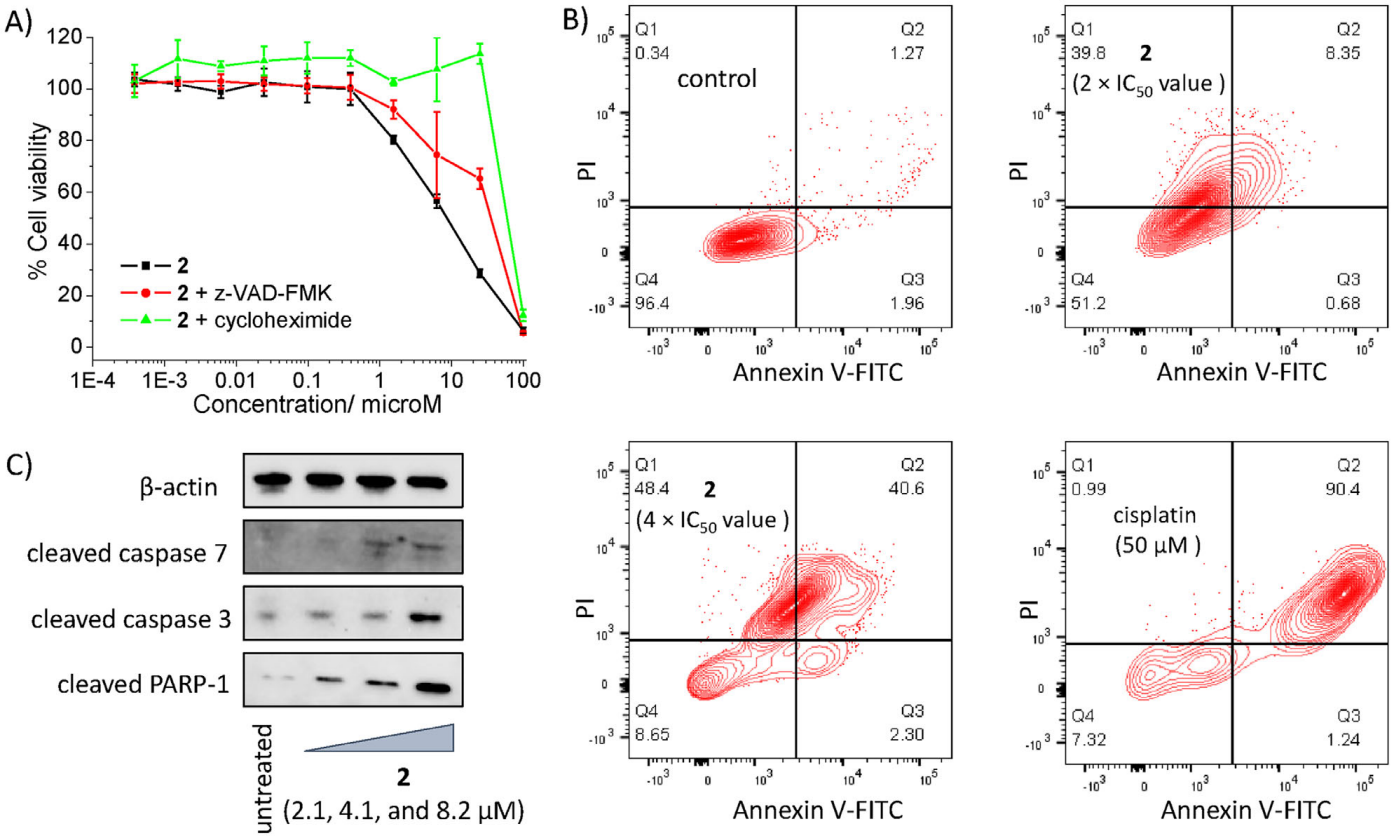
A) Representative dose‐response curves for the treatment of U2OS cells with **2** in the absence and presence of z‐VAD‐FMK (5 µm) or cycloheximide (1 µm) after 72 h incubation. B) FITC Annexin V‐propidium iodide binding assay plots of untreated U2OS cells and U2OS cells treated with **2** (2 × IC_50_ for 72 h) or **2** (4 × IC_50_ for 72 h) or cisplatin (50 µm for 72 h). C) Immunoblotting analysis of proteins involved in the apoptosis cell death pathway. Protein expression in U2OS cells following treatment with **2** (2.1–8.2 µm) for 72 h.

To shed light on the mode of osteosarcoma cell death induced by **2**, cytotoxicity studies were performed in the presence of inhibitors for distinct cell death pathways (Figure 4A; Figure S52 and Table S8, Supporting Information). Specifically, apoptosis (z‐VAD‐FMK, 5 µm),^[^ ^25^ ^]^ necroptosis (necrostatin‐1, 20 µm),^[^ ^26^ ^]^ ferroptosis (ferrostatin‐1, 10 µm),^[^ ^27^ ^]^ autophagy (chloroquine, 10 µm),^[^ ^28^ ^]^ and paraptosis (cycloheximide, 1 µm)^[^ ^29^ ^]^ inhibitors were used. The IC_50_ value of **2** toward U2OS cells did not change significantly when co‐incubated with necrostatin‐1, ferrostatin‐1 or chloroquine implying that **2** is unlikely to induce osteosarcoma cell death by necroptosis, ferroptosis or autophagy. The IC_50_ value of **2** toward U2OS cells increased significantly (*p* < 0.05, *n* = 18) in the presence of z‐VAD‐FMK and cycloheximide indicating that **2** is likely to induce apoptosis and paraptosis in osteosarcoma cells.

Apoptosis can result in the reorganization of the cell membrane, whereby phosphatidylserine residues are translocated from the interior to the exterior.^[^ ^30^ ^]^ The exposure of phosphatidylserine residues on the cell membrane exterior can be detected by Annexin V.^[^ ^31^ ^]^ Apoptosis can also result in widespread cell membrane damage, which enables cell impermeable agents such as propidium iodide to penetrate the cell membrane.^[^ ^31^ ^]^ The dual FITC Annexin V‐propidium iodide staining flow cytometry assay was used to determine if **2** induced morphological changes to osteosarcoma cells were consistent with apoptosis (Figure 4B). Incubation of U2OS cells with **2** (2 × IC_50_ value or 4 × IC_50_ value for 72 h) led to a significant increase in the population of cells expressing late‐stage apoptotic‐like features (Figure 4B). A marked increase in the population of cells displaying late‐stage apoptotic features was also observed upon treatment with cisplatin (50 µm for 72 h), an established apoptosis inducer (Figure 4B). Independent immunoblotting studies showed that U2OS cells treated with **2** (2.1–8.2 µm for 72 h) displayed higher levels of cleaved caspase 3 and 7, and poly‐ADP ribose polymerase (PARP) compared to untreated U2OS cells (Figure 4C), indicative of caspase‐dependent apoptosis.

**Figure 5:**
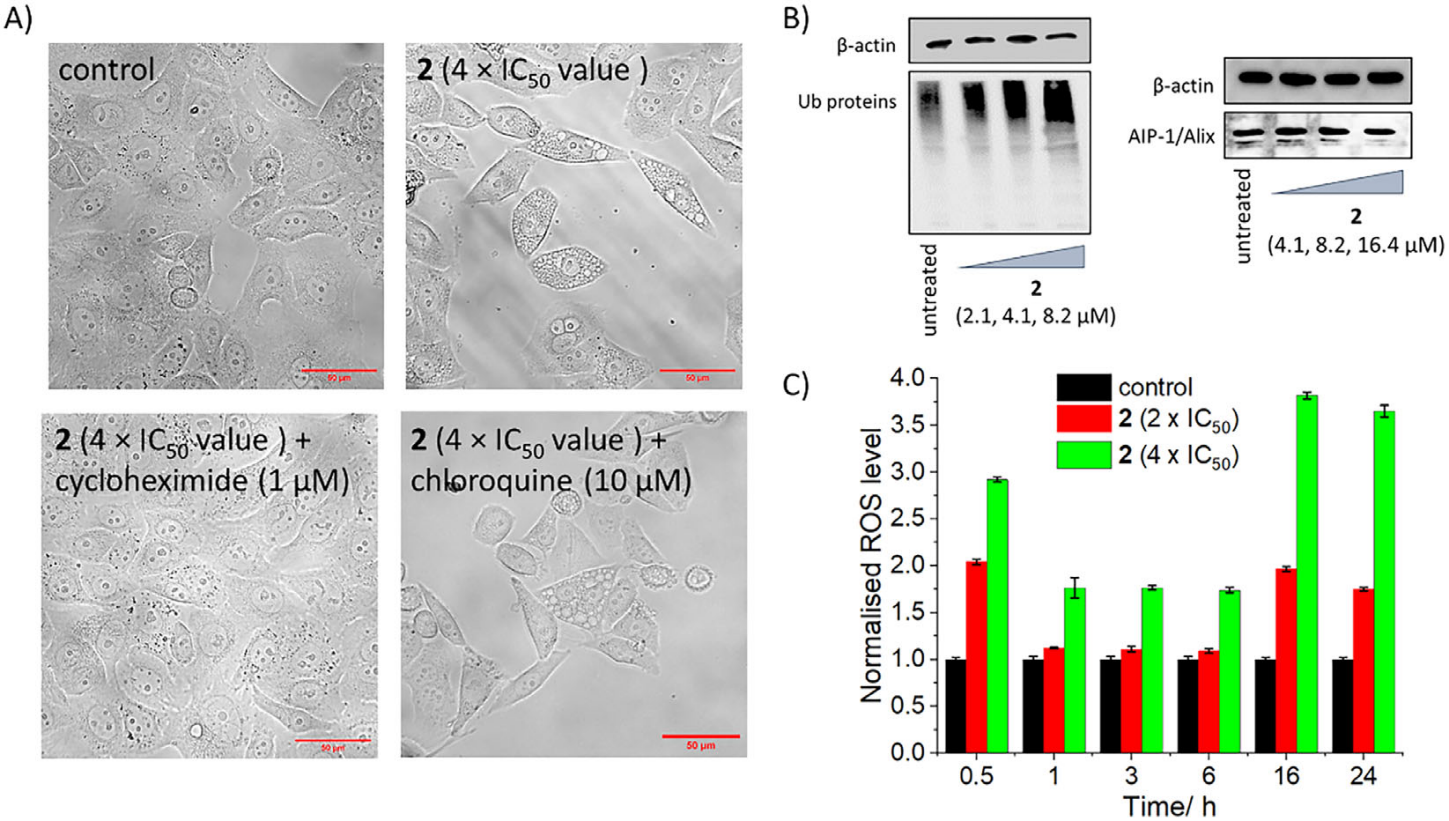
A) Representative bright‐field images (× 60) of untreated U2OS cells, U2OS cells treated with **2** (4 × IC_50_ value for 24 h), U2OS cells co‐treated with **2** (4 × IC_50_ value for 24 h) and cycloheximide (1 µm for 24 h), and U2OS cells co‐treated with **2** (4 × IC_50_ value for 24 h) and chloroquine (10 µm for 24 h). B) Immunoblotting analysis of proteins related to the paraptosis pathway. Protein expression in U2OS cells following treatment with **2** (2.1–8.2 µm after 72 h incubation or 4.1–16.4 µm after 4 h incubation). C) Normalized ROS activity in untreated U2OS cells and U2OS cells treated with **2** (2 × IC_50_ value or 4 × IC_50_ value for 0.5, 1, 3, 6, 16, and 24 h). Error bars represent standard deviations.

The morphology of osteosarcoma cells treated with **2** was investigated using an inverted microscope. Upon incubation of U2OS cells with **2** (4 × IC_50_ value for 24 h), a clear increase in intracellular vacuolization was observed (Figure 5A). Intracellular vacuolization was significantly attenuated when **2** (4 × IC_50_ value for 24 h) was co‐treated with cycloheximide (1 µm) but not chloroquine (10 µm) (Figure 5A). Given that cycloheximide is a protein synthesis inhibitor that halts paraptosis^[^ ^29^, ^32^ ^]^ and chloroquine inhibits autophagic flux and blocks autophagosome fusion,^[^ ^28^ ^]^ the morphology studies suggest that **2**‐induced vacuole formation could be a manifestation of paraptosis and not autophagy.^[^ ^33^ ^]^ The inhibition of proteasomal activity and the subsequent accumulation of ubiquitinated proteins is a distinctive feature associated to paraptosis.^[^ ^33^ ^]^ Independent immunoblotting studies showed that U2OS cells treated with **2** (2.1–8.2 µm for 72 h) displayed noticeably higher levels of poly‐ubiquitinated proteins than untreated U2OS cells (Figure 5B), implicative of paraptosis. AIP‐1/Alix is a phylogenetically conserved cytosolic scaffold protein that inhibits paraptosis.^[^ ^34^ ^]^ Immunoblotting studies indicated a noticeable decrease in AIP‐1/Alix expression in U2OS cells treated with **2** (4.1–16.4 µm for 4 h) (Figure 5B), providing further evidence of **2**‐induced paraptosis. During paraptosis, perturbations in intracellular calcium transport are common.^[^ ^35^ ^]^ Paraptosis‐inducing agents trigger calcium release from the ER to the cytoplasm.^[^ ^35^ ^]^ Relative cytoplasmic calcium levels were monitored using the calcium‐specific Fluo‐4 AM dye. U2OS cells treated with **2 (**2 × IC_50_ value) for 24 h displayed a significant increase (46%) in cytoplasmic calcium levels compared to untreated cells (Figure S53, Supporting Information). The elevation in **2**‐induced cytoplasmic calcium levels was significantly attenuated (*p* < 0.05, 36% reduction) in the presence of cycloheximide (1 µm) (Figure S53, Supporting Information), suggesting that the perturbation in calcium homeostasis induced **2** is related to its ability to evoke paraptosis. A common downstream characteristic for paraptosis‐inducing agents is the increase in intracellular reactive oxygen species (ROS) levels.^[^ ^36^ ^]^ Perturbations in intracellular ROS levels upon incubation of U2OS cells with **2** (2 × IC_50_ value or 4 × IC_50_ value) were measured using the well‐established ROS detector, 6‐carboxy‐2′,7′‐dichlorodihydrofluorescein diacetate (DCFH‐DA), over the course of 24 h (Figure 5C). U2OS cells treated with **2** displayed a significant (*p* < 0.05) increase in intracellular ROS levels, at short (0.5 h) and prolonged (16–24 h) incubation times (up to 3.8‐fold). This suggests that **2** has the potential to induce ROS‐mediated paraptosis. Overall, the mechanistic investigations suggest that **2** can induce hallmarks of both apoptosis and paraptosis in osteosarcoma cells.

### 2.8 Immunogenic Cell Death and Phagocytosis

**Figure 6:**
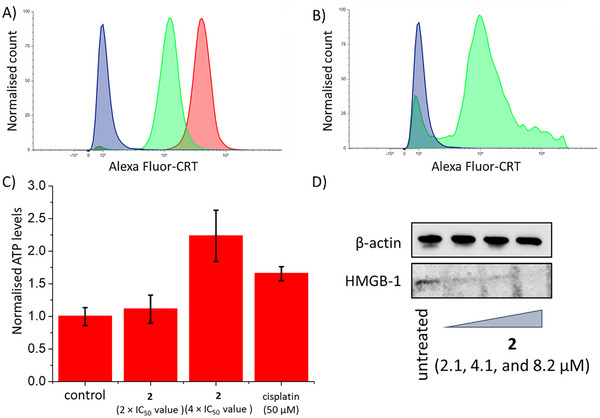
A,B) Representative histograms displaying the green fluorescence emitted by anti‐CRT Alexa Fluor 488 nm antibody‐stained U2OS cells (A) untreated (blue), and treated with **2** (2 × IC_50_ value) (green) or **2** (4 × IC_50_ value) (red) for 24 h and (B) U2OS cells untreated (blue) and treated with cisplatin (150 µm) and thapsigargin (7 µm) (green) for 24 h. C) Normalized extracellular ATP released from U2OS cells untreated and treated with **2** (2 × IC_50_ value and 4 × IC_50_ value) or cisplatin (50 µm) for 24 h. D) Immunoblotting analysis of high mobility group box 1 (HMGB‐1). Protein expression in U2OS cells following treatment with **2** (2.1–8.2 µm) for 72 h.

Both apoptosis and paraptosis can result in the release or exposure of danger‐associated molecular patterns (DAMPs), ATP, calreticulin, and high‐mobility group box 1, which are biomarkers of immunogenic cell death (ICD).^[^ ^36^, ^37^ ^]^ Given that **2** is able to evoke apoptosis and paraptosis of osteosarcoma cells, we investigated whether it could also induce DAMP release or exposure. U2OS cells treated with **2** (2 × IC_50_ value or 4 × IC_50_ value for 24 h) displayed markedly higher levels of CRT on their cell membrane than untreated control cells (Figure 6A). U2OS cells treated with cisplatin (150 µm for 24 h) and thapsigargin (7 µm for 24 h), a combination known to induce ICD,^[^ ^38^ ^]^ also displayed a clear increase in CRT on their cell membrane (Figure 6B). A luciferase‐based assay was used to determine the release of ATP from U2OS cells treated with **2**. U2OS cells treated with **2** (2 × IC_50_ value or 4 × IC_50_ value for 24 h) released ATP in a dose‐dependent manner (up to 2.2‐fold more than untreated cells) (Figure 6C). As expected, cisplatin (50 µm for 24 h) also induced significant ATP release (1.7‐fold more than untreated cells) (Figure 6C). Immunoblotting studies showed that U2OS cells treated with **2** (2.1–8.2 µm for 72 h) displayed markedly lower or undetectable amounts of HMGB‐1 relative to untreated control cells, indicative of HMGB‐1 expulsion (Figure 6D). Overall, the DAMP detection studies suggest that **2** is capable of inducing ICD of osteosarcoma.

**Figure 7:**
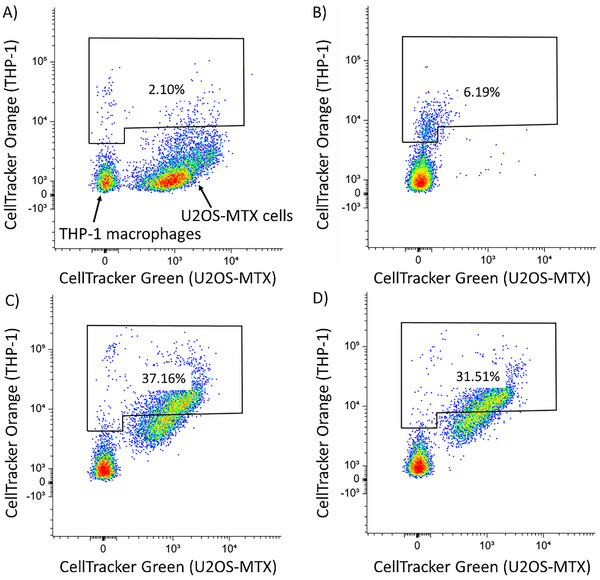
A–D) Representative 2D scatter plots of CellTracker Green‐stained U2OS‐MTX cells (A) untreated and treated with (B) cisplatin (150 µm) and thapsigargin (7 µm) or (C) **2** (25 µm) or (D) **2** (50 µm) for 24 h and then co‐cultured with CellTracker Orange‐stained THP‐1 macrophages for 2 h.

After ICD, dead cancer cells need to be phagocytosed and processed before a targeted immune response can be activated. The ability of U2OS and U2OS‐MTX cells treated with **2** to be phagocytosed by macrophages was determined using an in vitro phagocytosis assay. U2OS or U2OS‐MTX cells pre‐stained with CellTracker Green were treated with **2** (25–50 µm for 24 h) and then incubated with macrophages pre‐stained with CellTracker Orange for 2 h. The population of double‐stained macrophages and engulfed U2OS or U2OS‐MTX cells is indicated in the 2D scatter plots shown in Figure 7 and Figure S54 (Supporting Information). The phagocytosis assay showed that **2** (25–50 µm for 24 h) was able to significantly enhance engulfment of U2OS and U2OS‐MTX cells by macrophages (up to 98‐fold increase compared to untreated U2OS cells, Figure S54, Supporting Information and up to 18‐fold increase compared to untreated U2OS‐MTX cells, Figure 7). The addition of cisplatin (150 µm for 24 h) and thapsigargin (7 µm for 24 h) to U2OS or U2OS‐MTX cells also resulted in a significant increase in the population of double‐stained macrophages (3–24‐fold increase compared to untreated cells, Figure 7; Figure S54, Supporting Information) indicative of phagocytosis. Overall, these results show that bi‐nuclear gallium(III) complex **2** is able to kill osteosarcoma cells and OSCs in a manner that promotes engulfment by macrophages.

## 3 Conclusion

In summary, we report the synthesis, characterization, anti‐osteosarcoma and anti‐OSC activity of a series of bi‐nuclear gallium(III) complexes **1**–**4** containing two tridentate Schiff base ligands (with various halogen substituents) and two 8‐hydroxyquinoline moieties. X‐ray crystallography studies revealed that the structure of the bi‐nuclear gallium(III) complexes **1** and **2** comprise of two distorted octahedral gallium(III) centres with the Schiff base ligands and 8‐hydroxyquinoline occupying both terminal and bridging positions. The bi‐nuclear gallium(III) complexes **1**–**4** displayed micromolar potency toward bulk osteosarcoma and OSCs cultured in monolayer systems. The most active bi‐nuclear gallium(III) complex **2** was up to fourfold and 13‐fold more potent toward OSCs than cisplatin and carboplatin, respectively. The bi‐nuclear gallium(III) complexes **1**–**4** were able to reduce the viability of sarcospheres (cultured in 3D conditions) in the low micromolar or sub‐micromolar range. Based on the IC_50_ values, **1**–**4** were up to 25‐fold more potent toward sarcospheres than monolayer cultured OSCs. This is atypical for small molecule anticancer agents (as they tend to display the opposite trend) and suggests that **1**–**4** have the capacity to penetrate the multicellular nature of sarcospheres. This is also a highly attractive feature with respect to translation. Remarkably, **1**–**4** were up to 53‐fold more active toward sarcospheres than cisplatin, carboplatin, and salinomycin. Detailed mechanistic studies indicated that osteosarcoma cells killed by the most active bi‐nuclear gallium(III) complex **2** displayed both apoptosis and paraptosis characteristics. Specifically, osteosarcoma cells treated with **2** displayed phosphatidylserine residues on their cell membrane exterior and an increase in cleaved caspase 3 and 7 and PARP‐1 levels, implicative of caspase‐dependent apoptosis. Osteosarcoma cells treated with **2** also exhibited specific hallmarks associated with paraptosis such as intracellular vacuolizsation, an increase in ubiquitinated proteins, a decrease in AIP‐1/Alix expression, and significant increases in intracellular calcium and ROS levels. Osteosarcoma cells dosed with **2** exhibited DAMPs consistent with ICD, such as CRT exposure on their cell membrane exterior and extracellular release of ATP and HMGB‐1. Phagocytosis studies showed that osteosarcoma cells and OSCs dosed with **2** were effectively engulfed by macrophages, highlighting the promising immunogenic potential of **2**. To the best of our knowledge, **2** is the first metal complex to display both cytotoxic and immunogenic effects toward bulk osteosarcoma cells and OSCs. Not only does this study reinforce the therapeutic potential of gallium(III) complexes but it also provides the basis for the development of other metal complexes as anti‐osteosarcoma drug candidates. Furthermore, the results show that metal complexes that can induce paraptosis can be effective immunotherapeutic agents toward chemotherpy resistant osteosarcoma sub‐populations such as OSCs.

## Supporting Information

The authors have cited additional references within the Supporting Information.^[^ ^39^ ^]^

## Conflict of Interests

The authors declare no conflict of interest.

## Supporting information



Supporting Information

## Data Availability

The data that support the findings of this study are available from the corresponding author upon reasonable request.
