# Supplementary material for: Osteosarcoma Cell and Osteosarcoma Stem Cell Potent Immunogenic Bi‐Nuclear Gallium(III) Complexes
Source: Chemistry. 2025 Apr 23;31(29):e202500747. doi: 10.1002/chem.202500747 (PMC12099189; doi:10.1002/chem.202500747)
Supplement: Supplementary file 1 — Supporting Information [file CHEM-31-e202500747-s001.pdf]

Supporting Information for

Osteosarcoma Cell and Osteosarcoma Stem Cell Potent Immunogenic Bi-nuclear Gallium(III) Complexes

Xiao Feng,[a] Shruti Dhandore,[a] Yu Liu,[a] Kuldip Singh,[a] Fabrizio Ortu,*[a] and Kogularamanan Suntharalingam*[a]

[a] School of Chemistry, University of Leicester, Leicester, UK

* To whom correspondence should be addressed:
Email: k.suntharalingam@leicester.ac.uk; fabrizio.ortu@leicester.ac.uk

Table of Content

Experimental Details
Figure S1. ^{1}H NMR spectrum (400 MHz, DMSO-d_6) of **L¹**.
Figure S2. ^{19}F{^{1}H} NMR spectrum (376 MHz, DMSO-d_6) of **L¹**.
Figure S3. ^{1}H NMR spectrum (400 MHz, DMSO-d_6) of **L²**.
Figure S4. ^{1}H NMR spectrum (400 MHz, DMSO-d_6) of **L³**.
Figure S5. ^{1}H NMR spectrum (400 MHz, DMSO-d_6) of **L⁴**.
Figure S6. ^{1}H NMR spectra (400 MHz, DMSO-d_6) of 5-fluorosalicylaldehyde, 5-chlorosalicylaldehyde, 5-bromosalicylaldehyde, and 5-iodosalicylaldehyde.
Figure S7. ATR-FTIR spectra of (A) **L¹**, (B) **L²**, (C) **L³**, and (D) **L⁴** in the solid form.
Figure S8. High resolution ESI mass spectrum (positive mode) of **L¹**.
Figure S9. High resolution ESI mass spectrum (positive mode) of **L²**.
Figure S10. High resolution ESI mass spectrum (positive mode) of **L³**.
Figure S11. High resolution ESI mass spectrum (positive mode) of **L⁴**.
Figure S12. ^{1}H NMR spectrum (400 MHz, DMSO-d_6) of **1**.
Figure S13. ^{19}F{^{1}H} NMR spectrum (376 MHz, DMSO-d_6) of **1**.
Figure S14. ^{1}H NMR spectrum (400 MHz, DMSO-d_6) of **2**.
Figure S15. ^{1}H NMR spectrum (400 MHz, DMSO-d_6) of **3**.
Figure S16. ^{1}H NMR spectrum (400 MHz, DMSO-d_6) of **4**.
Figure S17. ATR-FTIR spectra of (A) **1**, (B) **2**, (C) **3**, and (D) **4** in the solid form.
Figure S18. (Top) Theoretical isotope model for [**1**+H]$^+$ ($C_{44}H_{29}Ga_2N_4O_6F_2$) and (bottom) the experimentally determined high-resolution ESI-TOF mass spectrum for complex **1**.
Figure S19. (Top and middle) Theoretical isotope model for [**2**+Na]$^+$ ($C_{44}H_{28}Ga_2N_4O_6Cl_2Na$) and [**2**+H]$^+$ ($C_{44}H_{29}Ga_2N_4O_6Cl_2$) and (bottom) the experimentally determined high-resolution ESI-TOF mass spectrum for complex **2**.

Figure S20. (Top) Theoretical isotope model for [**3**+H]$^+$ ($C_{44}H_{29}Ga_2N_4O_6Br_2$) and (bottom) the experimentally determined high-resolution ESI-TOF mass spectrum for complex **3**.
Figure S21. (Top) Theoretical isotope model for [**4**+H]$^+$ ($C_{44}H_{29}Ga_2N_4O_6I_2$) and (bottom) the experimentally determined high-resolution ESI-TOF mass spectrum for complex **4**.
Table S1. Crystallographic data for complexes **1** and **2**.
Table S2. Selected bond lengths (Å) and angles (°) for complex **1**.
Figure S22. X-ray structure of **2**. Ellipsoids are shown at 50% probability, H atoms have been omitted for clarity. C in black, N in dark blue, O in red, Cl in green, and Ga in grey.
Table S3. Experimentally determined LogP values for **1**-**4**.
Figure S23. UV-vis spectra of (A) **1**, (B) **2**, (C) **3**, and (D) **4** (all 50 µM) in DMSO over the course of 24 h at 37 ^{o}C.
Figure S24. ^{1}H NMR spectra of **1** (10 mM) in DMSO-d_6 over the course of 72 h.
Figure S25. ^{1}H NMR spectra of **2** (10 mM) in DMSO-d_6 over the course of 72 h.
Figure S26. ^{1}H NMR spectra of **3** (10 mM) in DMSO-d_6 over the course of 72 h.
Figure S27. ^{1}H NMR spectra of **4** (10 mM) in DMSO-d_6 over the course of 72 h.
Figure S28. ESI mass spectra (positive mode) of **1** (0.5 mM) in DMSO after incubation for (A) 0 h or (B) 24 h at 37 ^{o}C.
Figure S29. ESI mass spectra (positive mode) of **2** (0.5 mM) in DMSO after incubation for (A) 0 h or (B) 24 h at 37 ^{o}C.
Figure S30. ESI mass spectra (positive mode) of **3** (0.5 mM) in DMSO after incubation for (A) 0 h or (B) 24 h at 37 ^{o}C.
Figure S31. ESI mass spectra (positive mode) of **4** (0.5 mM) in DMSO after incubation for (A) 0 h or (B) 24 h at 37 ^{o}C.
Figure S32. UV-vis spectra of (A) **1**, (B) **2**, (C) **3**, and (D) **4** (all 50 µM) in H_2O:DMSO (200:1**)** over the course of 24 h at 37 ^{o}C.
Table S4. Crystallographic data for complexes **1a** and **3a**.
Table S5. Selected bond lengths (Å) and angles (°) for complex **1a**.
Table S6. Selected bond lengths (Å) and angles (°) for complex **3a**.
Figure S33. Chemical structures of the mono-nuclear gallium(III)-DMSO complexes **1a**-**4a**. The mono-nuclear gallium(III)-DMSO complexes **1a**-**4a** are observed in small amounts in H_2O:DMSO solutions.
Figure S34. ^{1}H NMR spectra of **1** (10 mM) in D_2O:DMSO-d_6 (9:1) over the course of 72 h. (A) aromatic region and (B) aliphatic region.
Figure S35. ^{1}H NMR spectra of **2** (10 mM) in D_2O:DMSO-d_6 (9:1) over the course of 72 h. (A) aromatic region and (B) aliphatic region.
Figure S36. ^{1}H NMR spectra of **3** (10 mM) in D_2O:DMSO-d_6 (9:1) over the course of 72 h. (A) aromatic region and (B) aliphatic region.
Figure S37. ^{1}H NMR spectra of **4** (10 mM) in D_2O:DMSO-d_6 (9:1) over the course of 72 h. (A) aromatic region and (B) aliphatic region.
Figure S38. UV-vis spectra of (A) **1**, (B) **2**, (C) **3**, and (D) **4** (all 50 µM) in MEGM:DMSO (200:1**)** over the course of 24 h at 37 ^{o}C.
Figure S39. Solution depletion plots showing the relative amount of **1**-**4** remaining in solution (unbound to hydroxyapatite) over the course of 48 h.
Figure S40. Representative dose-response curves for the treatment of U2OS and U2OS-MTX cells with **1** after 72 h incubation.
Figure S41. Representative dose-response curves for the treatment of U2OS and U2OS-MTX cells with **2** after 72 h incubation.

Figure S42. Representative dose-response curves for the treatment of U2OS and U2OS-MTX cells with **3** after 72 h incubation.

Figure S43. Representative dose-response curves for the treatment of U2OS and U2OS-MTX cells with **4** after 72 h incubation.

Figure S44. Representative dose-response curves for the treatment of U2OS and U2OS-MTX cells with $\mathbf{L^1}$ after 72 h incubation.

Figure S45. Representative dose-response curves for the treatment of U2OS and U2OS-MTX cells with $\mathbf{L^2}$ after 72 h incubation.

Figure S46. Representative dose-response curves for the treatment of U2OS and U2OS-MTX cells with $\mathbf{L^3}$ after 72 h incubation.

Figure S47. Representative dose-response curves for the treatment of U2OS and U2OS-MTX cells with $\mathbf{L^4}$ after 72 h incubation.

Table S7. IC_{50} values of $\mathbf{L^1}$-$\mathbf{L^4}$ against U2OS and U2OS-MTX cells. [a] Determined after 72 h incubation (mean of three independent experiments ± SD).

Figure S48. Representative bright-field images (× 10) of U2OS-MTX sarcospheres in the absence and presence of salinomycin at its IC_{20} values for 10 days.

Figure S49. Representative dose-response curves for the treatment of U2OS-MTX sarcospheres with **1-4** after 10 days incubation.

Figure S50. The amount of gallium (in terms of ng of Ga/ million cells) present in U2OS cells treated with **1-4** (5 μM for 24 h).

Figure S51. Gallium content (ng of Ga/ 10^6 cells) in various cellular components upon treatment of U2OS cells with **2** (5 μM for 24 h).

Figure S52. Representative dose-response curves for the treatment of U2OS cells with **2** in the presence of z-VAD-FMK (5 μM), necrostatin-1 (20 μM), ferrostatin-1 (10 μM), chloroquine (10 μM) or cycloheximide (1 μM) after 72 h incubation.

Table S8. IC_{50} values of **2** against U2OS in the absence and presence of apoptosis (z-VAD-FMK, 5 μM), necroptosis (necrostatin-1, 20 μM), ferroptosis (ferrostatin-1, 10 μM), autophagy (chloroquine, 10 μM), and paraptosis (cycloheximide, 1 μM) inhibitors.

Figure S53. Normalised cytoplasmic calcium levels in U2OS cells untreated and treated with **2** (2 × IC_{50} value for 24 h) or co-treated with **2** (2 × IC_{50} value for 24 h) and cycloheximide (1 μM for 24 h).

Figure S54. Representative two-dimensional scatter plots of CellTracker Green-stained U2OS cells (A) untreated and treated with (B) cisplatin (150 μM) and thapsigargin (7 μM) or (C) **2** (25 μM) or (D) **2** (50 μM) for 24 h and then co-cultured with CellTracker Orange-stained THP-1 macrophages for 2 h.

References

Experimental Details

Materials and Methods. All synthetic procedures were performed under normal atmospheric conditions or under nitrogen. Fourier transform infrared (FTIR) spectra were recorded with an IRAffinity-1S Shimadzu spectrophotometer. Electron spray ionisation mass spectra were recorded on a Micromass Quattro spectrometer. UV-vis absorption spectra were recorded on a Cary 3500 UV-Vis spectrophotometer. ^{1}H and ^{19}F{^{1}H} NMR spectra were recorded on a BrukerAvance 400 MHz Ultrashield NMR spectrometer. ^{1}H NMR spectra were referenced internally to residual solvent peaks, and chemical shifts are expressed relative to tetramethylsilane, $SiMe_4$ (δ = 0 ppm). Elemental analysis of the compounds prepared was performed commercially by the University of Cambridge. $GaCl_3$, 2-aminophenol, 5-fluorosalicylaldehyde, 5-chlorosalicylaldehyde, 5-bromosalicylaldehyde, 5-iodosalicylaldehyde, 8-hydroxyquinoline, and piperidine were purchased from Sigma Aldrich or Alfa Aesar and used as received.

Synthesis of (4-fluoro-)2-[[(2-hydroxyphenyl)imino]methyl]phenol, L^{1}. A mixture of 2-aminophenol (109.1 mg, 1 mmol) and 5-fluorosalicylaldehyde (140.1 mg, 1 mmol) was refluxed in methanol (15 mL) for 2 h. The reaction mixture was cooled to room temperature and poured into cold water (30 mL), resulting in a precipitate. The precipitate was filtered, washed with cold water (10 mL) and cold methanol (3 mL), and dried under vacuum overnight, yielding **L^{1}** as a yellow solid (178.4 mg, 77.2%); ^{1}H NMR (400 MHz, DMSO-d_6): δ_H 13.41 (s, 1H), 9.75 (s, 1H), 8.96 (s, 1H), 7.52 (d, 1H), 7.34 (d, 1H), 7.25 (t, 1H), 7.15 (t, 1H), 6.96 (d, 2H), 6.89 (t, 1H); ^{19}F NMR (376 MHz, DMSO-d_6): δ_F −125.59 (1F, s); IR (solid, ATR, cm^{-1}): 3050, 1632, 1533, 1458, 1304, 1270, 1233, 1133, 821, 789, 746, 558, 483, 430, 377; HR ESI-MS: Calcd. for $C_{13}H_{11}NO_2F$ $[M+H]^+$ 232.2142 a.m.u. Found $[M+H]^+$ 232.0776 a.m.u.

Synthesis of (4-chloro-)2-[[(2-hydroxyphenyl)imino]methyl]phenol, L^{2}. A mixture of 2-aminophenol (109.1 mg, 1 mmol) and 5-chlorosalicylaldehyde (156.6 mg, 1 mmol) was refluxed in methanol (15 mL) for 2 h. The reaction mixture was cooled to room temperature and poured into cold water (30 mL), resulting in a precipitate. The precipitate was filtered, washed with cold water (10 mL) and cold methanol (3 mL), and dried under vacuum overnight, yielding **L^{2}** as an orange solid (174 mg, 70.3%); ^{1}H NMR (400 MHz, DMSO-d_{6},): δ_H 13.77 (s, 1H), 9.79 (s, 1H), 8.98 (s, 1H), 7.74 (d, 1H), 7.41 (dd, 1H), 7.36 (d, 1H), 7.15 (t, 1H), 6.97 (d, 2H), 6.89 (t, 1H); IR (solid, ATR, cm^{-1}): 3071, 1625, 1508, 1455, 1377, 1268, 1215, 1142, 1128, 1112, 1030, 828, 755, 689, 517, 483, 400; HR ESI-MS: Calcd. for $C_{13}H_{11}NO_2Cl$ $[M+H]^+$ 248.6618 a.m.u. Found $[M+H]^+$ 248.0486 a.m.u.

Synthesis of (4-bromo-)2-[[(2-hydroxyphenyl)imino]methyl]phenol, L^{3}. A mixture of 2-aminophenol (109.1 mg, 1 mmol) and 5-bromosalicylaldehyde (201.2 mg, 1 mmol) was refluxed in methanol (15 mL) for 2 h. The reaction mixture was cooled to room temperature and poured into cold water (30 mL), resulting in a precipitate. The precipitate was filtered, washed with cold water (10 mL) and cold methanol (3 mL), and dried under vacuum overnight, yielding **L^{3}** as an orange solid (128.7 mg, 44.1%); ^{1}H NMR (400 MHz, DMSO-d_6): δ_H 13.81 (s, 1H), 9.80 (s, 1H), 8.97 (s, 1H), 7.86 (s, 1H), 7.52 (d, 1H), 7.35 (d, 1H), 7.15 (t, 1H), 6.97 (d, 1H), 6.90 (dd, 2H); IR (solid, ATR, cm^{-1}): 3508, 3069, 1627, 1508, 1453, 1380, 1268, 1220, 1128, 1112, 1025, 879, 824, 753, 622, 515, 483, 398; HR ESI-MS: Calcd. for $C_{13}H_{11}NO_2Br$ $[M+H]^+$ 293.1198 a.m.u. Found $[M+H]^+$ 293.9963 a.m.u.

Synthesis of (4-iodo-)2-[[(2-hydroxyphenyl)imino]methyl]phenol, L^{4}. A mixture of 2-aminophenol (109.1 mg, 1 mmol) and 5-iodosalicylaldehyde (248.0 mg, 1 mmol) was refluxed

in methanol (15 mL) for 2 h. The reaction mixture was cooled to room temperature and poured into cold water (30 mL), resulting in precipitate. The precipitate was filtered, washed with cold water (10 mL) and cold methanol (3 mL), and dried under vacuum overnight, yielding $\mathbf{L^4}$ as an orange solid (220.8 mg, 65.1%); ^{1}H NMR (400 MHz, DMSO-d_6): δ_H 13.82 (s, 1H), 9.85 (s, 1H), 8.95 (s, 1H), 7.98 (s, 1H), 7.64 (d, 1H), 7.35 (d, 1H), 7.14 (t, 1H), 6.97 (d, 1H), 6.89 (t, 1H), 6.79 (d, 1H); IR (solid, ATR, cm^{-1}): 3066, 1620, 1588, 1503, 1373, 1210, 1107, 1018, 920, 821, 737, 661, 609, 517, 480, 393; HR ESI-MS: Calcd. for $C_{13}H_{11}NO_2I$ [M+H]$^+$ 340.1202 a.m.u. Found [M+H]$^+$ 339.9839 a.m.u.

Synthesis of $Ga_2(L^1)_2$(8-hydroxyquinoline)$_2$, 1. A mixture of $\mathbf{L^1}$ (100.0 mg, 0.4 mmol) and piperidine (85.6 µL, 73.7 mg, 0.9 mmol) in ethanol (10 mL) was slowly added to $GaCl_3$ (76.2 mg, 0.4 mmol) in ethanol (2 mL). The reaction was stirred under nitrogen for 0.5 h, then to this solution was added a mixture of 8-hydroxyquinoline (62.9 mg, 0.4 mmol) and piperidine (42.8 µL, 36.9 mg, 0.4 mmol) in ethanol (2 mL). The resultant mixture was stirred for 2 h. During this period a precipitate formed. The precipitate was collected by filtration, washed with cold ethanol (15 mL) and water (30 mL), and dried under vacuum to yield **1** as an orange powder (81.5 mg, 42.5%); ^{1}H NMR (400 MHz, DMSO-d_6): δ_H 9.23 (s, 2H), 8.47 (d, 2H), 8.21 (s, 2H), 7.75 (dd, 2H), 7.51 (t, 4H), 7.35 (dd, 2H), 7.12 (d, 2H), 7.09 – 6.99 (m, 4H), 6.86 (d, 2H), 6.62 – 6.58 (m, 2H), 6.49 – 6.45 (m, 4H); ^{19}F NMR (376 MHz, DMSO-d_6): δ_F −130.38 (2F, s); IR (solid, ATR, cm^{-1}): 1613, 1540, 1463, 1378, 1309, 1269, 1203, 1142, 1109, 819, 742, 625, 523, 404; HR ESI-MS: Calcd. for $C_{44}H_{29}Ga_2N_4O_6F_2$ [M+H]$^+$ 887.0565 a.m.u. Found [M+H]$^+$ 887.0568 a.m.u.; Anal. Calcd. for $C_{44}H_{28}Ga_2N_4O_6F_2\cdot H_2O$ (%): C 58.45; H 3.34; N 6.20. Found: C 58.18; H 3.27; N 6.07.

Synthesis of $Ga_2(L^2)_2$(8-hydroxyquinoline)$_2$, 2. A mixture of $\mathbf{L^2}$ (100.0 mg, 0.4 mmol) and piperidine (79.7 µL, 68.8 mg, 0.8 mmol) in ethanol (10 mL) was slowly added to $GaCl_3$ (71.1 mg, 0.4 mmol) in ethanol (2 mL). The reaction was stirred under nitrogen for 0.5 h, then to this solution was added a mixture of 8-hydroxyquinoline (58.6 mg, 0.4 mmol) and piperidine (39.9 µL, 34.4 mg, 0.4 mmol) in ethanol (2 mL). The resultant mixture was stirred for 2 h. During this period a precipitate formed. The precipitate was collected by filtration, washed with cold ethanol (15 mL) and water (30 mL), and dried under vacuum to yield **2** as an orange powder (107.0 mg, 57.7%); ^{1}H NMR (400 MHz, DMSO-d_6): δ_H 9.24 (s, 2H), 8.47 (d, 2H), 8.19 (s, 2H), 7.76 (d, 2H), 7.61 (d, 2H), 7.51 (t, 4H), 7.18 – 7.12 (m, 4H), 7.02 (t, 2H), 6.87 (d, 2H), 6.61 (t, 2H), 6.49 (d, 4H); IR (solid, ATR, cm^{-1}): 1615, 1494, 1463, 1374, 1303, 1265, 1165, 1113, 1036, 823, 738, 696, 523, 408; HR ESI-MS: Calcd. for $C_{44}H_{29}Ga_2N_4O_6Cl_2$ [M+H]$^+$ 918.9965 a.m.u. Found [M+H]$^+$ 918.9961 a.m.u.; Anal. Calcd. for $C_{44}H_{28}Ga_2N_4O_6Cl_2\cdot 1.5H_2O$ (%): C 55.86; H 3.30; N 5.92. Found: C 55.96; H 3.42; N 5.73.

Synthesis of $Ga_2(L^3)_2$(8-hydroxyquinoline)$_2$, 3. A mixture of $\mathbf{L^3}$ (100.0 mg, 0.3 mmol) and piperidine (67.6 µL, 58.3 mg, 0.7 mmol) in ethanol (10 mL) was slowly added to $GaCl_3$ (60.3 mg, 0.3 mmol) in ethanol (2 mL). The reaction was stirred under nitrogen for 0.5 h, then to this solution was added a mixture of 8-hydroxyquinoline (49.6 mg, 0.3 mmol) and piperidine (33.8 µL, 29.2 mg, 0.3 mmol) in ethanol (2 mL). The resultant mixture was stirred for 2 h. During this period a precipitate formed. The precipitate was collected by filtration, washed with cold ethanol (15 mL) and water (30 mL), and dried under vacuum to yield **3** as an orange powder (80.0 mg, 46.4%); ^{1}H NMR (400 MHz, DMSO-d_6): δ_H 9.24 (s, 2H), 8.47 (d, 2H), 8.19 (s, 2H), 7.76 – 7.72 (m, 4H), 7.51 (t, 4H), 7.26 (dd, 2H), 7.13 (d, 2H), 7.01 (t, 2H), 6.87 (d, 2H), 6.60 (dd, 2H), 6.48 (d, 2H), 6.44 (d, 2H); IR (solid, ATR, cm^{-1}): 1613, 1462, 1371, 1307, 1265, 1162, 1112, 1037, 819, 739, 677, 647, 538, 524, 407; HR ESI-MS: Calcd. for $C_{44}H_{29}Ga_2N_4O_6$

Br_2 $[M+H]^+$ 1008.8942 a.m.u. Found $[M+H]^+$ 1008.8945 a.m.u.; Anal. Calcd. for $C_{44}H_{28}Ga_2N_4O_6Br_2{\cdot}2H_2O$ (%): C 50.62; H 3.09; N 5.37. Found: C 50.93; H 3.01; N 5.21.

Synthesis of $Ga_2(L^4)_2$(8-hydroxyquinoline)$_2$, 4. A mixture of **L^4** (100.0 mg, 0.3 mmol) and piperidine (58.2 µL, 50.2 mg, 0.6 mmol) in ethanol (10 mL) was slowly added to $GaCl_3$ (51.9 mg, 0.3 mmol) in ethanol (2 mL). The reaction was stirred under nitrogen for 0.5 h, then to this solution was added a mixture of 8-hydroxyquinoline (42.8 mg, 0.3 mmol) and piperidine (29.1 µL, 25.1 mg, 0.3 mmol) in ethanol (2 mL). The resultant mixture was stirred for 2 h. During this period a precipitate formed. The precipitate was collected by filtration, washed with cold ethanol (15 mL) and water (30 mL), and dried under vacuum to yield **4** as an orange powder (79.5 mg, 48.9%); ^{1}H NMR (400 MHz, DMSO-d_6): δ 9.22 (s, 2H), 8.47 (d, 2H), 8.16 (s, 2H), 7.87 (d, 2H), 7.75 (dd, 2H), 7.51 (t, 4H), 7.37 (dd, 2H), 7.12 (dd, 2H), 7.03 – 6.99 (m, 2H), 6.87 (d, 2H), 6.62 – 6.58 (m, 2H), 6.47 (dd, 2H), 6.32 (d, 2H); IR (solid, ATR, cm^{-1}): 1611, 1461, 1374, 1496, 1307, 1267, 1159, 1111, 1036, 817, 738, 638, 538, 404; HR ESI-MS: Calcd. for $C_{44}H_{29}Ga_2N_4O_6I_2$ $[M+H]^+$ 1102.8685 a.m.u. Found $[M+H]^+$ 1102.8684 a.m.u.; Anal. Calcd. for $C_{44}H_{28}Ga_2N_4O_6I_2$ (%): C 47.96; H 2.56; N 5.08. Found: C 47.90; H 2.52; N 5.01.

X-ray crystallography. The crystal data for all compounds are compiled in Tables S1 and S4. Crystals were mounted in inert oil on micromounts and examined using a Bruker D8 Quest diffractometer with a Photon III detector and a microfocus source with Cu-Kα radiation (λ = 1.54178) at 150(2) K. Intensities were integrated from data recorded on 1° frames by ω or φ rotation. A multi-scan method absorption correction with a beam profile was applied.[1] The structures were solved using SHELXS[2] or SHELXT;[3] the datasets were refined by full-matrix least-squares on reflections with $F^2 \geq 2\sigma(F^2)$ values, with anisotropic displacement parameters for all non-hydrogen atoms, and with constrained riding hydrogen geometries;[2] U_{iso}(H) was set at 1.2 (1.5 for methyl groups) times U_{eq} of the parent atom. The largest features in final difference syntheses were close to heavy atoms and were of no chemical significance. SHELX[2,3] was employed through OLEX2 for structure solution and refinement.[4] The CCDC deposition numbers 2419224-2419227 contain the supplementary crystallographic data. This data can be obtained free of charge via The Cambridge Crystallography Data Centre.

Measurement of water-octanol partition coefficient (LogP). The LogP value for **1-4** was determined using the shake-flask method and UV-vis spectroscopy. The 1-octanol used in this experiment was pre-saturated with water. A DMSO solution of **1-4** (10 µL, 10 mM) was incubated with 1-octanol (495 µL) and H_2O (495 µL) in a 1.5 mL tube. The tube was shaken at room temperature for 24 h. The two phases were separated by centrifugation and the content of **1-4** in the water and 1-octanol phases was determined by UV-vis spectroscopy.

Bone mineral absorption studies. The gallium(III) complexes **1-4** (1 mM, 100 µL) were incubated with hydroxyapatite (10 mg) in HEPES buffer (900 µL) at 37 °C and agitated (200 rpm). At specific time points throughout the course of 48 h, 100 µL aliquots were removed, diluted with HEPES buffer (900 µL), and centrifuged (500 rpm). A portion of the resultant supernatant (500 µL) was digested with 65% HNO_3 overnight (1 mL). All samples were diluted 4.25-fold with water and analysed using inductively coupled plasma mass spectrometry (ICP-MS, Thermo Scientific iCAP-Qc quadrupole) to determine the amount of **1-4** unbound to hydroxyapatite. Results are presented as the mean of four determinations for each data point.

Cell Lines and Cell Culture Conditions. The U2OS bone osteosarcoma cell line was acquired from American Type Culture Collection (ATCC, Manassas, VA, USA) and cultured in Dulbecco's Modified Eagle's Medium (DMEM) supplemented with 10% fetal bovine serum

and 1% penicillin. The cells were grown at 310 K in a humidified atmosphere containing 5% CO_2. To gain access to OSC-enriched cells, a full T75 flask of U2OS cells was treated with methotrexate (300 nM) for 4 days.[5] The cells (labelled U2OS-MTX cells) were then used immediately.

Cytotoxicity MTT assay. The colourimetric MTT assay was used to determine the toxicity of test compounds. U2OS and U2OS-MTX cells (5×10^3) were seeded in each well of a 96-well plate. After incubating the cells overnight, various concentrations of the compounds (0.0004-100 µM) were added and incubated for 72 h (total volume 200 µL). Stock solutions of the compounds were prepared as 10 mM solutions in DMSO and diluted using media. The final concentration of DMSO in each well was ≥ 0.5% and this amount was present in the untreated control as well. After 72 h, 20 µL of a 4 mg/mL solution of MTT in PBS was added to each well, and the plate was incubated for an additional 4 h. The DMEM/MTT mixture was aspirated and 200 µL of DMSO was added to dissolve the resulting purple formazan crystals. The absorbance of the solutions in each well was read at 550 nm. Absorbance values were normalized to (DMSO-containing) control wells and plotted as concentration of test compound versus % cell viability. IC_{50} values were interpolated from the resulting dose dependent curves. The reported IC_{50} values are the average of three independent experiments, each consisting of six replicates per concentration level (overall n = 18). The Student's t-test was used to statistically analysis and compare data.

Sarcosphere Formation and Viability Assay. U2OS-MTX cells (2.5×10^4) were plated in ultralow-attachment 96-well plates (Corning) and incubated in DMEM supplemented with N2 (Invitrogen), human EGF (10 ng/mL), and human bFGF (10 ng/mL) for 10 days. Studies were also conducted in the presence of test compounds (0-133 µM). Sarcospheres treated with test compounds (at their respective IC_{20} values, 10 days) were imaged using an inverted microscope. The viability of the sarcospheres was determined by addition of a resazurin-based reagent, TOX8 (Sigma). After incubation for 16 h, the fluorescence of the solutions was read at 590 nm (λ_{ex} = 560 nm). Viable sarcospheres reduce the amount of the oxidized TOX8 form (blue) and concurrently increases the amount of the fluorescent TOX8 intermediate (red), indicating the degree of sarcosphere cytotoxicity caused by the test compound. Fluorescence values were normalized to DMSO-containing controls and plotted as concentration of test compound versus % sarcosphere viability. IC_{50} values were interpolated from the resulting dose dependent curves. The reported IC_{50} values are the average of three independent experiments, each consisting of three replicates per concentration level (overall n = 9). The Student's t-test was used to statistically analysis and compare data.

Cellular Uptake. To measure the cellular uptake of **1-4**, *ca.* 1 million U2OS cells were treated with **1-4** (5 µM) at 37 ºC for 24 h. After incubation, the media was removed and the cells were washed with PBS (2 mL × 3), and harvested. The number of cells was counted at this stage, using a haemocytometer. This mitigates any cell death induced by **1-4** at the administered concentration and experimental cell loss. The cells were centrifuged to form pellets. The cellular pellets were dissolved in 65% HNO_3 (250 µL) overnight to determine the whole cell uptake of **1-4**. For **2**, the gallium content in the cytoplasmic, nuclear, and membrane fractions was also determined. The Thermo Scientific NE-PER Nuclear and Cytoplasmic Extraction Kit was used to extract and separate the cytoplasmic, nuclear, and membrane fractions. The fractions were dissolved in 65% HNO_3 (250 µL final volume) overnight. All samples were diluted 17-fold with water and analysed using inductively coupled plasma mass spectrometry (ICP-MS, Thermo Scientific ICAP-Qc quadrupole ICP mass spectrometer). Gallium levels are

expressed as Ga (ng) per million cells. Results are presented as the mean of four determinations for each data point.

Annexin V-propidium iodide assay. U2OS cells were incubated with and without **2** (2 × IC_{50} value and 4 × IC_{50} value for 72 h) and cisplatin (50 µM for 72 h) at 37 °C. Cells were harvested from adherent cultures by trypsinisation. The FITC Annexin V/Dead Cell Apoptosis Kit was used. The manufacture's (Thermo Fisher Scientific) protocol was followed to carry out this experiment. Briefly, untreated and treated cells (1 × 10^6) were suspended in 1× Annexin binding buffer (100 µL) (10 mM HEPES, 140 mM NaCl, 2.5 mM $CaCl_2$, pH 7.4), then 5 µL of FITC Annexin V and 1 µL of PI (100 µg/ mL) were added to each sample and incubated at room temperature for 15 min. After which more 1× Annexin binding buffer (400 µL) was added while gently mixing. The cells were analysed using a FACSCanto II flow cytometer (BD Biosciences) (10,000 events per sample were acquired) at the University of Leicester FACS Facility. The FL1 channel was used to assess Annexin V binding and the FL2 channel was used to assess PI uptake. Cell populations were analysed using FlowJo.

Immunoblotting analysis. U2OS cells (1 × 10^6) were incubated with **2** (2.1-8.2 µM µM for 72 h or 4.1-16.4 µM for 4 h) at 37 °C. U2OS cells were harvested and isolated as pellets. SDS-PAGE loading buffer (64 mM Tris-HCl (pH 6.8), 9.6% glycerol, 2% SDS, 5% β-mercaptoethanol, 0.01% bromophenol blue) was added to the pellets, and this was incubated at 95 °C for 10 min. Cell lysates were resolved by 4-20 % sodium dodecylsulphate polyacylamide gel electrophoresis (SDS-PAGE; 200 V for 25 min) followed by electro transfer to polyvinylidene difluoride membrane, PVDF (350 mA for 1 h). Membranes were blocked in 5% (w/v) non-fat milk in PBST (PBS/0.1% Tween 20) and incubated with the appropriate primary antibodies (Cell Signalling Technology). After incubation with horseradish peroxidase-conjugated secondary antibodies (Cell Signalling Technology), immune complexes were detected with the ECL detection reagent (BioRad) and analysed using a chemiluminescence imager (Bio-Rad ChemiDoc Imaging System).

Cytoplasmic calcium levels. U2OS cells (2.5 × 10^4) were seeded in each well of a 96-well plate. After incubating the cells overnight, they were treated with **2** (2 × IC_{50} value) or co-treated with **2** (2 × IC_{50} value) and cycloheximide (1 µM) for 24 h. After the incubation period, the media was removed and 120 µL of Hanks′ Balanced Salt solution (HBSS) with 20 mM HEPES was added to each well. Then 40 µL of a 20 µM solution of the Fluo-4 AM dye was added to each well, and the plate was incubated for an additional 1 h. The staining solution was then removed and the replaced with 50 µL of fresh Hanks′ Balanced Salt solution (HBSS) with 20 mM HEPES. The washing solution was removed and 50 µL of fresh Hanks′ Balanced Salt solution (HBSS) with 20 mM HEPES was added. The cytoplasmic calcium level was determined by measuring the fluorescence of the solutions in each well at 526 nm (λ_{ex} = 490 nm). The Student's t-test was used to statistically analysis and compare data.

Intracellular ROS assay. U2OS cells (5 × 10^3) were seeded in each well of a 96-well plate. After incubating the cells overnight, they were treated with **2** (2 × IC_{50} value or 4 × IC_{50} value for 0.5-24 h), and incubated with 6-carboxy-2′,7′-dichlorodihydrofluorescein diacetate (20 µM) for 30 min. The intracellular ROS level was determined by measuring the fluorescence of the solutions in each well at 529 nm (λ_{ex} = 504 nm). The Student's t-test was used to statistically analysis and compare data.

CRT cell surface exposure. Flow cytometry was used to analyse cell surface CRT exposure. U2OS cells were seeded into a 6-well plate (at a density of 5 × 10^5 cells/ mL) and the cells

were incubated at 37 °C overnight. The cells were treated with **2** (2 × IC_{50} value or 4 × IC_{50} value) or co-treated with cisplatin (150 μM) with thapsigargin (7 μM) for 24 h at 37 °C. The cells were then harvested by trypsinization and collected by centrifugation. The resulting pellets were suspended in PBS (500 μL), and after the addition of the Alexa Fluor® 488 nm labelled anti-CRT antibody (2 μL), the cells were incubated in the dark for 30 minutes. The cells were then washed with PBS (1 mL) and analysed using a FACSCanto II flow cytometer (BD Biosciences) (10,000 events per sample were acquired) at the University of Leicester FACS Facility. The FL1 channel was used to assess CRT cell surface exposure. Cell populations were analysed using Floreada.io.

ATP assay. U2OS cells (5 × 10^3 cells /well) were seeded in a 96-well plate and incubated overnight. The cells were then treated with **2** (2 × IC_{50} value or 4 × IC_{50} value) or cisplatin (50 μM, positive control) for 24 h at 37 °C. The media was carefully extracted and transferred into a white-walled opaque 96-well plate, and a luciferin-based ENLITEN ATP Assay Kit (Promega) was used to measure the relative amount of ATP released into the supernatant.

HMGB-1 release. U2OS cells (1 × 10^6 cells) were incubated with **2** (2.1-8.2 μM μM for 72 h) at 37 °C. Cells were collected in full and added to SDS-PAGE loading buffer (64 mM Tris-HCl (pH 6.8), 9.6% glycerol, 2% SDS, 5% β-mercaptoethanol, 0.01% Bromophenol Blue) and incubated at 95 °C for 10 min. The HMGB-1 content was probed by immunoblotting analysis as described above. The anti-HMGB-1 antibody (Cell Signalling Technology) was used in this experiment.

Phagocytosis assay. U2OS or U2OS-MTX cells were seeded into a 6-well plate (at a density of 5 × 10^5 cells/ mL) and the cells were incubated at 37 °C overnight. The cells were stained with CellTracker Green (30 min) and washed with DMEM media. The cells were then treated with **2** (25-50 μM) or cisplatin (150 μM) with thapsigargin (7 μM) for 24 h at 37 °C. Then macrophages, obtained by differentiating THP-1 cells with phorbol 12-myristate 13-acetate (100 nM for 72 h) and pre-stained with CellTracker Orange for 30 min and washed with RPMI 1640 media, were added to the U2OS or U2OS-MTX cells (at a density of 5 × 10^5 cells/ mL). After 2 h, phagocytosis was assessed by flow cytometry, using a FACSCanto II flow cytometer (BD Biosciences) (10,000 events per sample were acquired) at the University of Leicester FACS Facility. The FL1 channel was used to assess the CellTracker Green-stained U2OS or U2OS-MTX cell population and the FL4 channel was used to assess the CellTracker Orange-stained THP-1 macrophage population. Cell populations were analysed using Floreada.io.

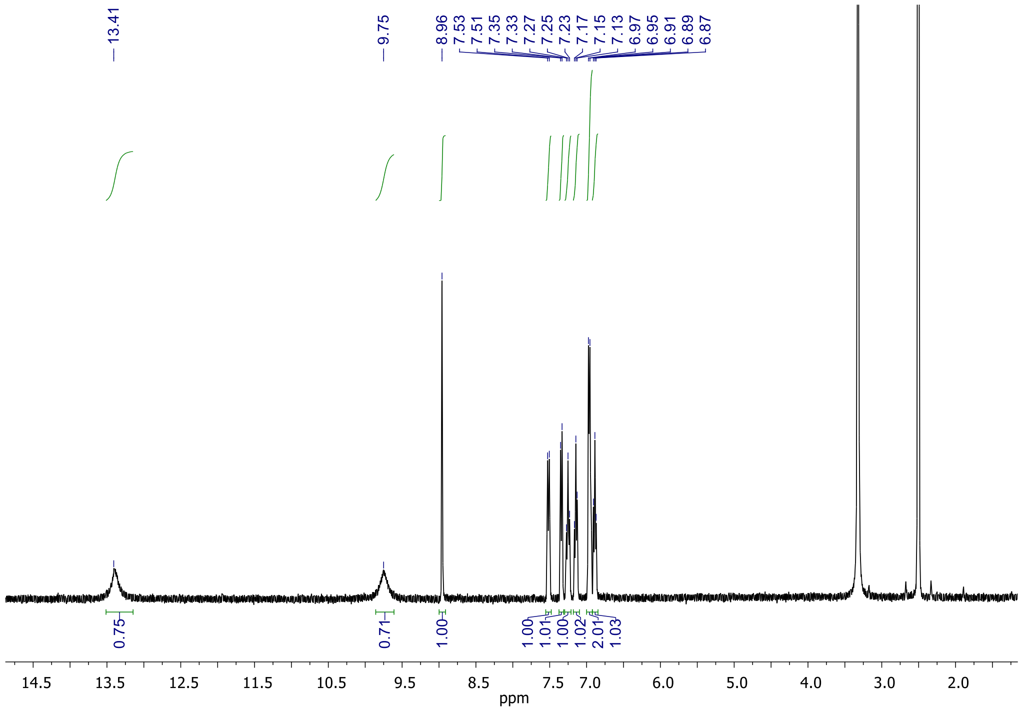

Figure S1. ^{1}H NMR spectrum (400 MHz, DMSO-d_6) of **L**$^{\mathbf{1}}$.

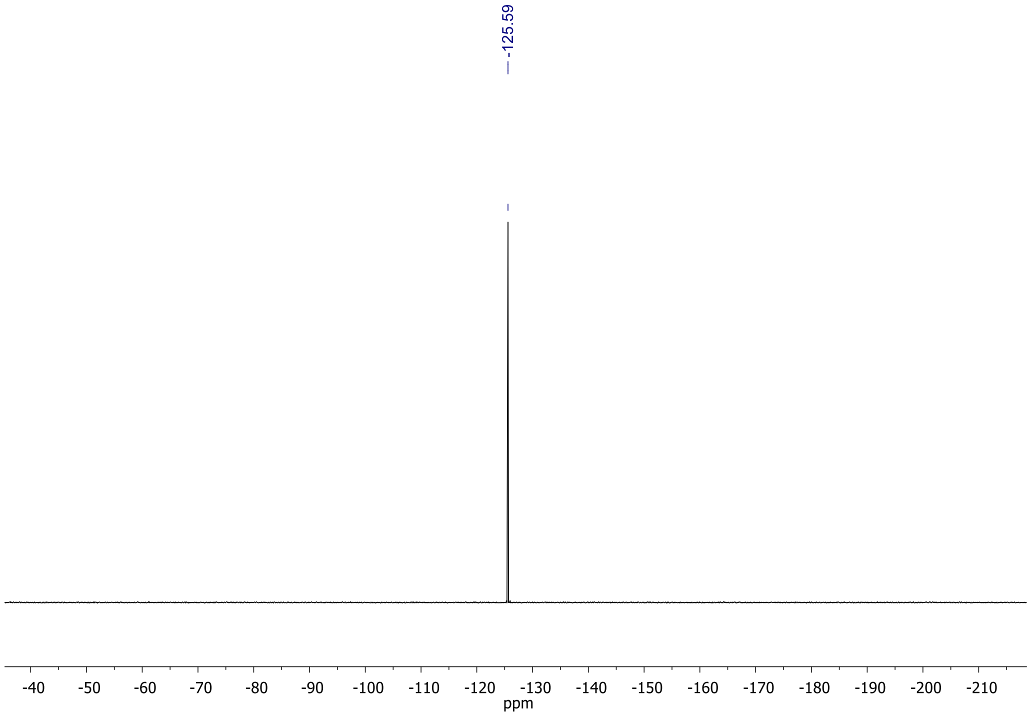

Figure S2. $^{19}F\{^{1}H\}$ NMR spectrum (376 MHz, DMSO-d_6) of **L**$^{\mathbf{1}}$.

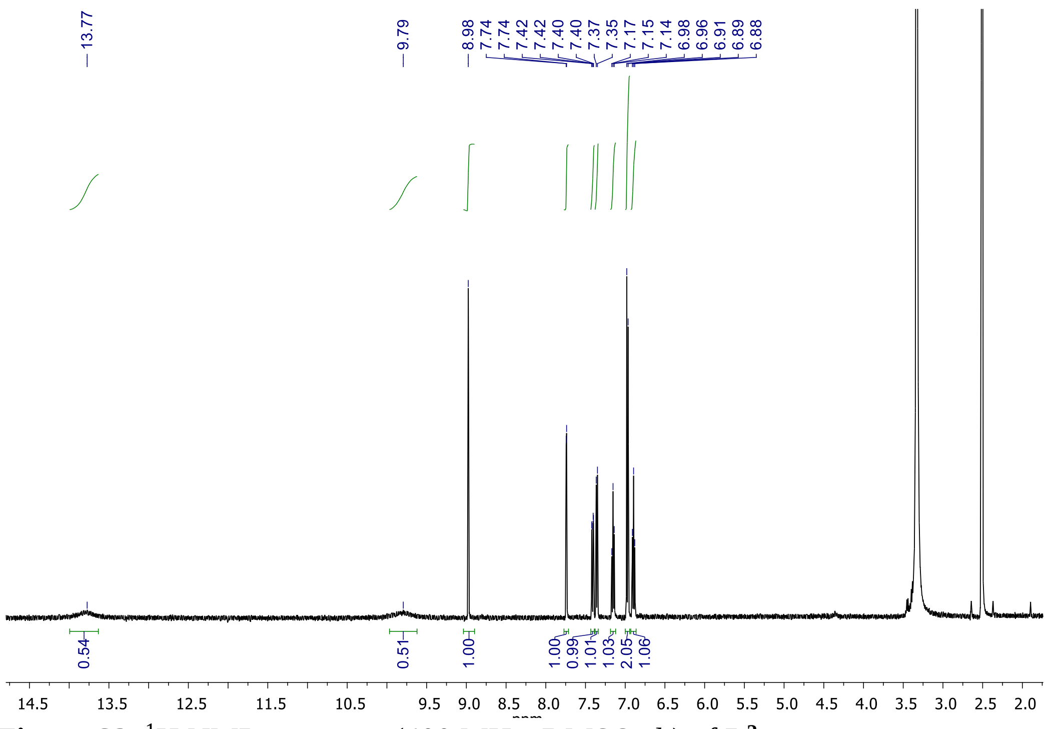

Figure S3. ^{1}H NMR spectrum (400 MHz, DMSO-d_6) of **L^2**.

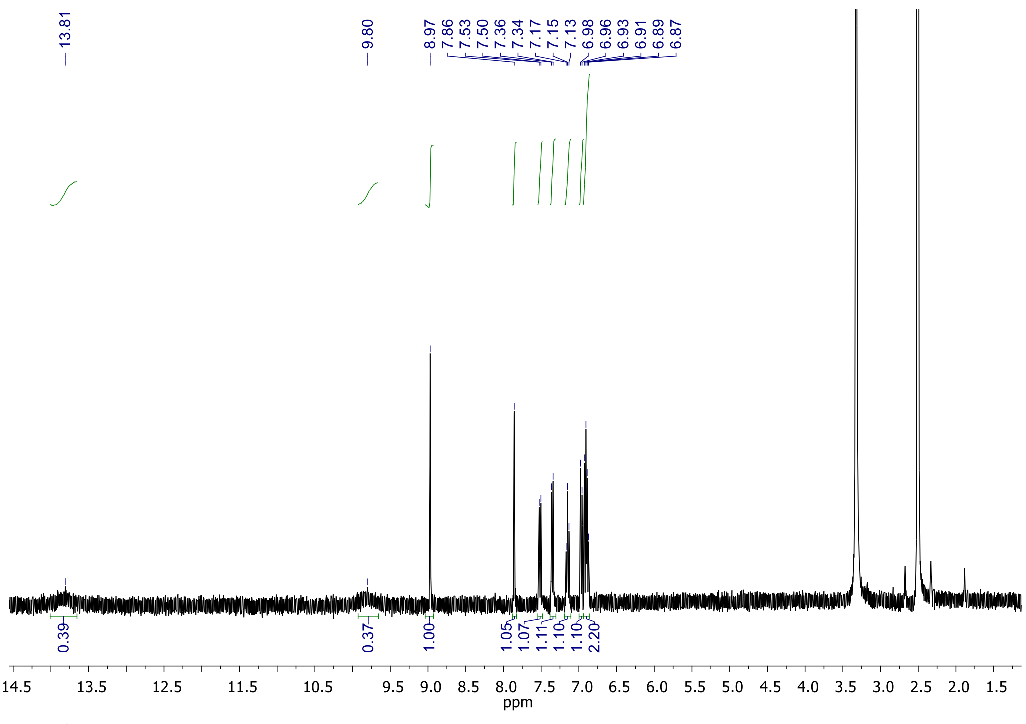

Figure S4. ^{1}H NMR spectrum (400 MHz, DMSO-d_6) of **L^3**.

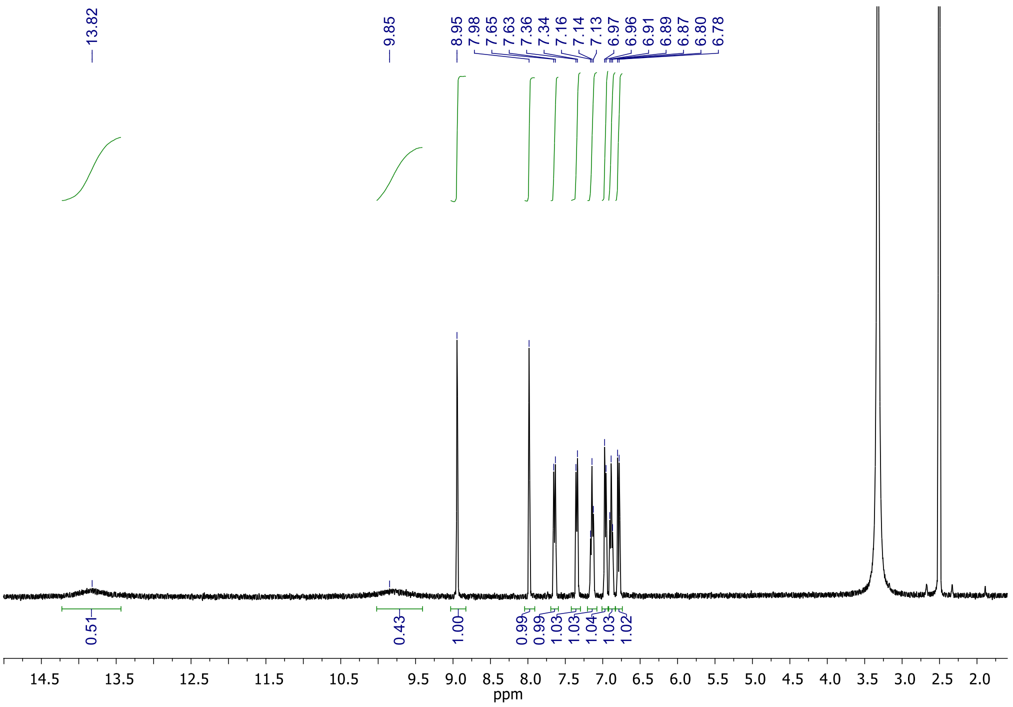

Figure S5. ^{1}H NMR spectrum (400 MHz, DMSO-d_6) of $\mathbf{L^4}$.

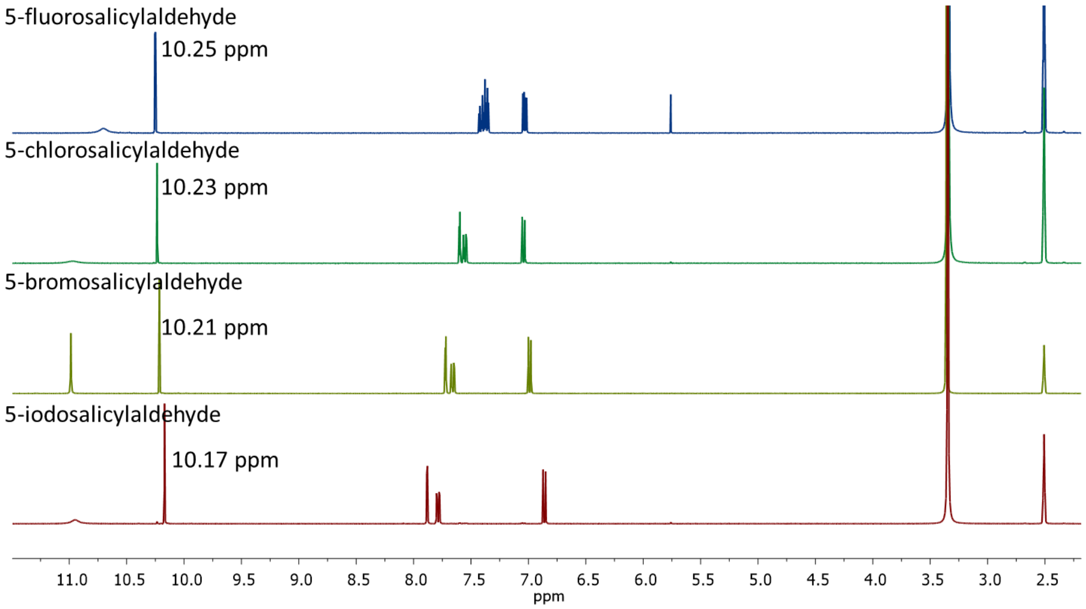

Figure S6. ^{1}H NMR spectra (400 MHz, DMSO-d_6) of 5-fluorosalicylaldehyde, 5-chlorosalicylaldehyde, 5-bromosalicylaldehyde, and 5-iodosalicylaldehyde. The aldehyde proton peak is highlighted.

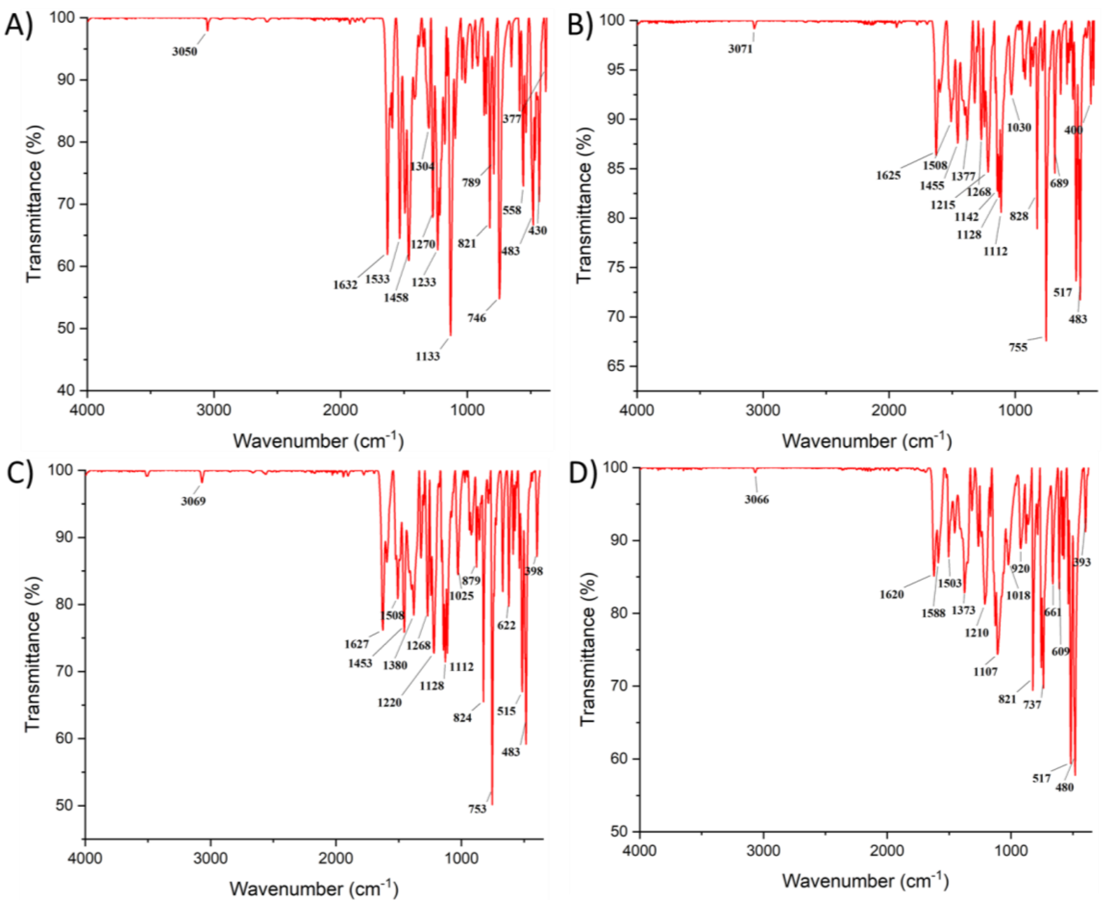

Figure S7. ATR-FTIR spectra of (A) $\mathbf{L^1}$, (B) $\mathbf{L^2}$, (C) $\mathbf{L^3}$, and (D) $\mathbf{L^4}$ in the solid form.

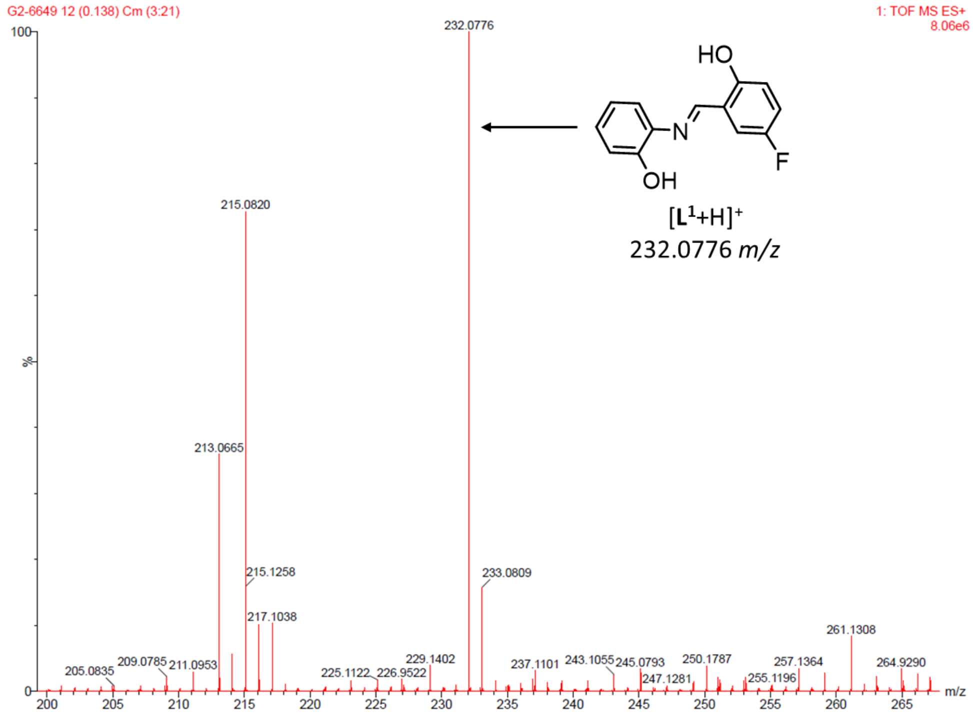

Figure S8. High resolution ESI mass spectrum (positive mode) of $\mathbf{L^1}$.

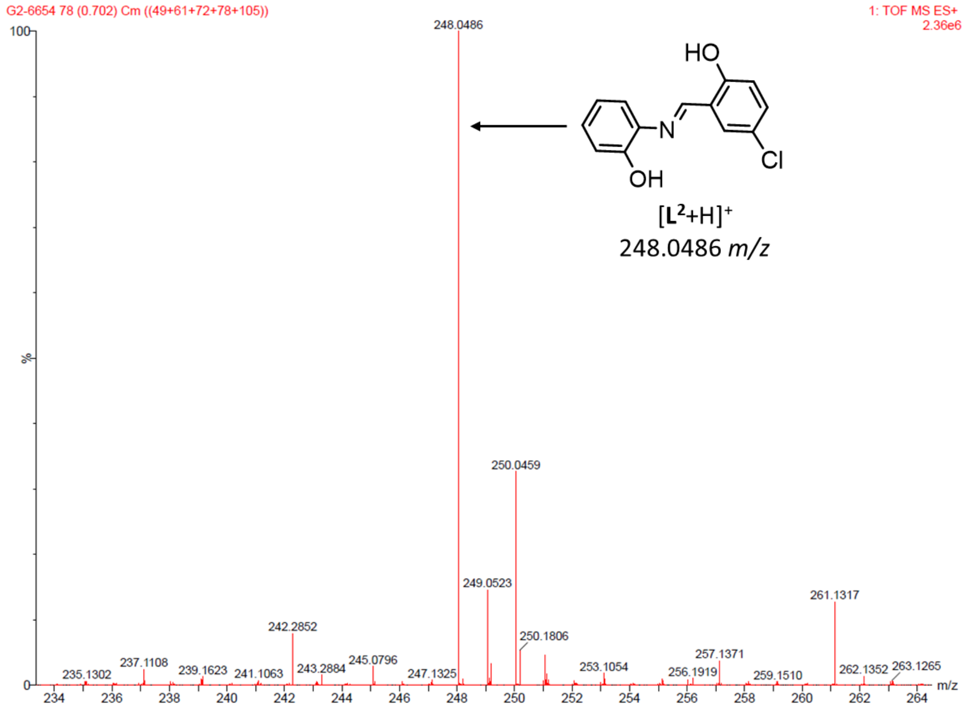

Figure S9. High resolution ESI mass spectrum (positive mode) of $\mathbf{L^2}$.

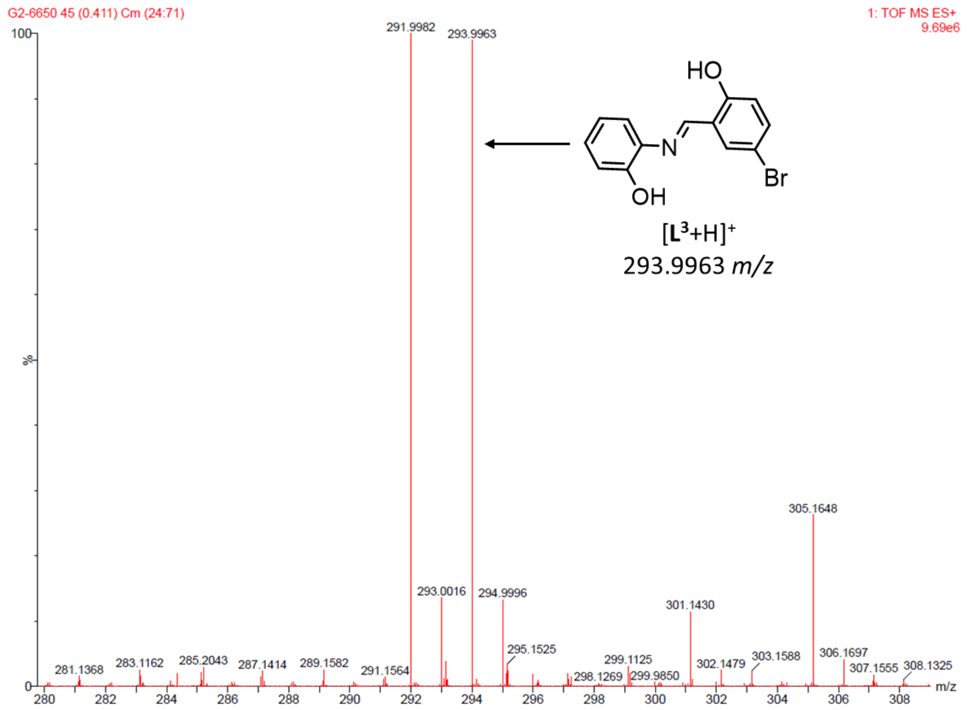

Figure S10. High resolution ESI mass spectrum (positive mode) of $\mathbf{L^3}$.

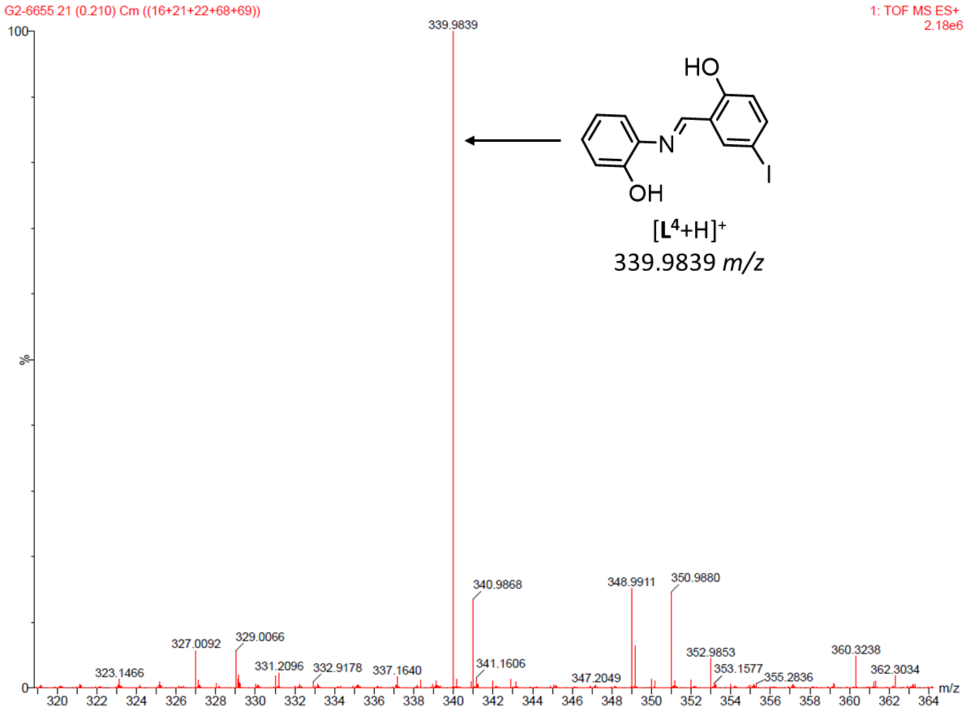

Figure S11. High resolution ESI mass spectrum (positive mode) of **L⁴**.

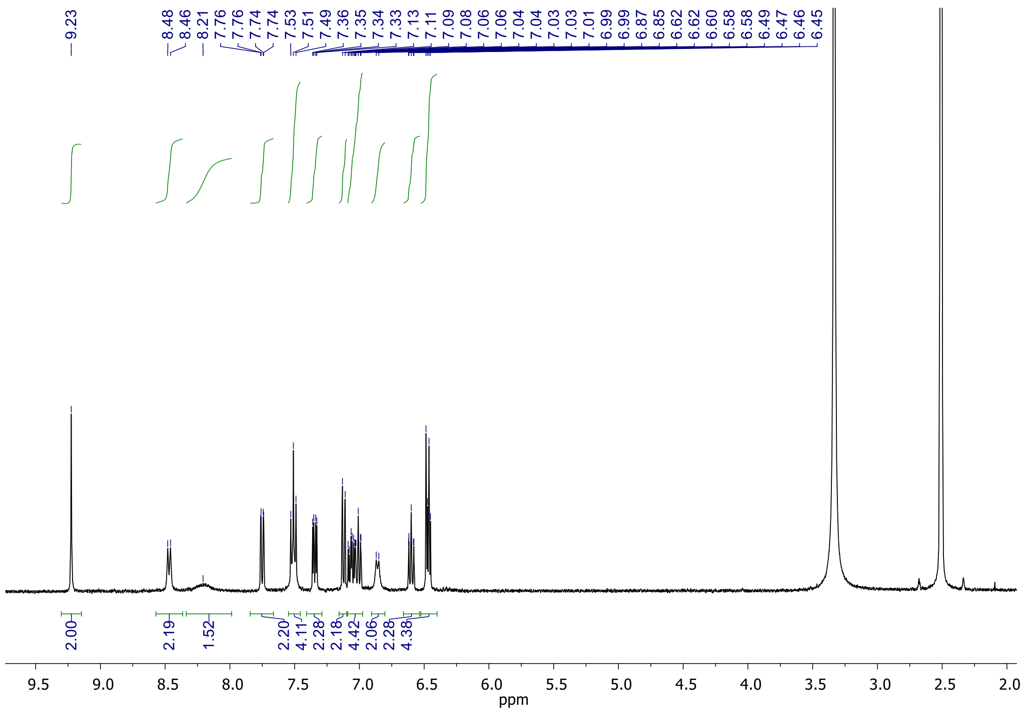

Figure S12. ^{1}H NMR spectrum (400 MHz, DMSO-d_6) of **1**.

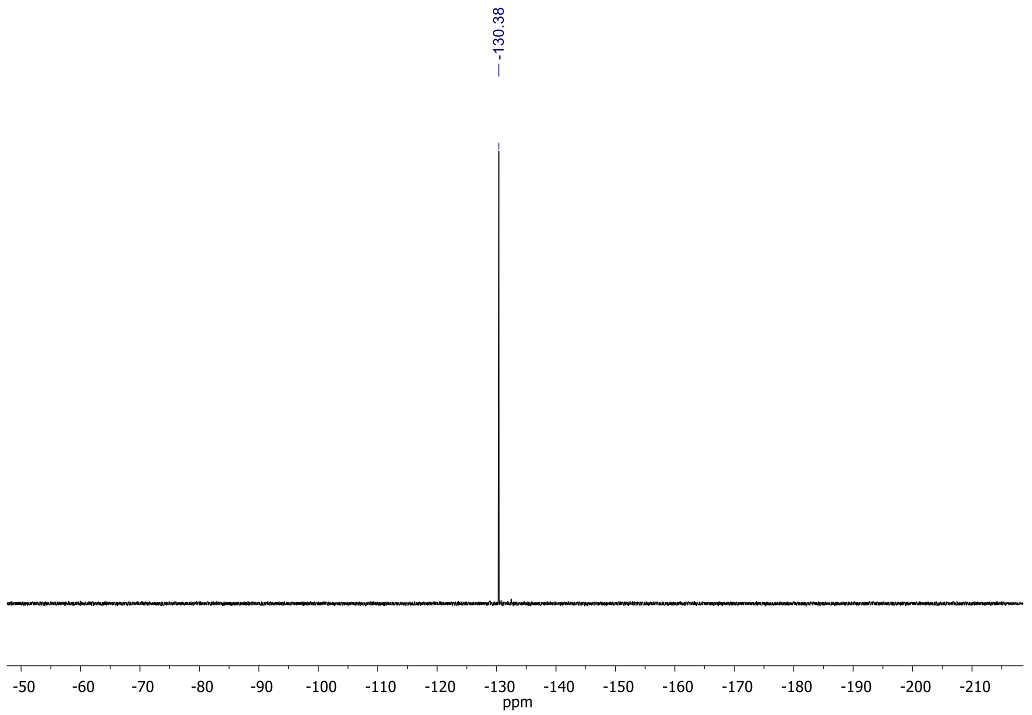

Figure S13. ${}^{19}F\{{}^{1}H\}$ NMR spectrum (376 MHz, DMSO-d_6) of **1**.

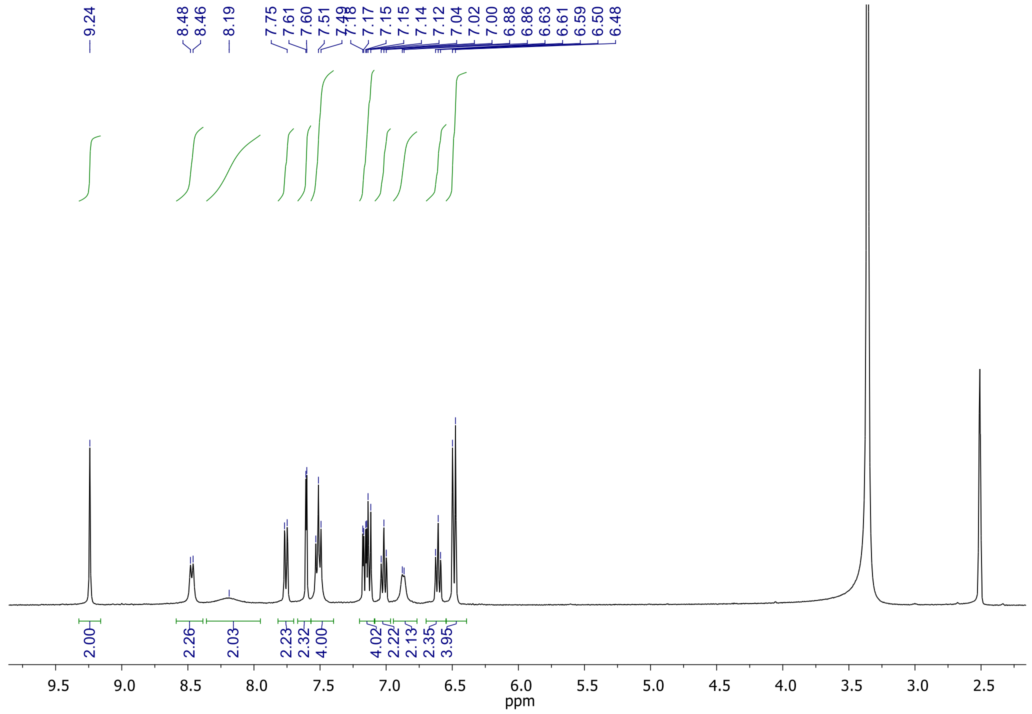

Figure S14. ${}^{1}H$ NMR spectrum (400 MHz, DMSO-d_6) of **2**.

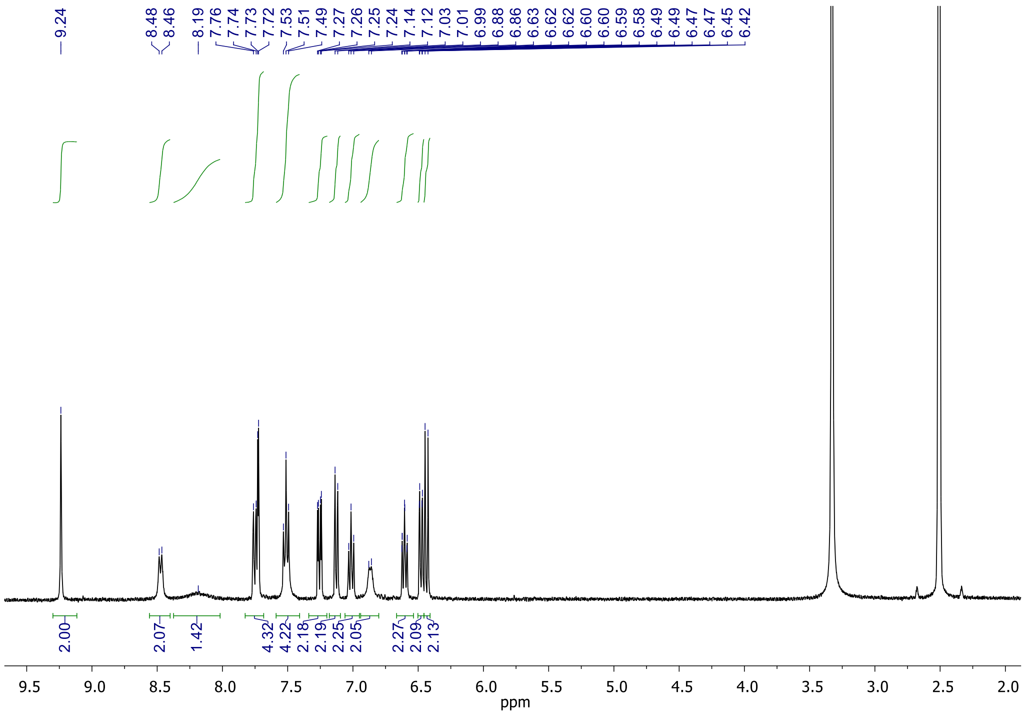

Figure S15. ^{1}H NMR spectrum (400 MHz, DMSO-d_6) of **3**.

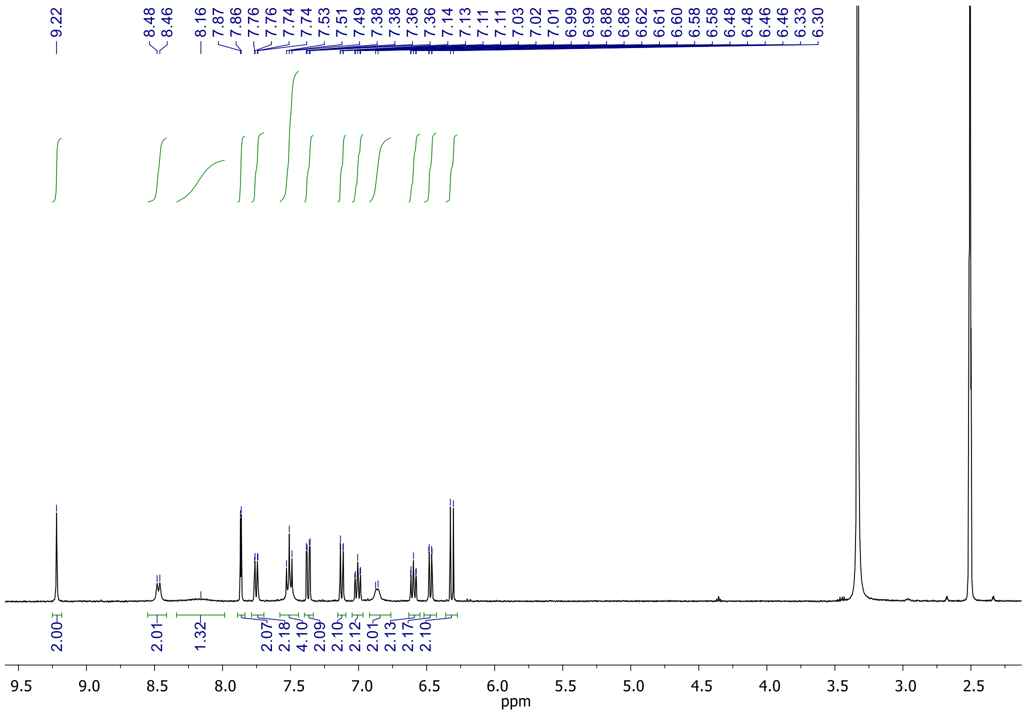

Figure S16. ^{1}H NMR spectrum (400 MHz, DMSO-d_6) of **4**.

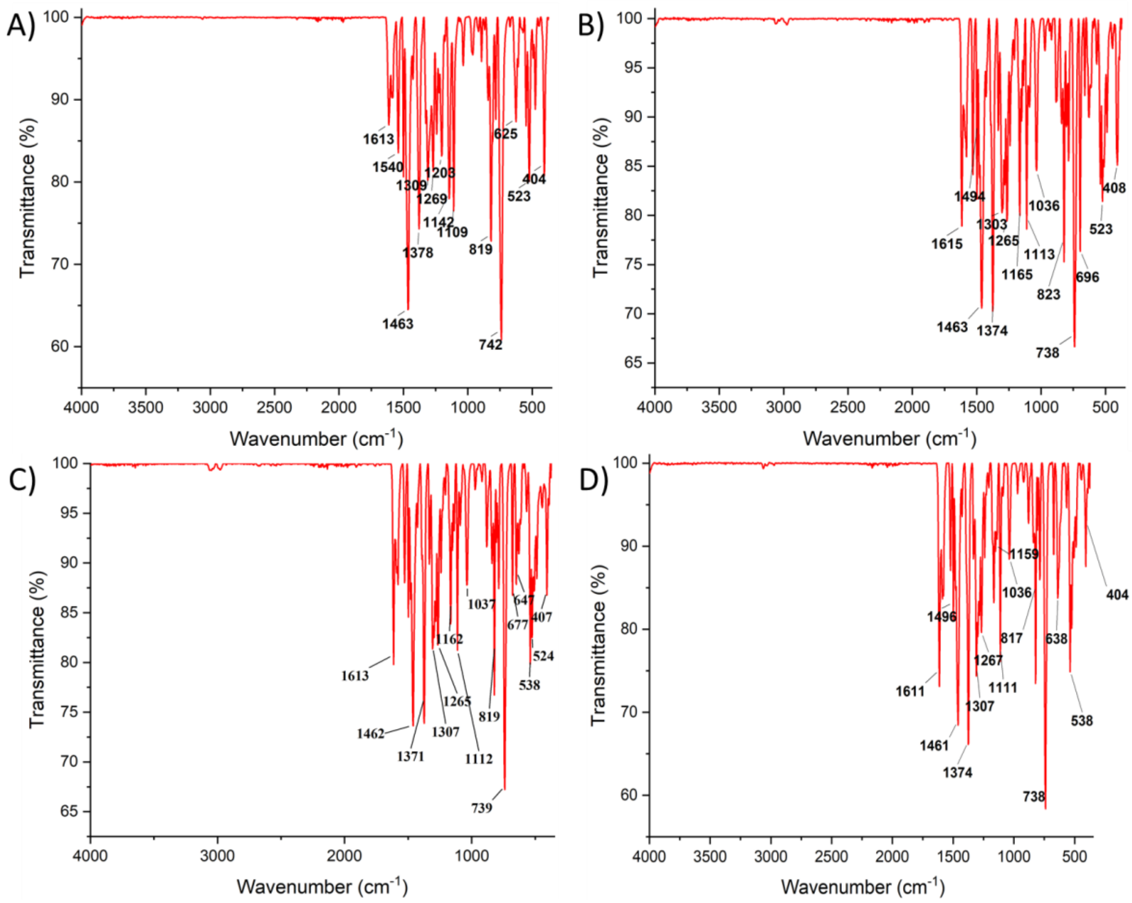

Figure S17. ATR-FTIR spectra of (A) **1**, (B) **2**, (C) **3**, and (D) **4** in the solid form.

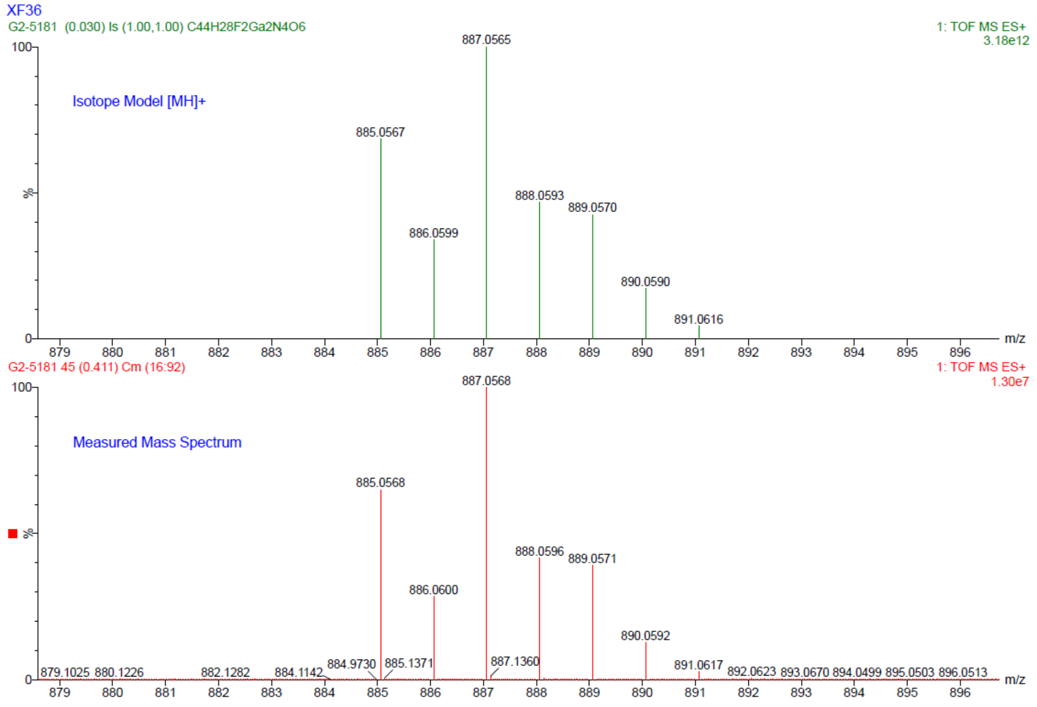

Figure S18. (Top) Theoretical isotope model for [**1**+H]$^{+}$ ($C_{44}H_{29}Ga_2N_4O_6F_2$) and (bottom) the experimentally determined high-resolution ESI-TOF mass spectrum for complex **1**.

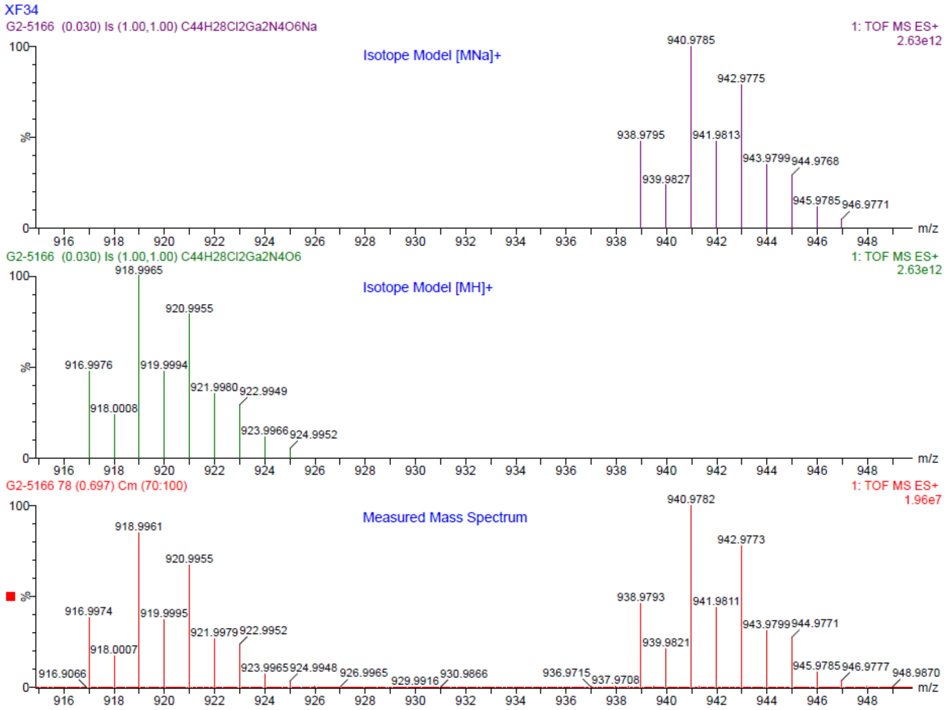

Figure S19. (Top and middle) Theoretical isotope model for [**2**+Na]$^+$ ($C_{44}H_{28}Ga_2N_4O_6Cl_2Na$) and [**2**+H]$^+$ ($C_{44}H_{29}Ga_2N_4O_6Cl_2$) and (bottom) the experimentally determined high-resolution ESI-TOF mass spectrum for complex **2**.

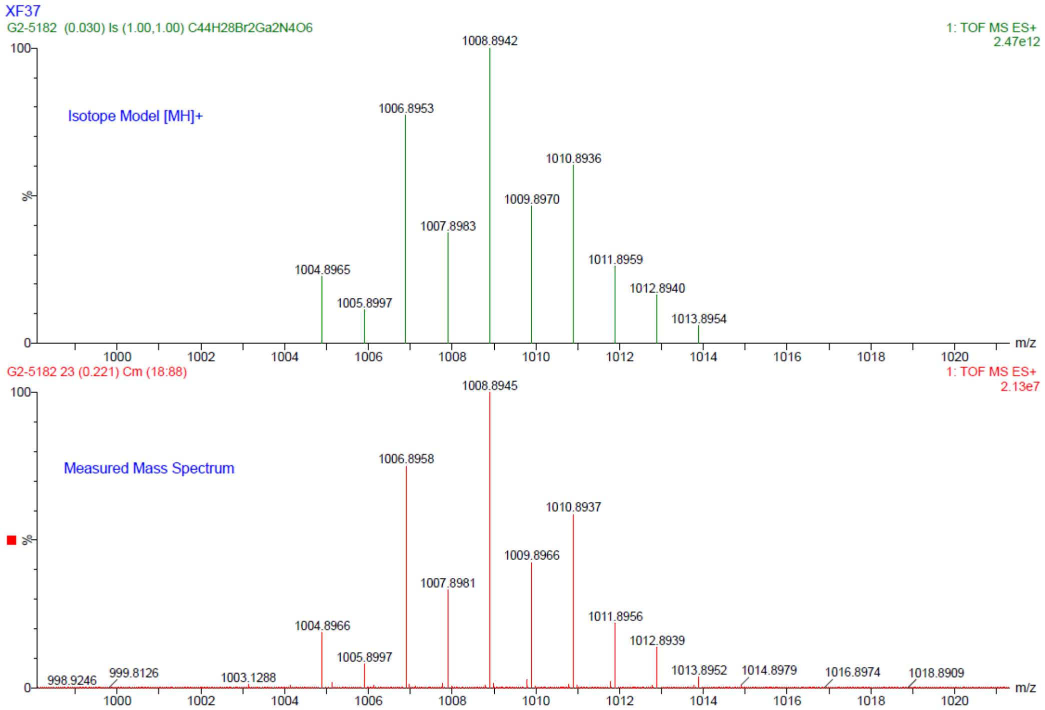

Figure S20. (Top) Theoretical isotope model for [**3**+H]$^+$ ($C_{44}H_{29}Ga_2N_4O_6Br_2$) and (bottom) the experimentally determined high-resolution ESI-TOF mass spectrum for complex **3**.

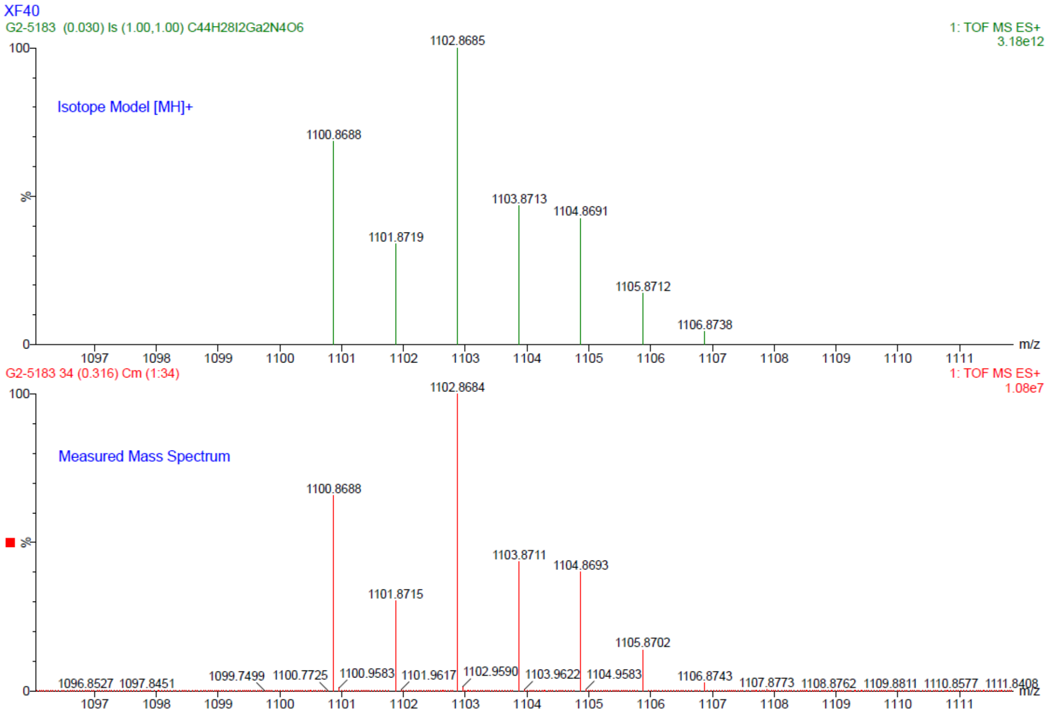

Figure S21. (Top) Theoretical isotope model for [**4**+H]$^+$ ($C_{44}H_{29}Ga_2N_4O_6I_2$) and (bottom) the experimentally determined high-resolution ESI-TOF mass spectrum for complex **4**.

Table S1. Crystallographic data for complexes **1** and **2**.

**Metal complex**	**1**	**2**
CCDC No.	2419225	2419226
formula	$C_{44}H_{28}Ga_2N_4O_6F_2$•$2C_2H_3N$	$C_{44}H_{28}Cl_2Ga_2N_4O_6$
Fw	968.25	919.04
Crystal system	monoclinic	triclinic
Space group	$P2_1/c$	*P*-1
a, Å	11.8634(4)	12.647(3)
b, Å	22.9459(7)	16.411(3)
c, Å	15.7208(4)	18.069(3)
α, deg.	90	93.171(11)
β, deg.	106.591(2)	94.571(13)
γ, deg.	90	90.673(12)
V, Å^3	4101.3(2)	3732.2(12)
Z	4	4
D_{calcd}, Mg/m^3	1.568	1.636
2θ / deg.	7.018 to 144.752	4.914 to 130.61
Reflections collected	54095	42127
Independent reflections	8089	12615
Goodness-of-fit on F^2	1.018	0.96
R_1, wR_2 [$I \geq 2\sigma(I)$]	0.0524, 0.1247	0.1362, 0.2890
R_1, wR_2 [all data]	0.0829, 0.1402	0.3403, 0.3956

Table S2. Selected bond lengths (Å) and angles (°) for complex **1**.

Ga(1)-O(1)	1.908(3)	Ga(2)-O(3)	2.027(3)
Ga(1)-O(2)	1.929(3)	Ga(2)-O(4)	1.916(3)
Ga(1)-O(3)	2.041(3)	Ga(2)-O(5)	1.929(3)
Ga(1)-O(6)	1.965(3)	Ga(2)-O(6)	2.066(2)
Ga(1)-N(4)	2.090(3)	Ga(2)-N(3)	2.024(3)
Ga(1)-N(1)	2.031(4)	Ga(2)-N(2)	2.037(4)
O(1)-Ga(1)-O(2)	172.88(12)	O(3)-Ga(2)-O(6)	74.86(10)
O(1)-Ga(1)-O(3)	92.41(12)	O(3)-Ga(2)-N(2)	88.91(15)
O(1)-Ga(1)-O(6)	93.46(11)	O(4)-Ga(2)-O(3)	163.83(11)
O(1)-Ga(1)-N(4)	91.02(13)	O(4)-Ga(2)-O(5)	100.03(13)
O(1)-Ga(1)-N(1)	92.86(14)	O(4)-Ga(2)-O(6)	93.65(12)
O(2)-Ga(1)-O(3)	89.08(12)	O(4)-Ga(2)-N(3)	95.60(12)
O(2)-Ga(1)-O(6)	93.66(12)	O(4)-Ga(2)-N(2)	79.75(16)
O(2)-Ga(1)-N(4)	90.35(13)	O(5)-Ga(2)-O(3)	92.59(12)
O(2)-Ga(1)-N(1)	80.02(15)	O(5)-Ga(2)-O(6)	165.51(12)
O(3)-Ga(1)-N(4)	156.83(12)	O(5)-Ga(2)-N(3)	83.38(12)
O(6)-Ga(1)-O(3)	76.76(11)	O(5)-Ga(2)-N(2)	96.29(15)
O(6)-Ga(1)-N(4)	80.16(11)	N(3)-Ga(2)-O(3)	95.87(12)
O(6)-Ga(1)-N(1)	173.64(14)	N(3)-Ga(2)-O(6)	90.61(11)
N(1)-Ga(1)-O(3)	103.66(14)	N(3)-Ga(2)-N(2)	175.22(16)
N(1)-Ga(1)-N(4)	99.05(14)	N(2)-Ga(2)-O(6)	90.77(14)

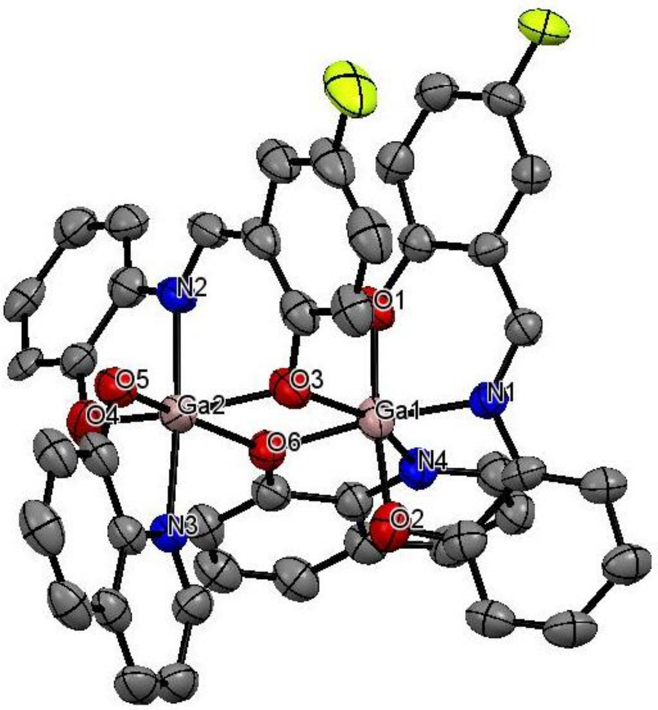

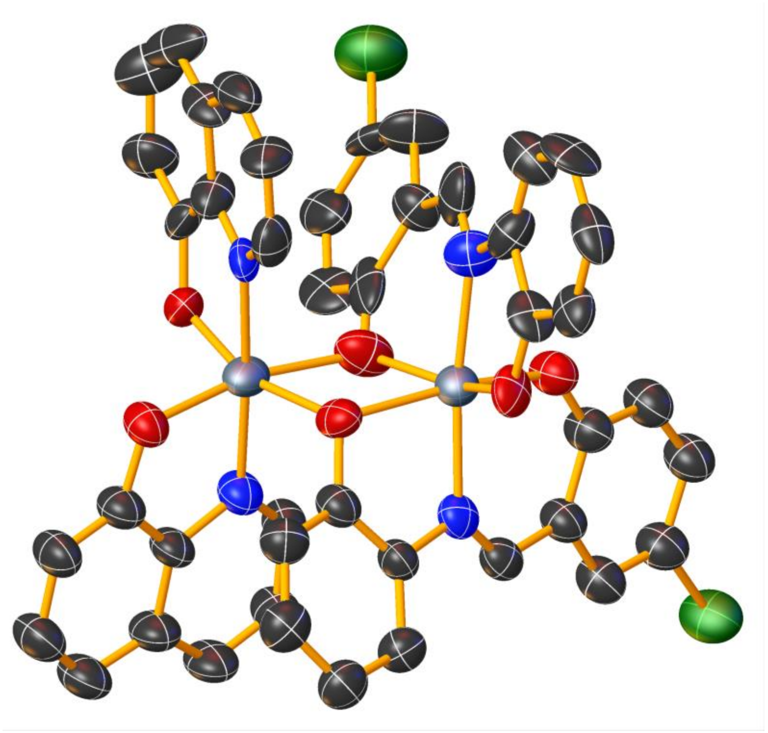

Figure S22. X-ray structure of **2**. Ellipsoids are shown at 50% probability, H atoms have been omitted for clarity. C in black, N in dark blue, O in red, Cl in green, and Ga in grey.

Table S3. Experimentally determined LogP values for **1-4**.

Ga(III) complex	LogP
**1**	1.51 ± 0.001
**2**	1.86 ± 0.05
**3**	1.90 ± 0.002
**4**	2.10 ± 0.03

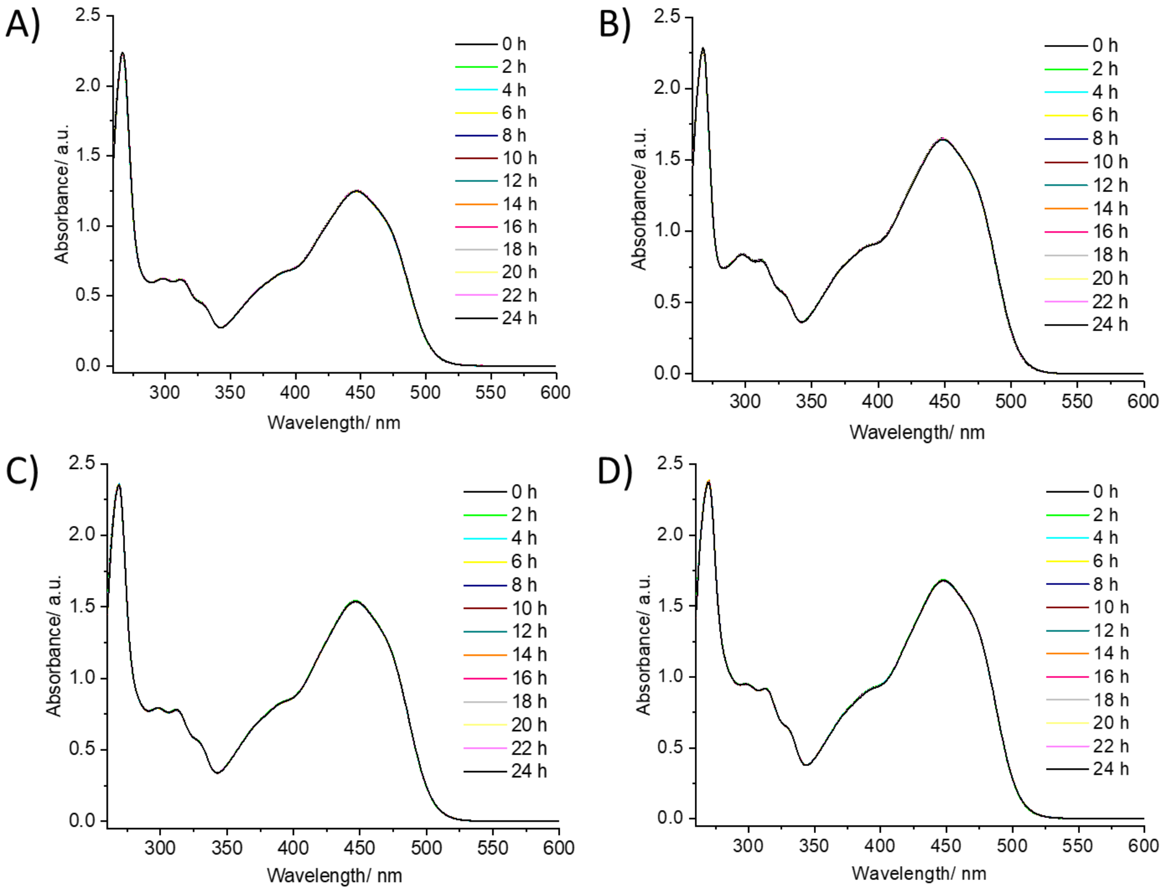

Figure S23. UV-vis spectra of (A) **1**, (B) **2**, (C) **3**, and (D) **4** (all 50 µM) in DMSO over the course of 24 h at 37 ^{o}C.

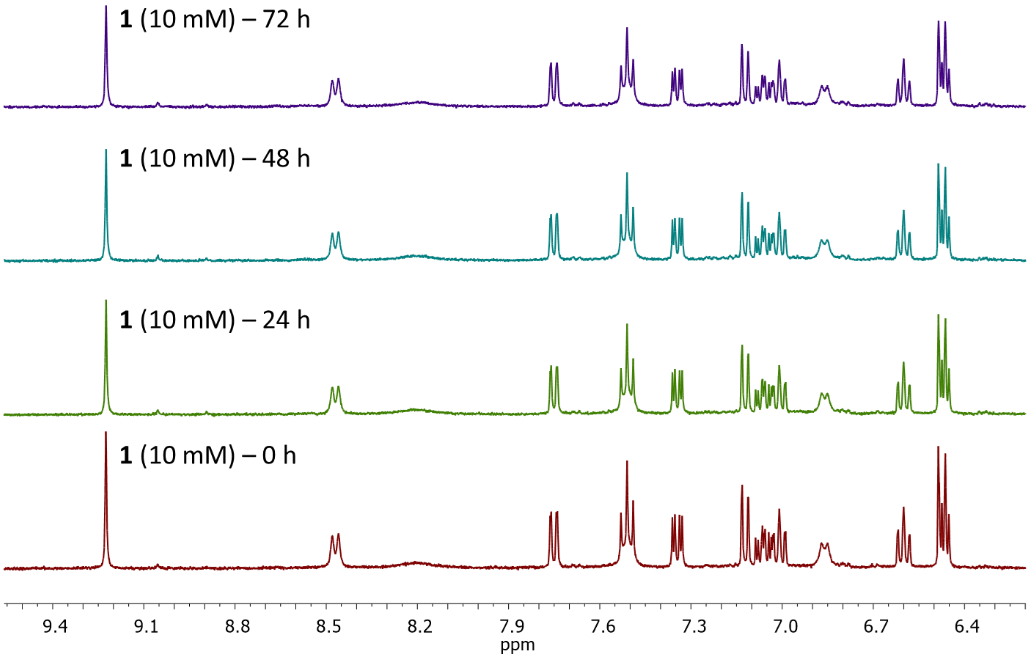

Figure S24. ^{1}H NMR spectra of **1** (10 mM) in DMSO-d_6 over the course of 72 h.

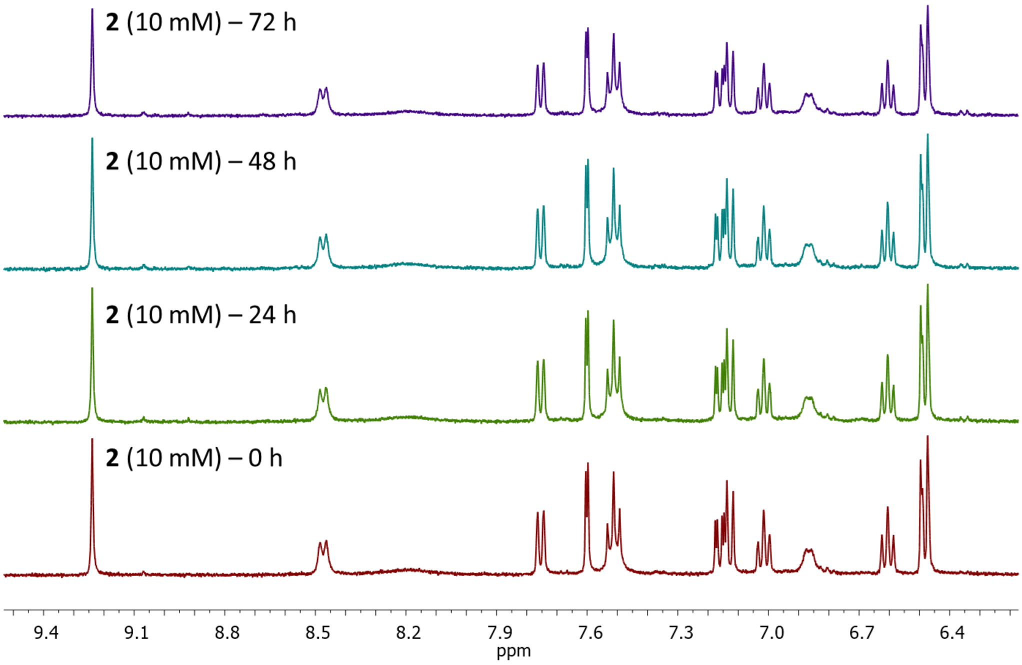

Figure S25. ^{1}H NMR spectra of **2** (10 mM) in DMSO-d_6 over the course of 72 h.

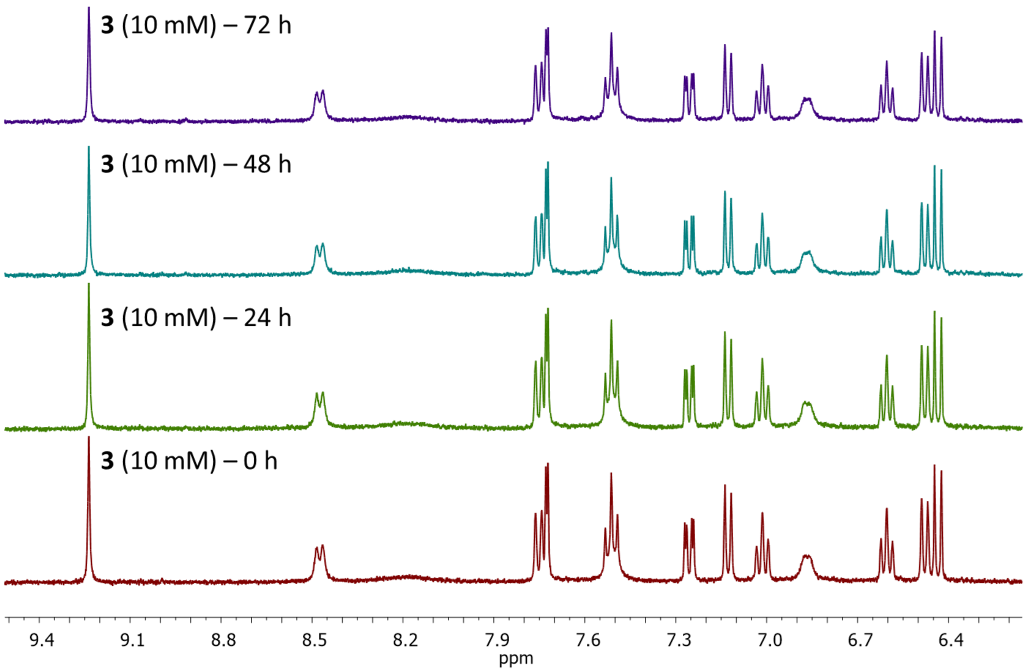

Figure S26. ^{1}H NMR spectra of **3** (10 mM) in DMSO-d_6 over the course of 72 h.

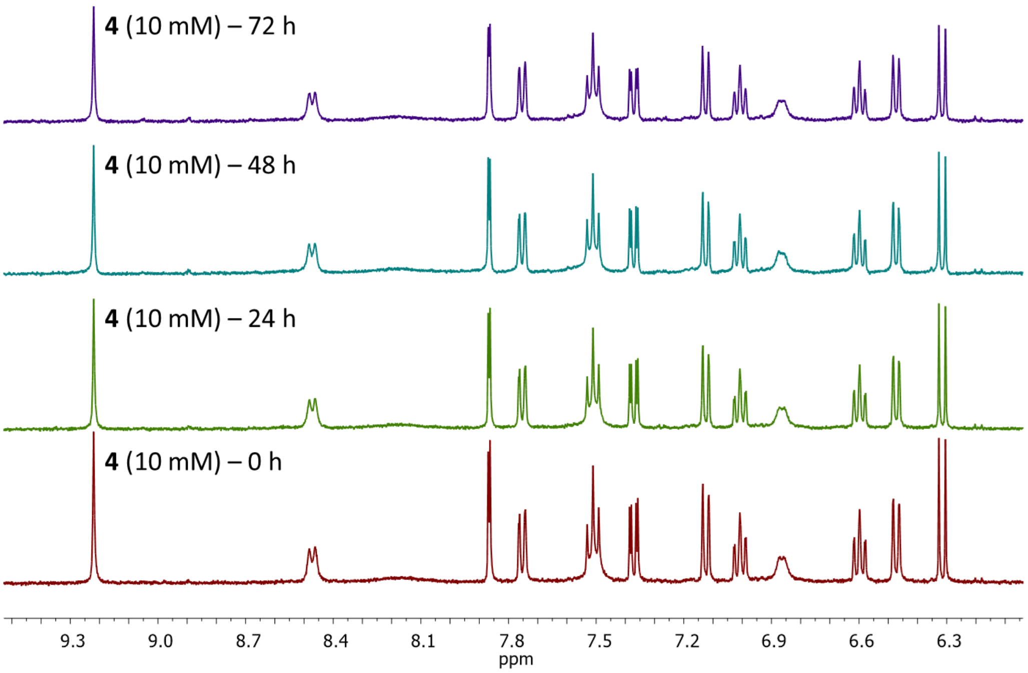

Figure S27. ^{1}H NMR spectra of **4** (10 mM) in DMSO-d_6 over the course of 72 h.

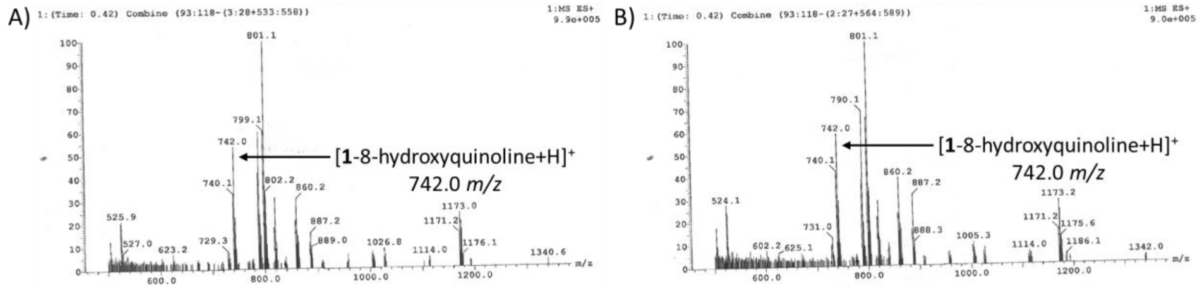

Figure S28. ESI mass spectra (positive mode) of **1** (0.5 mM) in DMSO after incubation for (A) 0 h or (B) 24 h at 37 ^{o}C.

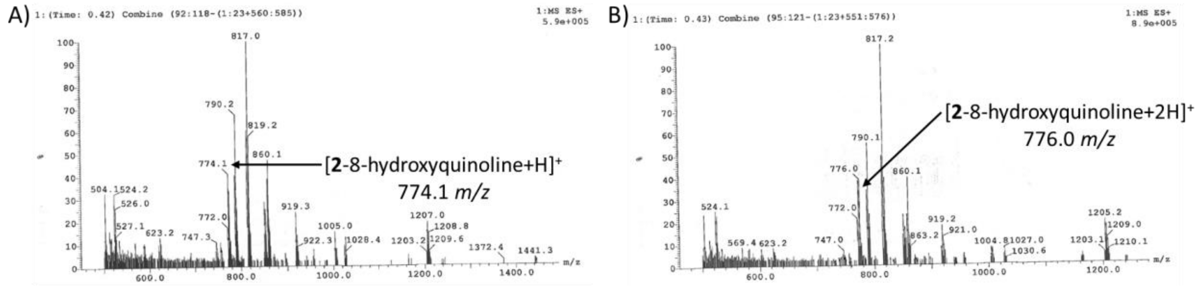

Figure S29. ESI mass spectra (positive mode) of **2** (0.5 mM) in DMSO after incubation for (A) 0 h or (B) 24 h at 37 ^{o}C.

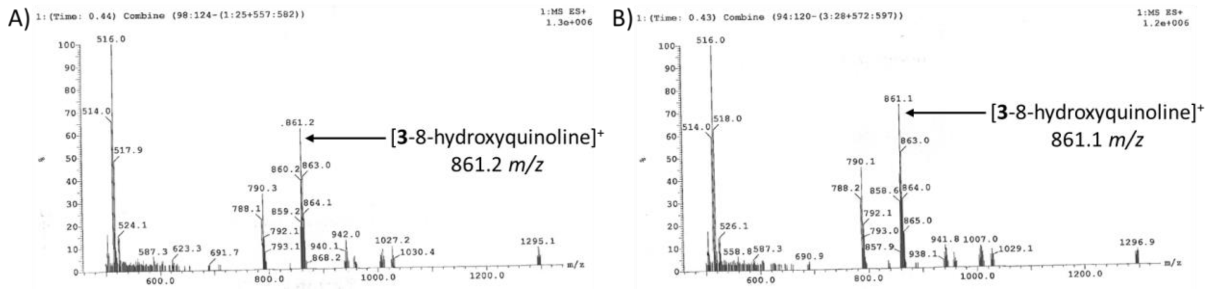

Figure S30. ESI mass spectra (positive mode) of **3** (0.5 mM) in DMSO after incubation for (A) 0 h or (B) 24 h at 37 ^{o}C.

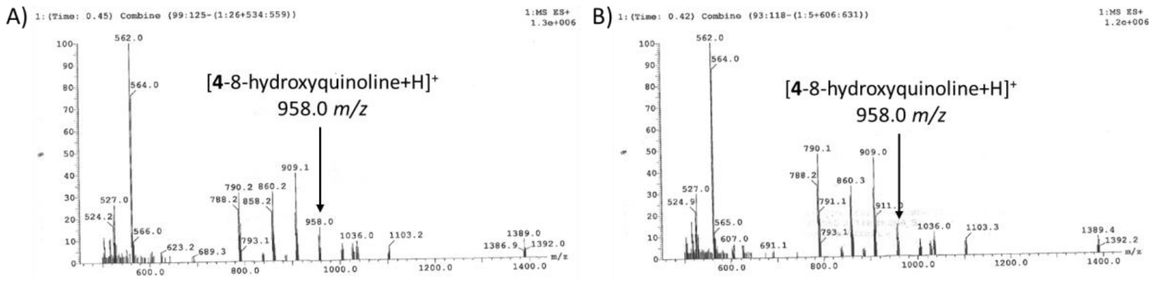

Figure S31. ESI mass spectra (positive mode) of **4** (0.5 mM) in DMSO after incubation for (A) 0 h or (B) 24 h at 37 ^{o}C.

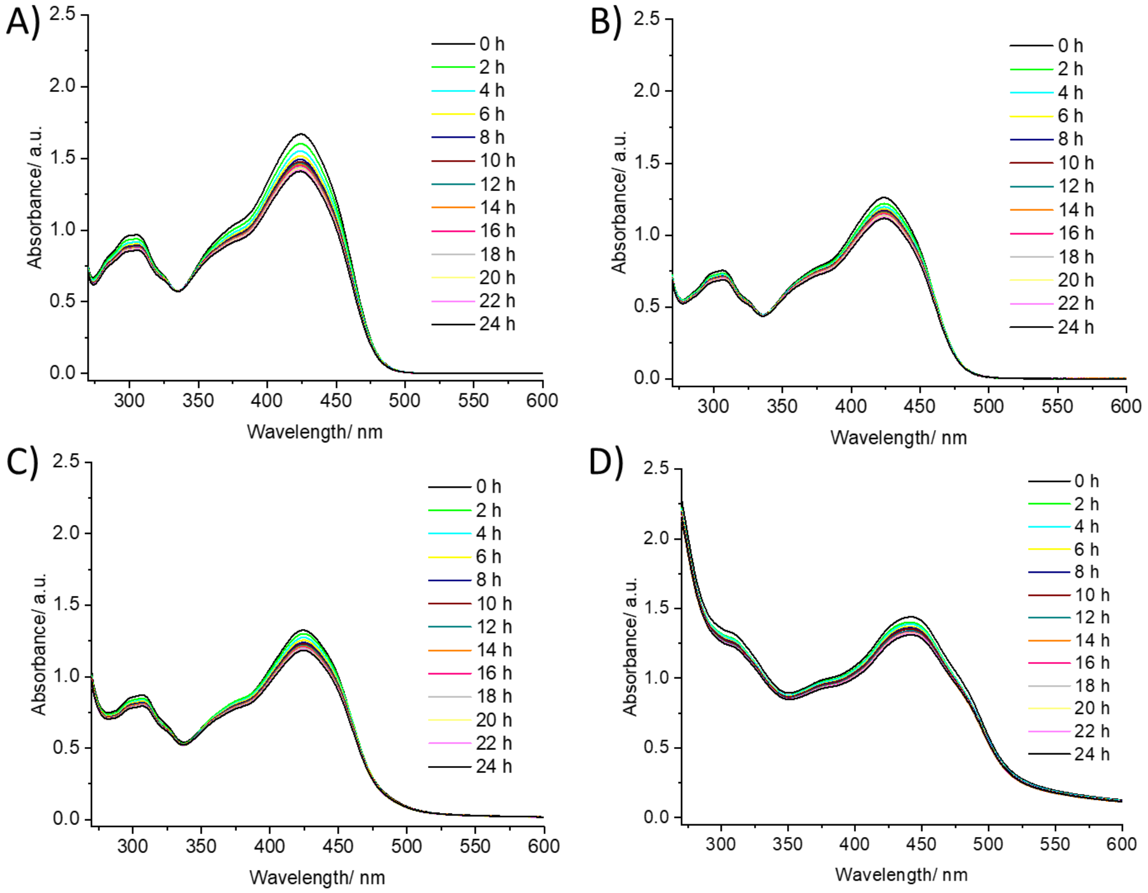

Figure S32. UV-vis spectra of (A) **1**, (B) **2**, (C) **3**, and (D) **4** (all 50 μM) in H_2O:DMSO (200:1**)** over the course of 24 h at 37 ^{o}C.

Table S4. Crystallographic data for complexes **1a** and **3a**.

**Metal complex**	**1a**	**3a**
CCDC No.	2419224	2419227
formula	$C_{24}H_{20}FGaN_2O_4S$	$C_{24}H_{20}BrGaN_2O_4S$•$0.25C_4H_{10}O$
Fw	521.20	600.64
Crystal system	monoclinic	monoclinic
Space group	Cc	$P2_1/n$
a, Å	16.0660(5)	14.9530(5)
b, Å	17.4487(5)	12.8360(4)
c, Å	8.4467(2)	25.6006(9)
α, deg.	90	90
β, deg.	112.9970(10)	99.591(2)
γ, deg.	90	90
V, $Å^3$	2179.68(11)	4845.0(3)
Z	4	8
D_{calcd}, Mg/m^3	1.588	1.647
2θ / deg.	7.836 to 145.132	6.418 to 144.256
Reflections collected	11268	78581
Independent reflections	3695	9533
Goodness-of-fit on F^2	1.052	1.165
R_1, wR_2 [$I \geq 2\sigma(I)$]	0.0423, 0.1099	0.0649, 0.1516
R_1, wR_2 [all data]	0.0425, 0.1100	0.0694, 0.1536

Table S5. Selected bond lengths (Å) and angles (°) for complex **1a**.

Ga(1)-O(1)	1.908(4)	Ga(1)-O(4)	2.012(4)
Ga(1)-O(2)	1.969(4)	Ga(1)-N(1)	2.046(4)
Ga(1)-O(3)	1.936(4)	Ga(1)-N(3)	2.124(4)
O(1)-Ga(1)-O(2)	170.68(18)	O(3)-Ga(1)-O(2)	96.40(17)
O(1)-Ga(1)-O(3)	92.89(17)	O(3)-Ga(1)-O(4)	88.52(17)
O(1)-Ga(1)-O(4)	90.21(16)	O(3)-Ga(1)-N(1)	173.36(18)
O(1)-Ga(1)-N(1)	90.58(18)	O(3)-Ga(1)-N(3)	81.46(17)
O(1)-Ga(1)-N(3)	92.03(17)	O(4)-Ga(1)-N(1)	97.14(18)
O(2)-Ga(1)-O(4)	90.77(15)	O(4)-Ga(1)-N(3)	169.83(17)
O(2)-Ga(1)-N(1)	80.10(18)	N(1)-Ga(1)-N(3)	92.76(18)
O(2)-Ga(1)-N(3)	88.62(16)		

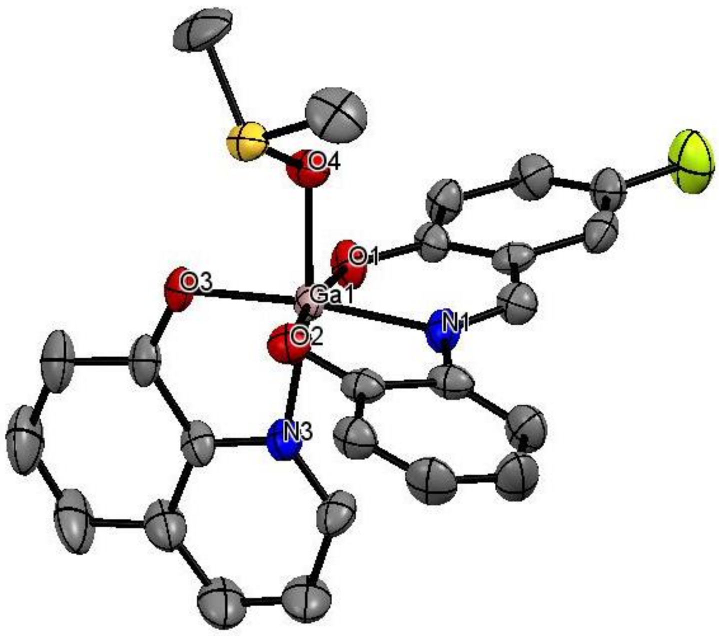

Table S6. Selected bond lengths (Å) and angles (°) for complex **3a**.

Ga(1A)-N(1A)	2.047(5)	Ga(1)-N(1)	2.054(4)
Ga(1A)-N(2A)	2.105(5)	Ga(1)-N(2)	2.113(4)
Ga(1A)-O(1A)	1.952(5)	Ga(1)-O(1)	1.933(3)
Ga(1A)-O(2A)	1.919(5)	Ga(1)-O(2)	1.963(3)
Ga(1A)-O(3A)	1.900(4)	Ga(1)-O(3)	1.929(3)
Ga(1A)-O(4A)	2.025(4)	Ga(1)-O(4)	2.025(4)
N(1A)-Ga(1A)-N(2A)	94.82(19)	O(2)-Ga(1)-N(1)	81.48(16)
O(1A)-Ga(1A)-N(1A)	81.0(2)	O(2)-Ga(1)-N(2)	93.02(15)
O(1A)-Ga(1A)-N(2A)	91.89(19)	O(2)-Ga(1)-O(4)	88.76(15)
O(1A)-Ga(1A)-O(4A)	89.9(2)	O(3)-Ga(1)-N(1)	172.37(16)
O(2A)-Ga(1A)-N(1A)	91.59(19)	O(3A)-Ga(1A)-O(1A)	95.8(2)
O(2A)-Ga(1A)-N(2A)	88.54(19)	O(3A)-Ga(1A)-O(2A)	91.55(19)
O(2A)-Ga(1A)-O(1A)	172.6(2)	O(3A)-Ga(1A)-O(4A)	90.56(18)
O(2A)-Ga(1A)-O(4A)	90.6(2)	O(4A)-Ga(1A)-N(1A)	92.4(2)
O(3A)-Ga(1A)-N(1A)	175.7(2)	O(4A)-Ga(1A)-N(2A)	172.79(19)
O(3A)-Ga(1A)-N(2A)	82.30(17)	O(3)-Ga(1)-N(2)	82.45(15)
N(1)-Ga(1)-N(2)	94.51(16)	O(3)-Ga(1)-O(1)	96.89(14)
O(1)-Ga(1)-N(1)	90.11(16)	O(3)-Ga(1)-O(2)	91.67(15)
O(1)-Ga(1)-N(2)	90.68(16)	O(3)-Ga(1)-O(4)	89.19(15)
O(1)-Ga(1)-O(2)	171.06(15)	O(4)-Ga(1)-N(1)	93.98(16)
O(1)-Ga(1)-O(4)	88.77(16)	O(4)-Ga(1)-N(2)	171.49(16)

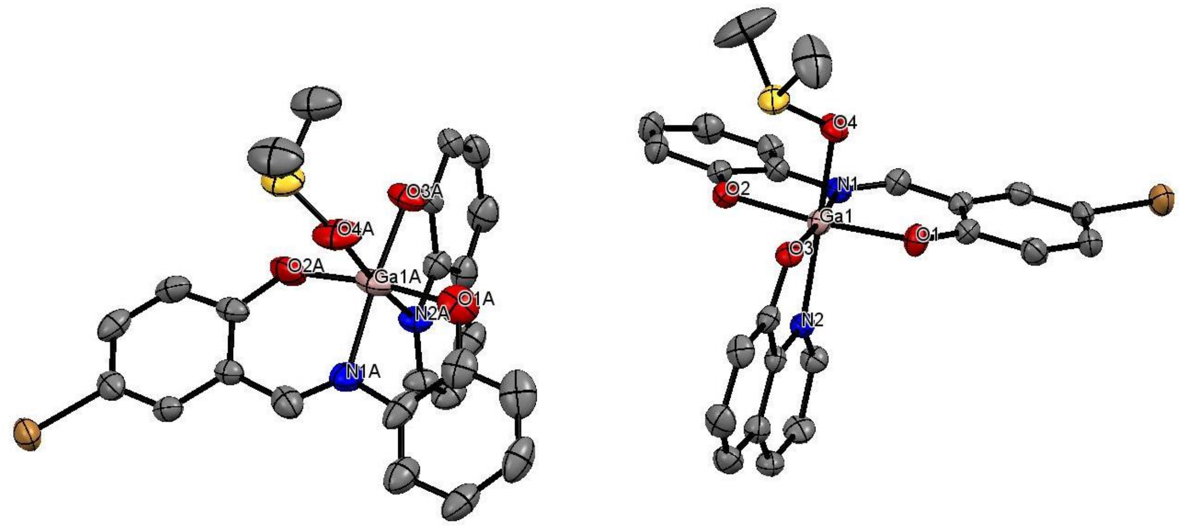

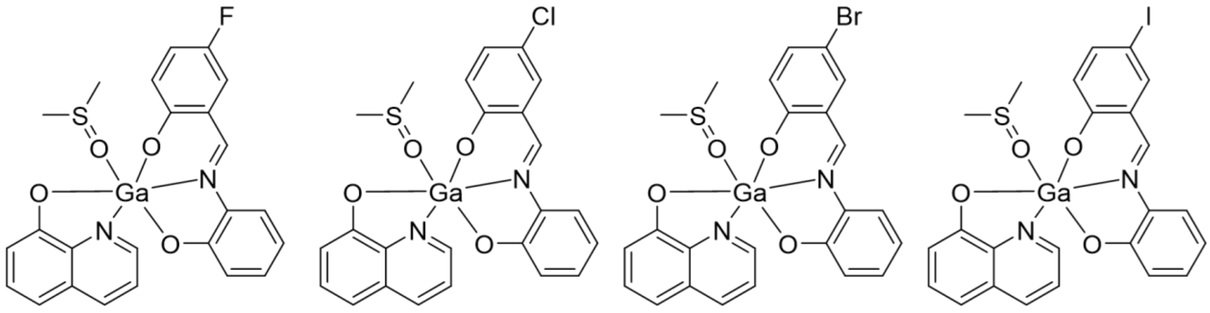

1a **2a** **3a** **4a**

Figure S33. Chemical structures of the mono-nuclear gallium(III)-DMSO complexes **1a**-**4a**. The mono-nuclear gallium(III)-DMSO complexes **1a**-**4a** are observed in small amounts in H_2O:DMSO solutions.

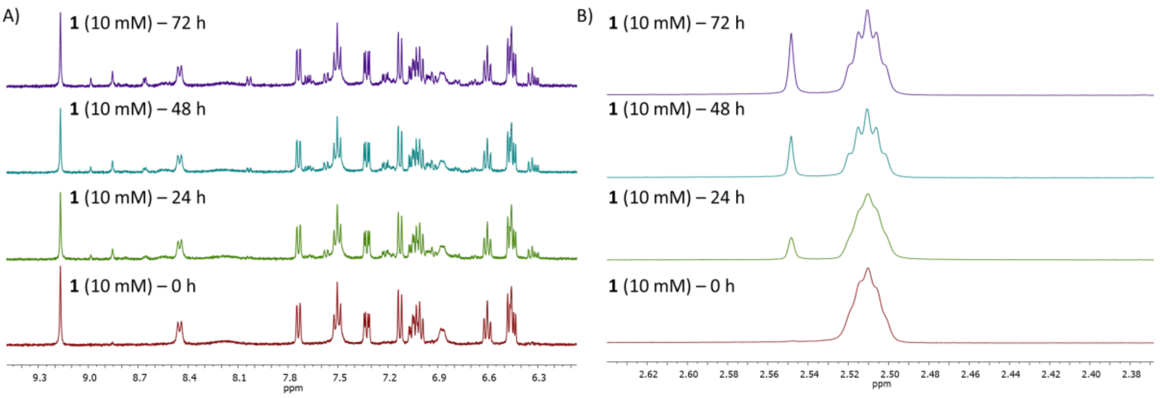

Figure S34. ^{1}H NMR spectra of **1** (10 mM) in D_2O:DMSO-d_6 (9:1) over the course of 72 h. (A) aromatic region and (B) aliphatic region.

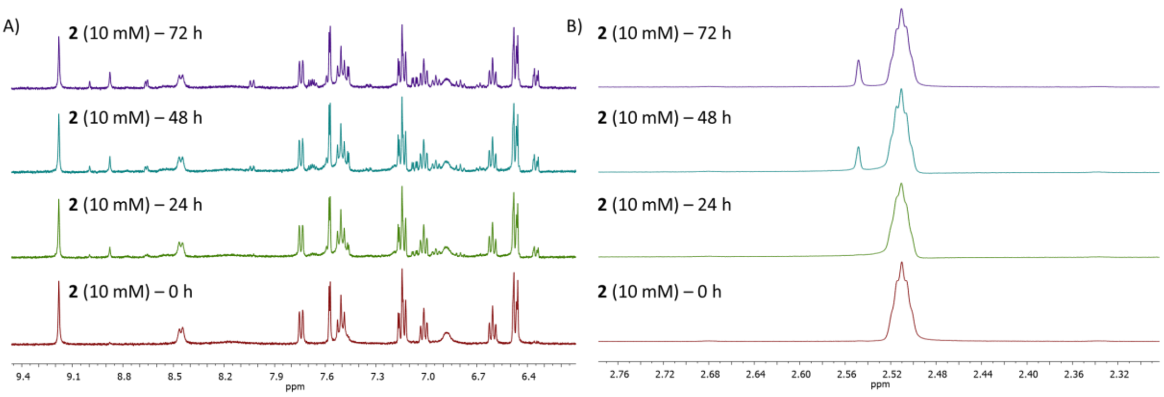

Figure S35. ^{1}H NMR spectra of **2** (10 mM) in D_2O:DMSO-d_6 (9:1) over the course of 72 h. (A) aromatic region and (B) aliphatic region.

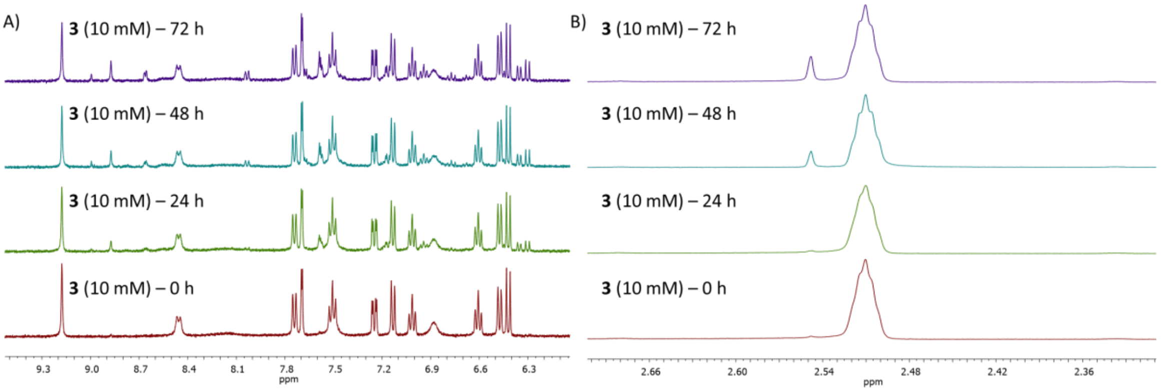

Figure S36. ^{1}H NMR spectra of **3** (10 mM) in D_2O:DMSO-d_6 (9:1) over the course of 72 h. (A) aromatic region and (B) aliphatic region.

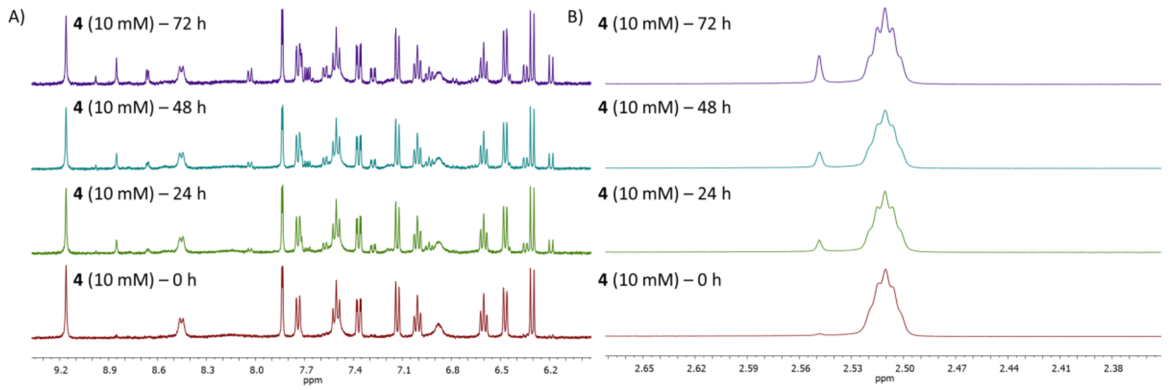

Figure S37. ^{1}H NMR spectra of **4** (10 mM) in D_2O:DMSO-d_6 (9:1) over the course of 72 h. (A) aromatic region and (B) aliphatic region.

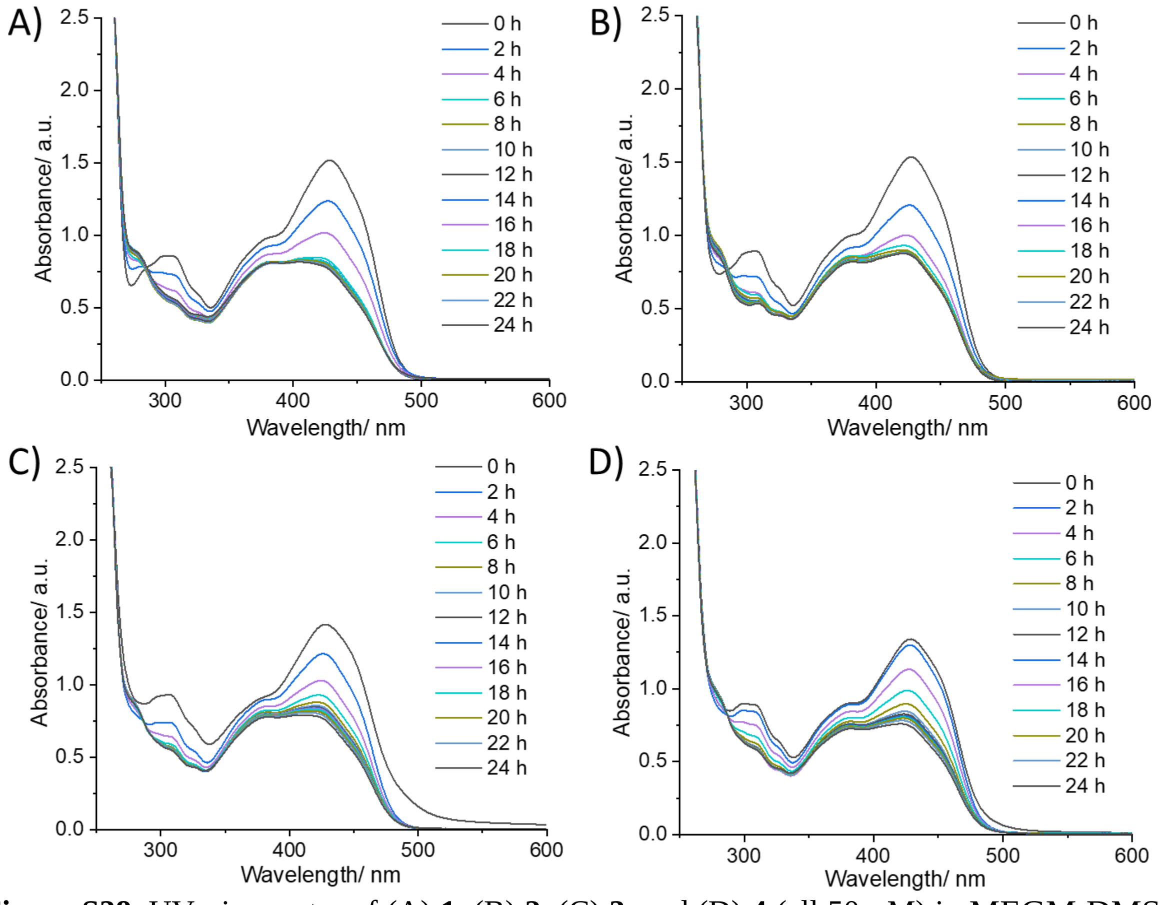

Figure S38. UV-vis spectra of (A) **1**, (B) **2**, (C) **3**, and (D) **4** (all 50 µM) in MEGM:DMSO (200:1**)** over the course of 24 h at 37 °C.

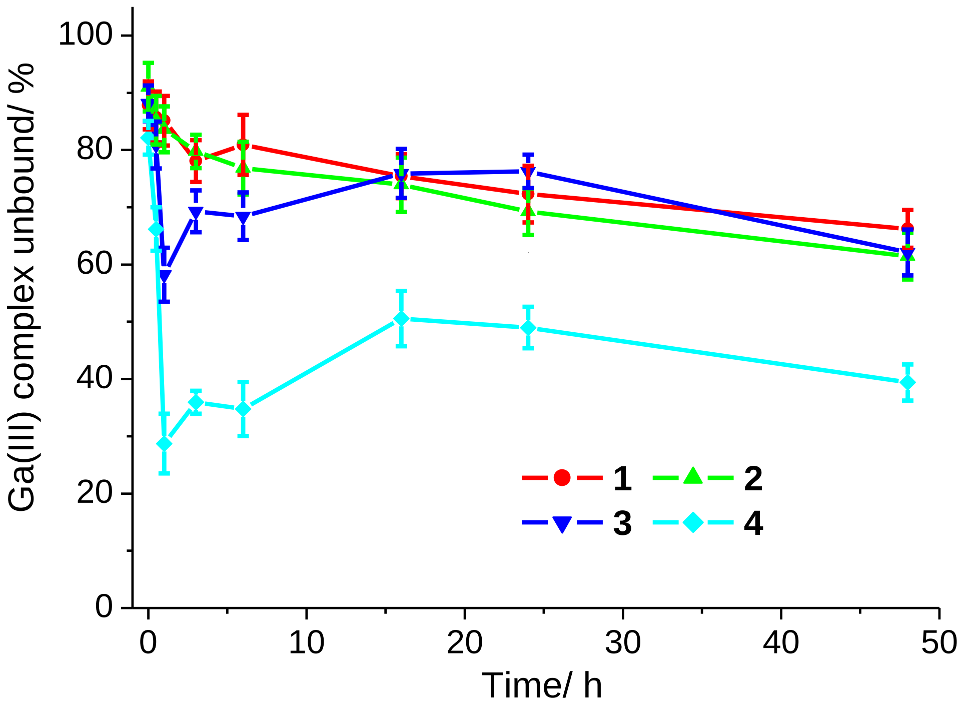

Figure S39. Solution depletion plots showing the relative amount of **1-4** remaining in solution (unbound to hydroxyapatite) over the course of 48 h.

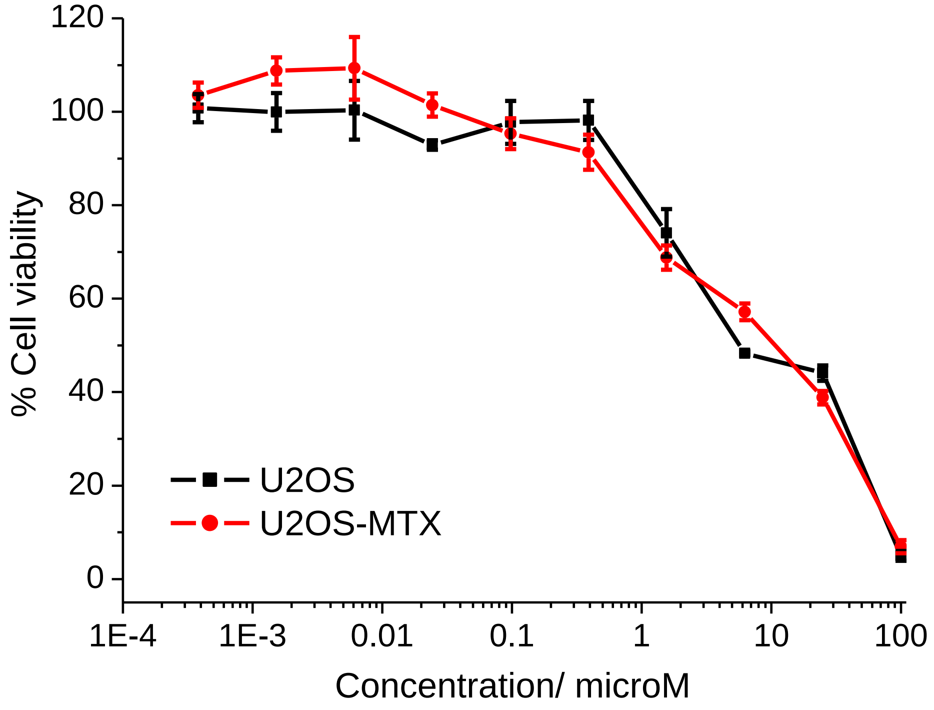

Figure S40. Representative dose-response curves for the treatment of U2OS and U2OS-MTX cells with **1** after 72 h incubation.

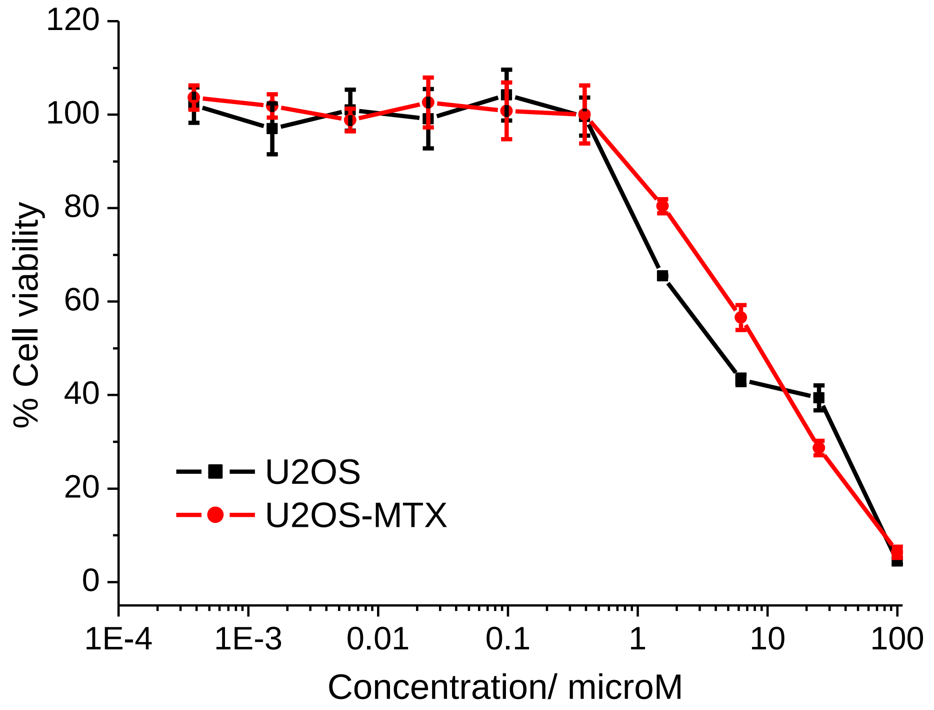

Figure S41. Representative dose-response curves for the treatment of U2OS and U2OS-MTX cells with **2** after 72 h incubation.

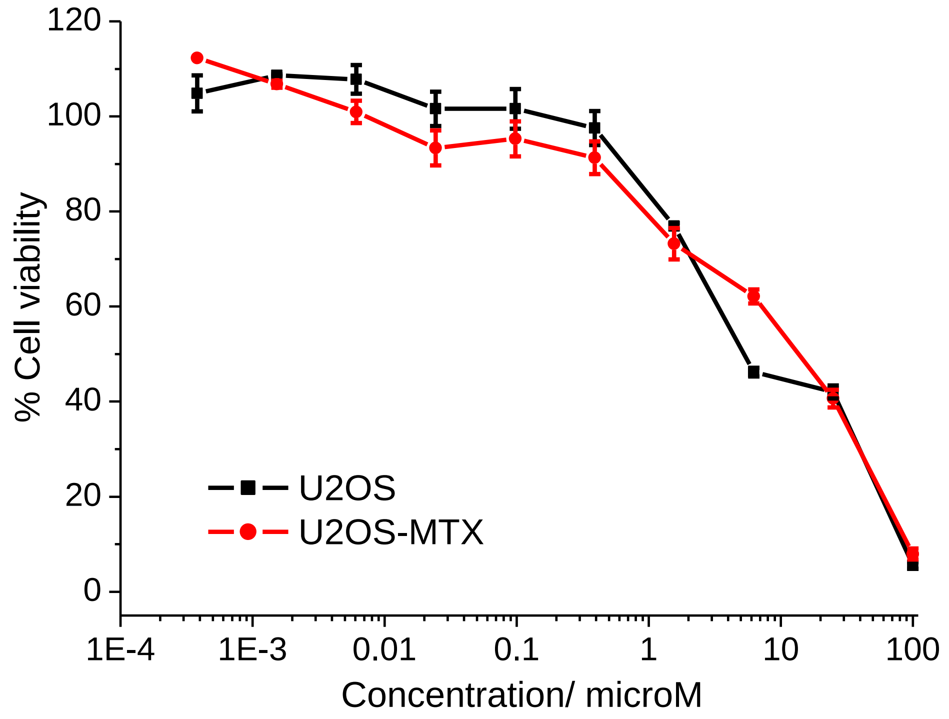

Figure S42. Representative dose-response curves for the treatment of U2OS and U2OS-MTX cells with **3** after 72 h incubation.

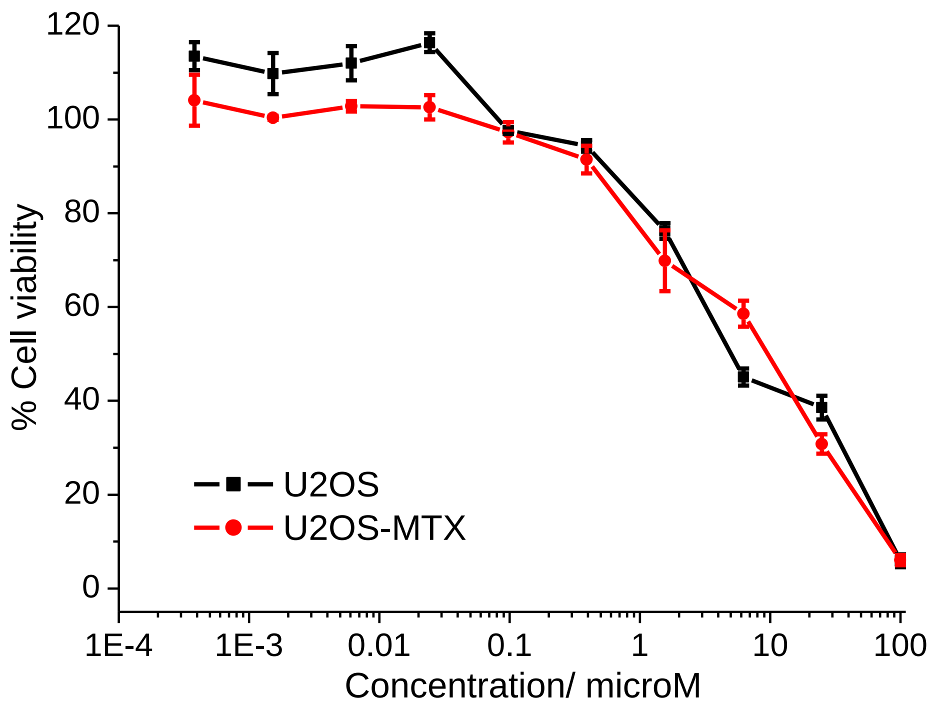

Figure S43. Representative dose-response curves for the treatment of U2OS and U2OS-MTX cells with **4** after 72 h incubation.

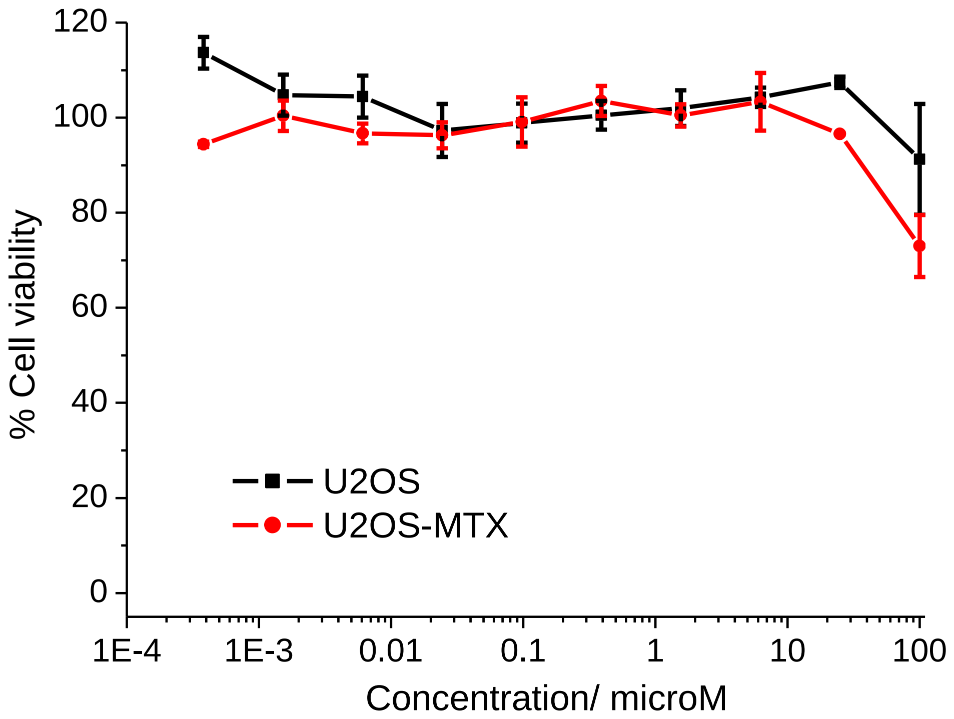

Figure S44. Representative dose-response curves for the treatment of U2OS and U2OS-MTX cells with $\mathbf{L^1}$ after 72 h incubation.

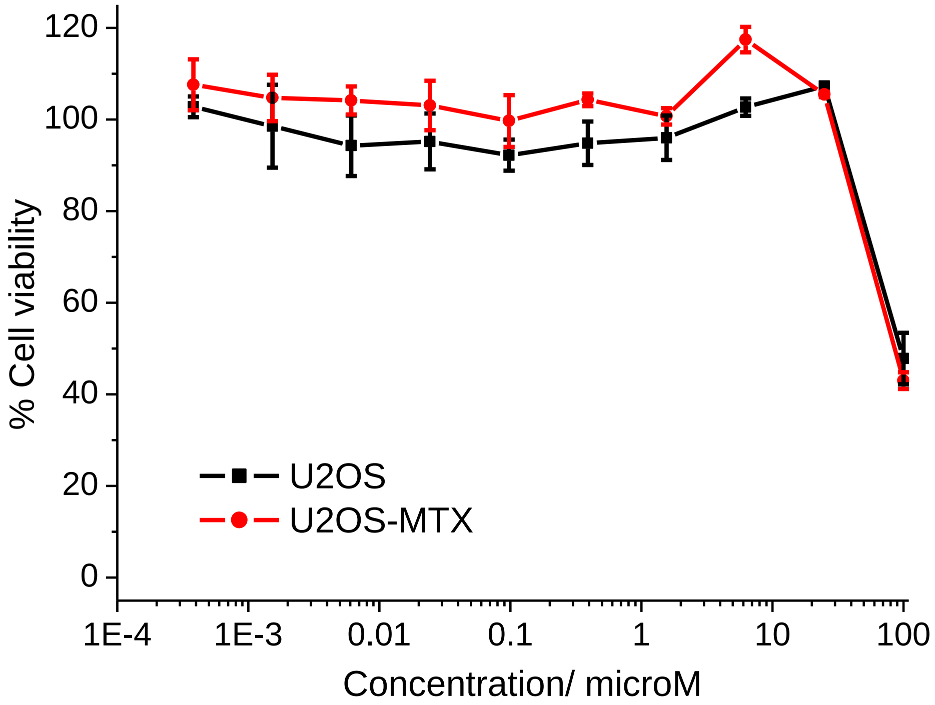

Figure S45. Representative dose-response curves for the treatment of U2OS and U2OS-MTX cells with $\mathbf{L}^2$ after 72 h incubation.

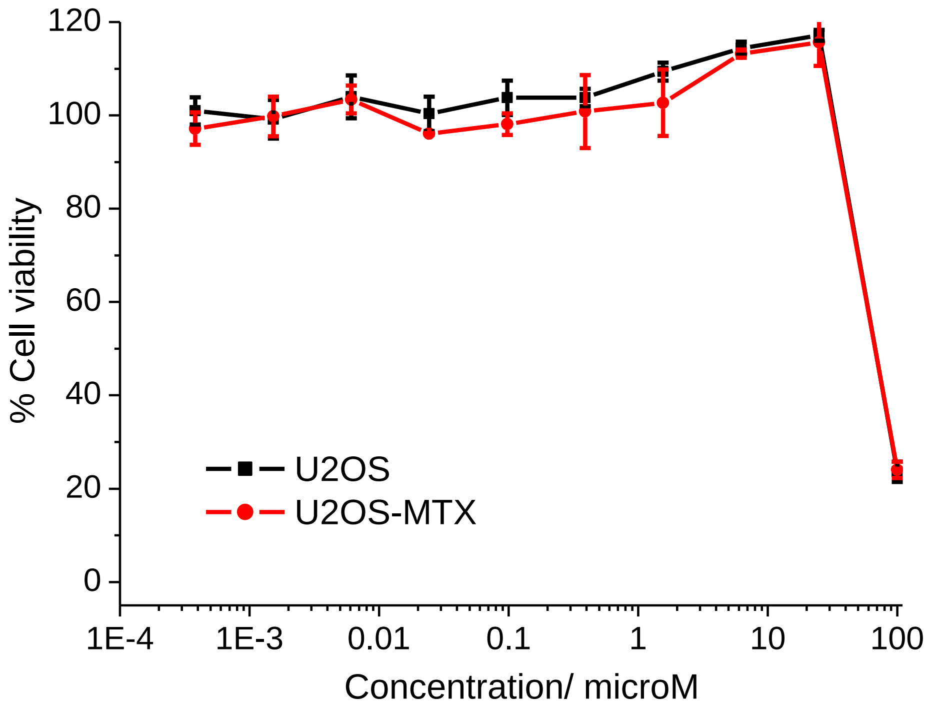

Figure S46. Representative dose-response curves for the treatment of U2OS and U2OS-MTX cells with $\mathbf{L}^3$ after 72 h incubation.

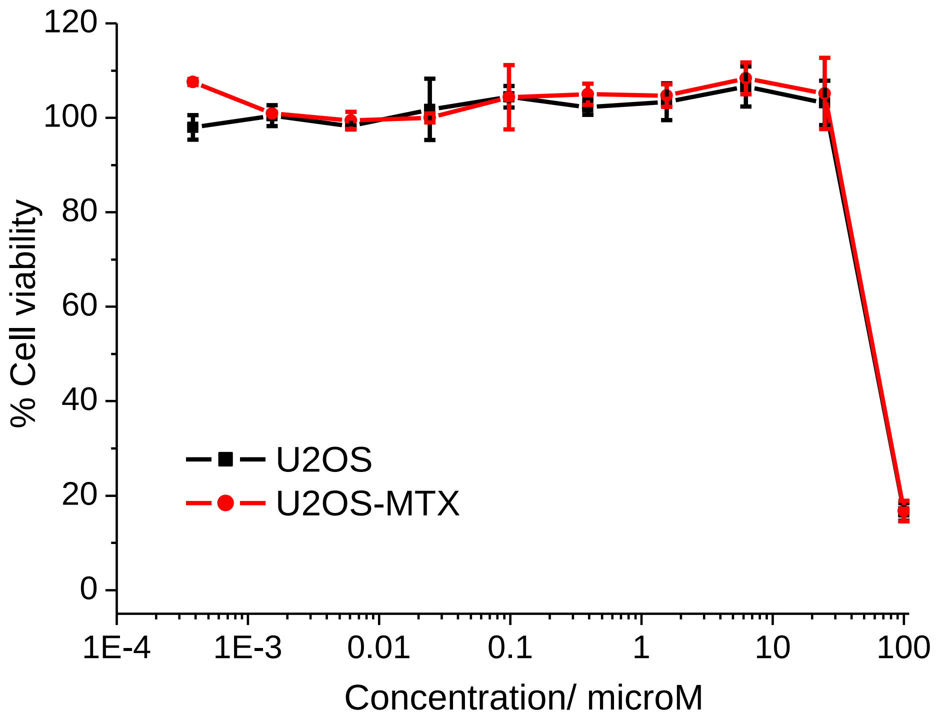

Figure S47. Representative dose-response curves for the treatment of U2OS and U2OS-MTX cells with **L**^{4} after 72 h incubation.

Table S7. IC_{50} values of **L**^{1}-**L**^{4} against U2OS and U2OS-MTX cells. [a] Determined after 72 h incubation (mean of three independent experiments ± SD).

Test compound	U2OS [μM][a]	U2OS-MTX [μM][a]
**L**^{1}	> 100	> 100
**L**^{2}	92.99 ± 9.55	85.67 ± 2.38
**L**^{3}	67.71 ± 1.29	67.47 ± 1.32
**L**^{4}	58.54 ± 1.46	59.33 ± 3.52

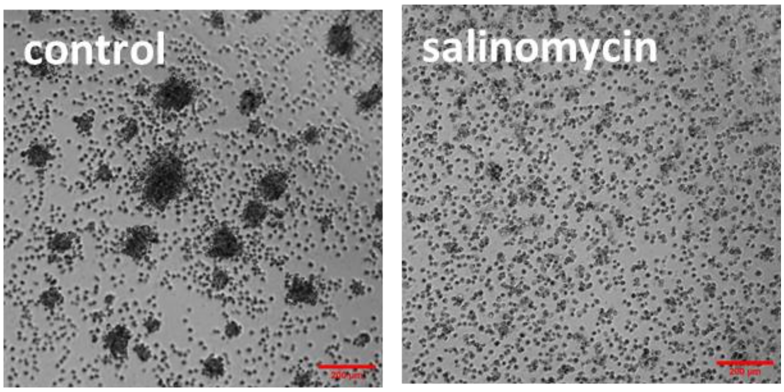

Figure S48. Representative bright-field images (× 10) of U2OS-MTX sarcospheres in the absence and presence of salinomycin at its IC_{20} values for 10 days.

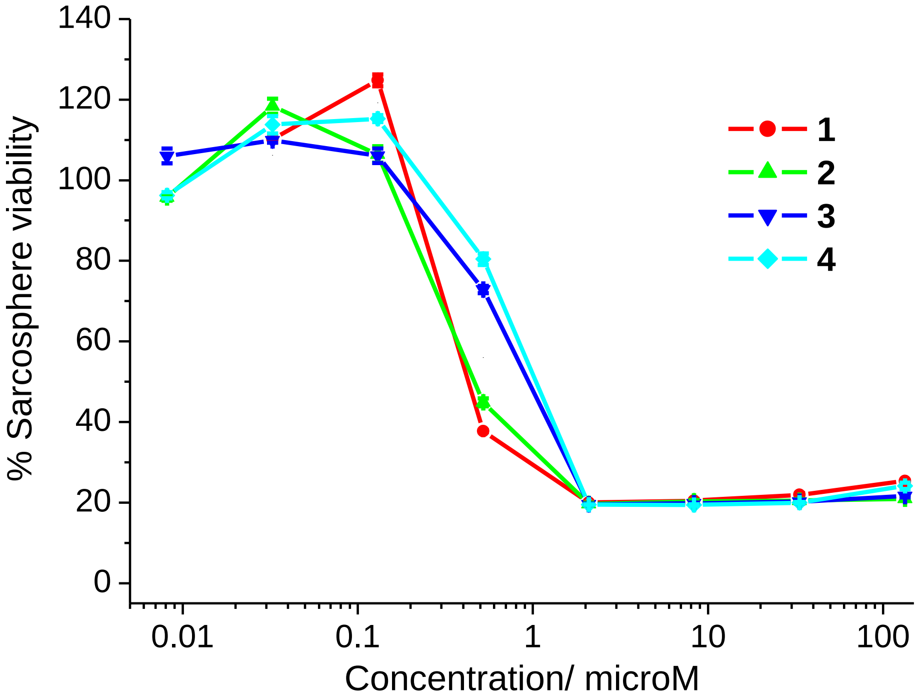

Figure S49. Representative dose-response curves for the treatment of U2OS-MTX sarcospheres with **1-4** after 10 days incubation.

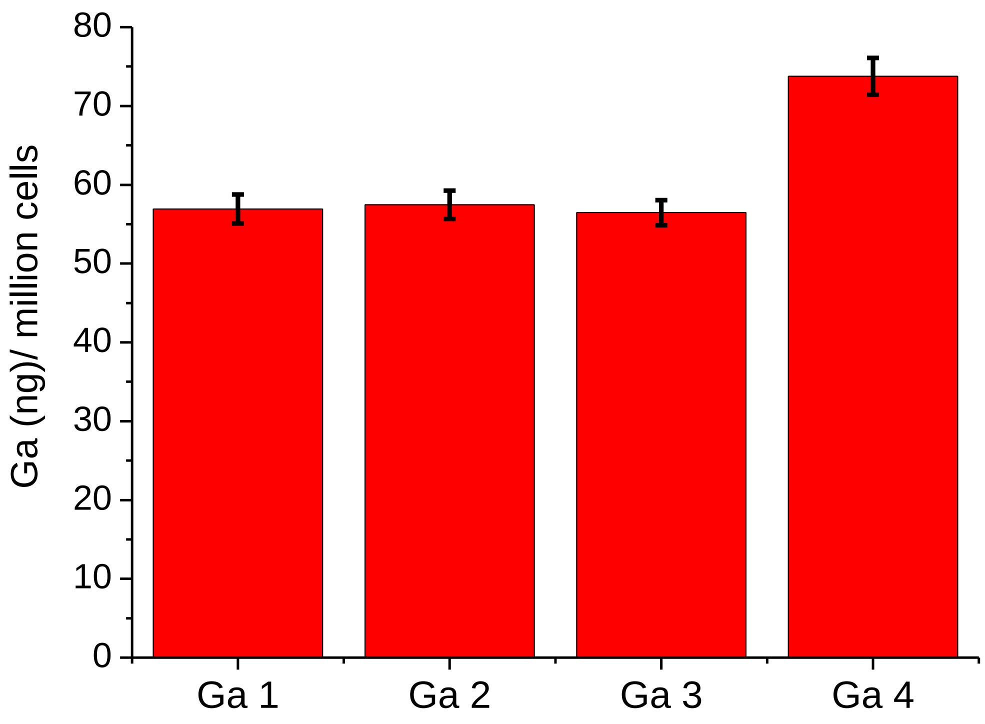

Figure S50. The amount of gallium (in terms of ng of Ga/ million cells) present in U2OS cells treated with **1-4** (5 µM for 24 h).

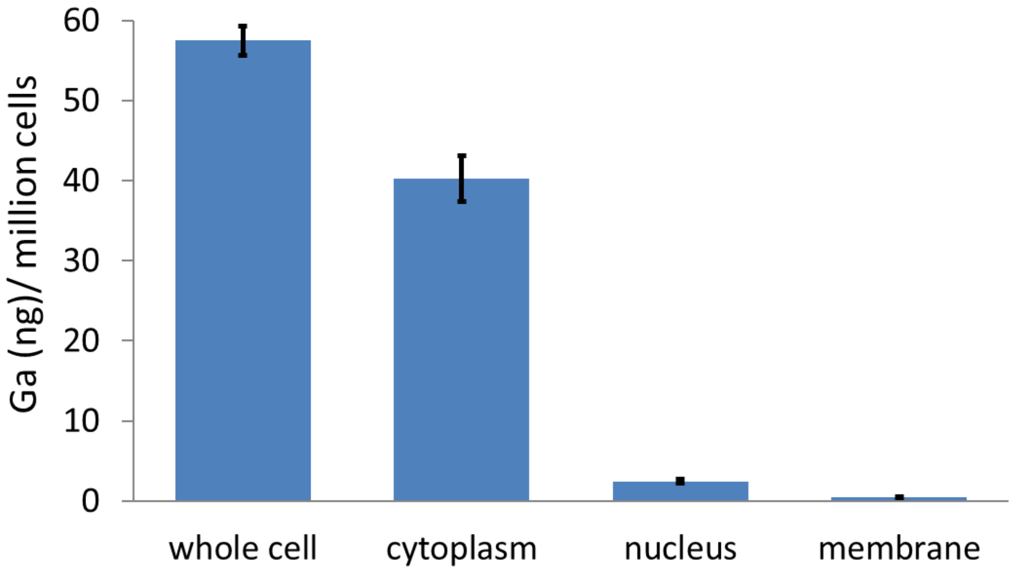

Figure S51. Gallium content (ng of Ga/ 10^6 cells) in various cellular components upon treatment of U2OS cells with **2** (5 µM for 24 h).

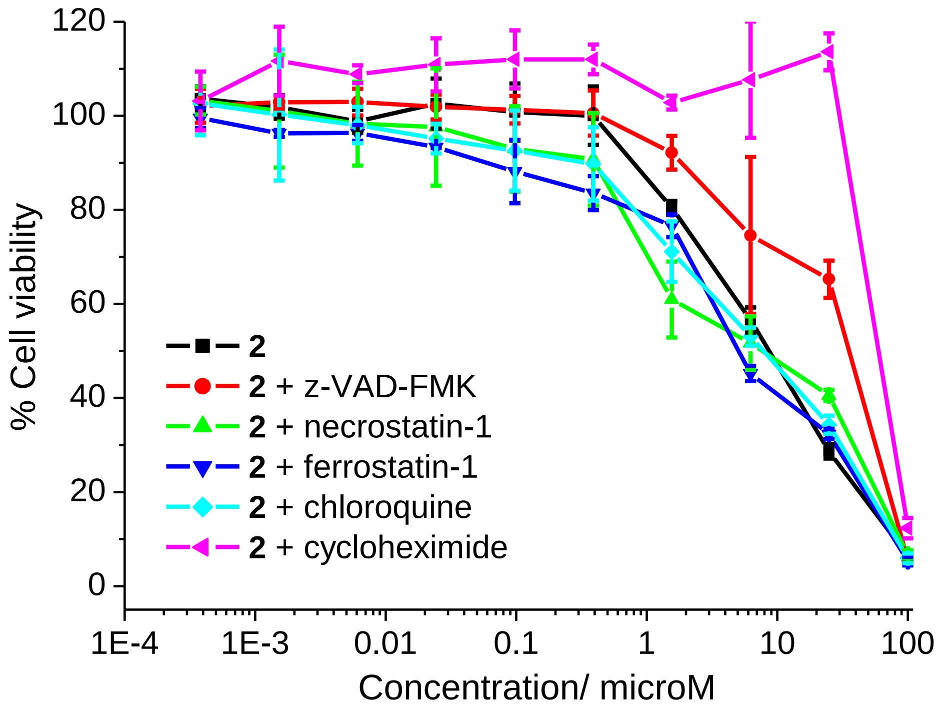

Figure S52. Representative dose-response curves for the treatment of U2OS cells with **2** in the presence of z-VAD-FMK (5 µM), necrostatin-1 (20 µM), ferrostatin-1 (10 µM), chloroquine (10 µM) or cycloheximide (1 µM) after 72 h incubation.

Table S8. IC_{50} values of **2** against U2OS in the absence and presence of apoptosis (z-VAD-FMK, 5 µM), necroptosis (necrostatin-1, 20 µM), ferroptosis (ferrostatin-1, 10 µM), autophagy (chloroquine, 10 µM), and paraptosis (cycloheximide, 1 µM) inhibitors. [a] Determined after 72 h incubation (mean of three independent experiments ± SD).

Test compound	U2OS [µM][a]
**2**	8.70 ± 0.38
**2** + z-VAD-FMK	35.65 ± 0.35
**2** + necrostatin-1	6.68 ± 1.43
**2** + ferrostatin-1	5.08 ± 0.23
**2** + chloroquine	7.97 ± 0.62
**2** + cycloheximide	59.68 ± 1.05

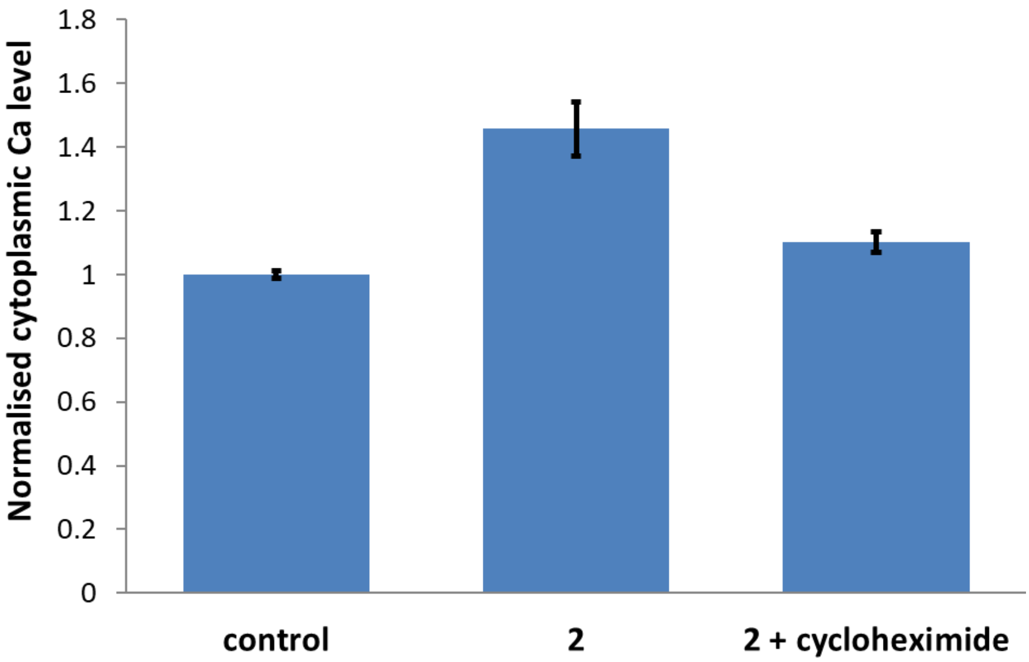

Figure S53. Normalised cytoplasmic calcium levels in U2OS cells untreated and treated with **2** (2 × IC_{50} value for 24 h) or co-treated with **2** (2 × IC_{50} value for 24 h) and cycloheximide (1 µM for 24 h).

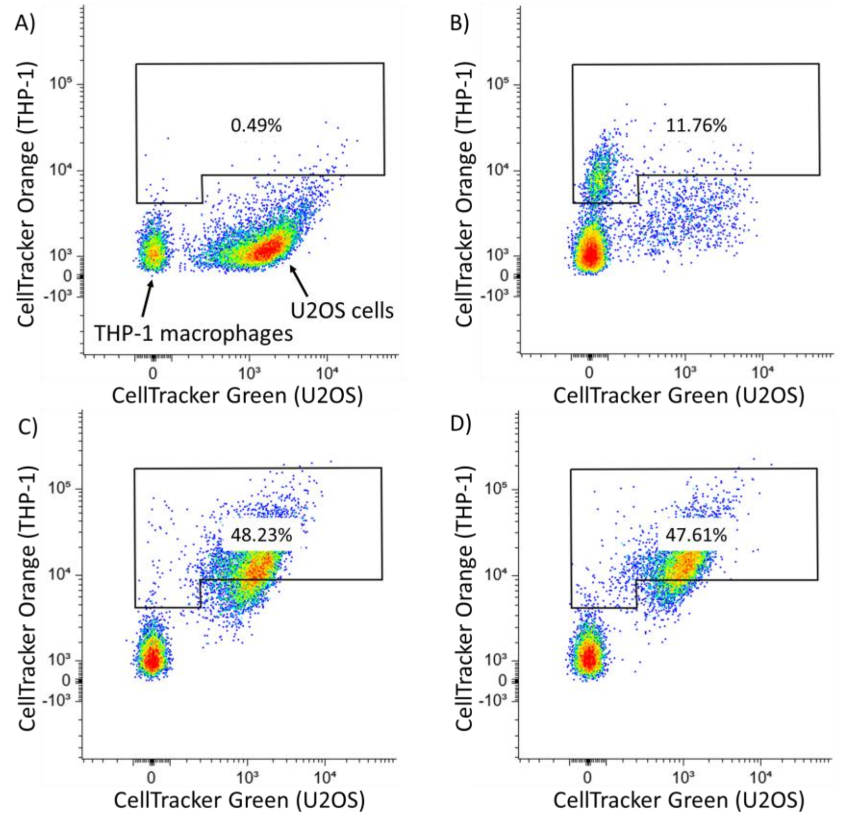

Figure S54. Representative two-dimensional scatter plots of CellTracker Green-stained U2OS cells (A) untreated and treated with (B) cisplatin (150 µM) and thapsigargin (7 µM) or (C) **2** (25 µM) or (D) **2** (50 µM) for 24 h and then co-cultured with CellTracker Orange-stained THP-1 macrophages for 2 h.

References

1. G.M. Sheldrick, *Program for Area Detector Absorption Correction*, Institute for Inorganic Chemistry, University of Göttingen: Göttingen, Germany, 1996.
2. G. M. Sheldrick, *Acta Crystallogr. Sect. A*, 2008, **64**, 112-122.
3. G. M. Sheldrick, *Acta Crystallogr. Sect. C*, 2015, **71**, 3-8.
4. O. V. Dolomanov, L. J. Bourhis, R. J. Gildea, J. A. K. Howard, H. Puschmann, *J. Appl. Crystallogr.*, 2009, **42**, 339-341.
5. P. Robin, K. Singh and K. Suntharalingam, *Chem. Commun.*, 2020, **56**, 1509-1512.
